# Engineering Dimensional Configuration of Single‐Atom S‐Cu‐S Sites as Reversible Electron Station for Enhanced Peroxidase‐Mimicking

**DOI:** 10.1002/advs.202510133

**Published:** 2025-10-03

**Authors:** Wenjie Ma, Qian He, Jiancheng Sun, Yiqing Chen, Hongfei Su, Ludan Zhang, Xiao He, Yuguang Wang, Changjian Xie, Zhiyong Zhang, Xin Zhou, Yuliang Zhao, Wenyan Yin

**Affiliations:** ^1^ CAS Key Laboratory for Biomedical Effects of Nanomaterials and Nanosafety & CAS Center for Excellent in Nanoscience Institute of High Energy Physics and National Center for Nanoscience and Technology of China Chinese Academy of Sciences Beijing 100049 China; ^2^ College of Veterinary Medicine Institute of Comparative Medicine Yangzhou University Yangzhou 225009 China; ^3^ School of Light Industry and Food Engineering Guangxi University Nanning 530004 China; ^4^ Jinan Laboratory of Applied Nuclear Science Jinan 250131 China; ^5^ Center of Digital Dentistry Peking University School and Hospital of Stomatology & National Center of Stomatology Beijing 100081 China; ^6^ School of life Sciences and medicine Shandong University of Technology Zibo 255000 China

**Keywords:** biomimetic catalysis, dimensional configuration, neighboring metal site, peroxidase‐like activity, single‐atom nanozymes

## Abstract

Boosting catalytic activity of single‐atom nanozymes (SAzymes) to substitute natural metalloenzymes remains challenging due to the lack of enzyme‐like secondary building blocks and proper 3D conformation. Herein, a natural amino acid L‐cysteine (L‐Cys)‐triggered auto‐assembly process engineers the spatial positioning of 3D‐biomimetic S‐Cu‐S single‐atom catalytic sites and adjacent L‐Cys on sheet‐like MoS_2_ nanozyme, achieving activated MoCC SAzymes. MoCC achieves a maximum Cu single‐atom loading of 10.11% by suppressing aggregation through L‐Cys coordination. Particularly, MoCC can properly bind and react with the H_2_O_2_ substrate, mimicking 3D catalytic pockets of natural enzymes. The maximum reaction velocity (4.56×10^−7^ M s^−1^), affinity (Michaelis constant, 0.65 mM), and specific activity (SA) (355.59 U mg^−1^) catalyzed by peroxidase (POD)‐mimicking MoCC are 16.3‐, 17.9‐, and 1.2‐fold higher than natural horseradish peroxidase (HRP). Density functional theory computations reveal that the S‐Cu‐S single‐atom catalytic sites stabilized by L‐Cys bonding function as a reversible electron flow workstation, triggering storage and transfer with MoS_2_, facilitating swift electron exchange with H_2_O_2_, reducing energy barrier for hydroxyl radicals generation. The optimized 3D S‐Cu‐S single‐atom featuring L‐Cys building of MoCC exhibits cascaded catalase‐like activity and sono‐piezocatalysis effect, non‐invasively amplifying the generation of oxygen and singlet oxygen. Consequently, multiple free radicals can selectively eliminate dental bacteria and biofilms.

## Graphical abstract

L‐cysteine triggers auto‐assembly of POD‐like 3D biomimetic S‐Cu‐S single‐atom nanozymes on MoS_2_ (MoCC). MoCC shows 16.3‐fold higher catalytic velocity and 17.9‐fold greater affinity than HRP, enabling efficient •OH generation via enhanced electron inversion and transfer. Its cascaded catalase‐like/sono‐piezocatalytic activity produces O_2_/^1^O_2_, generating targeted ROS storm for anti‐caries and teeth whitening.

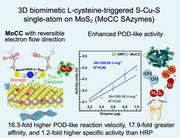

## 1 Introduction

Nanozymes are a category of nanomaterials that combine the advantages of natural enzymes with chemical catalysts, overcoming deficiencies of natural enzymes, including easy inactivation, high cost, and difficult storage.^[^ ^1^, ^2^, ^3^ ^]^ Among these, redox nanozymes can mimic metalloenzymes like peroxidase (POD), oxidase (OXD), and catalase (CAT), enabling precise reactive oxygen species (ROS) regulation for various disease diagnosis and treatment.^[^ ^4^, ^5^, ^6^ ^]^ The emerging metal single‐atom nanozymes (SAzymes) have received great attention as innovative biocatalysts for combating bacterial infections.^[^ ^7^, ^8^, ^9^ ^]^ SAzymes exhibit superior catalytic activity in contrast to conventional nanozymes because of their maximum atomic utilization and explicit electronic and geometric structures.^[^ ^10^, ^11^, ^12^ ^]^ Importantly, the metal active sites of SAzymes can be finely tuned by manipulating metal‐support interaction, structure‐activity relationship, and tailoring physicochemical performances like surface modification, coordination numbers and elements, defects, etc.^[^ ^13^, ^14^ ^]^ For instance, SAzymes have heightened POD‐like activity and kinetics far exceeding nanozymes counterpart to generate hydroxyl radicals (•OH) with strong oxidizing property by catalyzing H_2_O_2_ for efficient bacteria elimination.^[^ ^15^, ^16^ ^]^ However, achieving POD‐like activity comparable to that of natural enzymes and satisfactory selectivity under complex physiological conditions remains a long‐term goal for SAzymes.

In particular, the majority of active metal centers of SAzymes are planar structures with high surface energy, prone to aggregation/leaching when synthesized by common pyrolysis methods.^[^ ^17^ ^]^ This hinders their synergistic binding function with the substrates, ultimately limiting their catalytic activity. In contrast, natural enzymes, with intricate 3D configurations in catalytic pockets, harness non‐covalent forces between surrounding amino acids and active sites to facilitate swift binding and release of substrates, while stabilizing transition states during the reactive pathway.^[^ ^18^, ^19^ ^]^ Therefore, as essential secondary building blocks of natural enzymes, amino acids are crucial for the synergy in the catalysis activity and structural stability.^[^ ^20^ ^]^ Electron transport is the fundamental principle underlying catalysis‐related redox reactions occurring in either nanozymes or natural enzymes.^[^ ^21^ ^]^ However, the development of high enzyme‐mimicking activities of SAzymes through engineering their 3D‐biomimetic configuration, incorporating the nearby amino acids to accelerate electron transfer, remains an underexplored strategy.

In living species, Cu element with a variable valence state plays a key role in maintaining homeostasis. Cu‐containing natural enzymes are a significant portion of metalloenzymes, such as cytochrome C oxidase, superoxide dismutase, etc.^[^ ^22^, ^23^, ^24^ ^]^ Cu SAzymes, with their extraordinary multienzyme‐like behaviors and biocompatibility, can catalyze the oxidation of biomolecules such as glutathione (GSH), proteins, nucleic acids and lipids of bacterial cells, thereby inducing bacterial apoptosis and elimination.^[^ ^25^, ^26^, ^27^, ^28^ ^]^ Especially, Cu (I)‐based SAzymes can participate in redox cycling with GSH, which facilitates H_2_O_2_ generation while reducing the scavenging effect of GSH on ·OH.^[^ ^29^ ^]^ Despite these advancements, the development of Cu SAzymes in antibacterial therapy still encounters numerous bottlenecks. First, more suitable substrates need to be selected for Cu single atoms to enhance catalytic activity in the bacterial microenvironment, while avoiding off‐target effects in normal tissues. Second, the limited Cu single atoms loading ratio hampers further application. Molybdenum disulfide (MoS_2_) nanosheets (NSs) have attracted ever‐growing attention as a transition metal catalyst for various biomedical applications.^[^ ^30^ ^]^ Notably, MoS_2_ exhibits good biocompatibility because Mo is a trace element required by humans.^[^ ^31^ ^]^ Based on their unique advantages such as high near‐infrared photothermal properties and tunable piezoelectric efficiency, MoS_2_ as POD‐like nanozymes have been widely used as antimicrobial alternatives.^[^ ^32^, ^33^, ^34^, ^35^ ^]^ Their POD‐like activity can be improved using defect‐tunable active sites, heteroatom doping and photo/ultrasound (US) stimulus for accelerated electron transport.^[^ ^32^, ^36^, ^37^, ^38^ ^]^ Particularly, owing to their extensive specific surface area, easy modification, and multiple enzyme‐like activity, MoS_2_ NSs are promising as a support in SAzymes.^[^ ^39^, ^40^, ^41^ ^]^ Inspired by the features of MoS_2_ and the 3D catalytic pocket in natural enzymes, we hypothesized that engineering configuration of highly loaded 3D Cu single‐atom catalytic centers on the MoS_2_ support could boost electron transport for enhanced POD‐like activity.

**Scheme 1:**
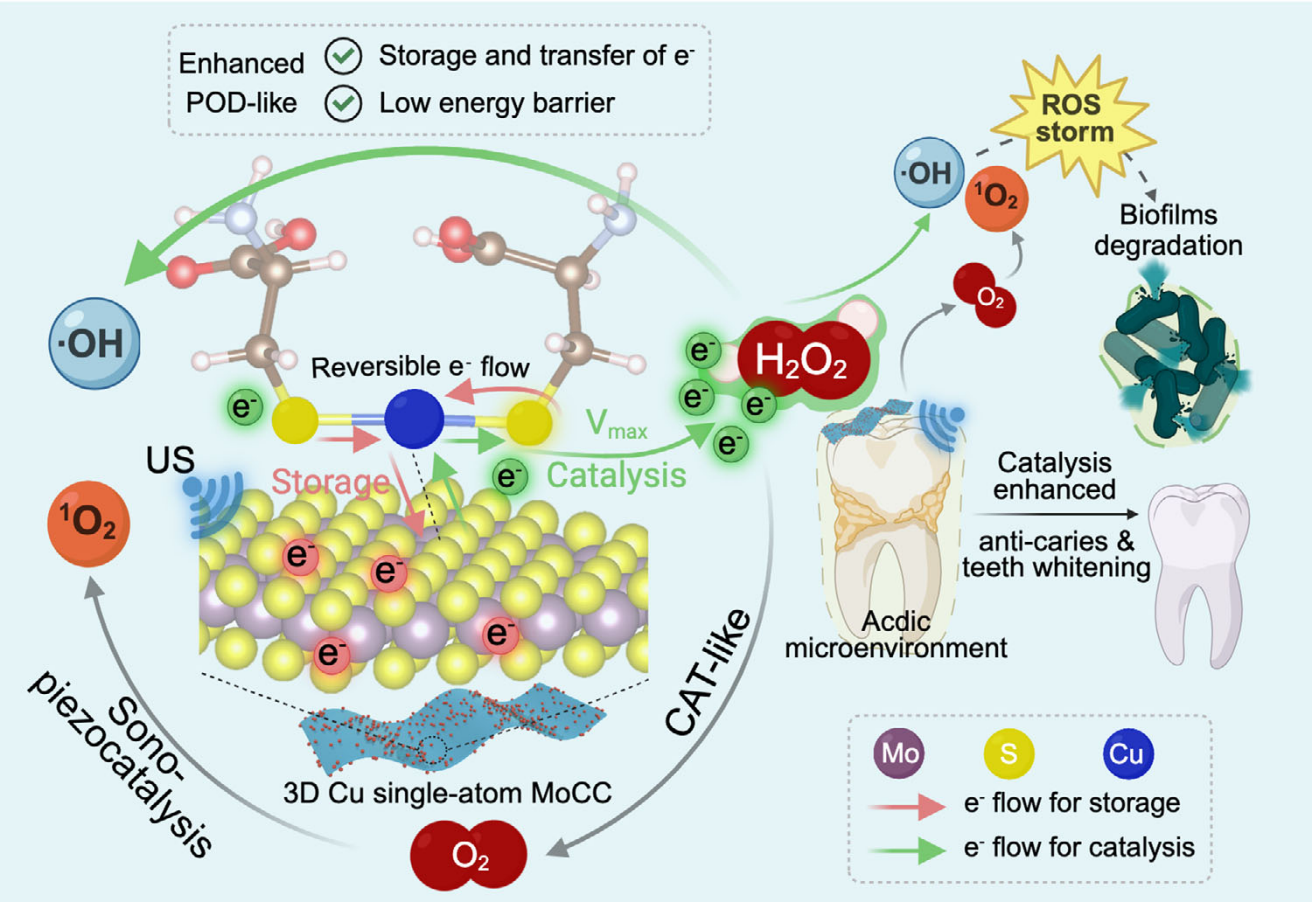
Schematic illustration of L‐Cys triggered auto‐assembly of 3D Cu single‐atom with S‐Cu‐S catalytic and L‐Cys bonding sites on MoS_2_, forming MoCC SAzymes. The S‐Cu‐S catalytic pocket acts as a unique reversible electron flow workstation, where e^−^ are donated to MoS_2_ for storage and are reversibly transferred as needed for S‐Cu‐S bridge catalysis, enabling H_2_O_2_ to quickly exchange e^−^, reducing the energy barrier to enhance POD‐like activity and converting H_2_O_2_ into ·OH, with the catalytic performance comparable to natural HRP. Simultaneously, cascaded CAT‐like activity and sono‐piezocatalysis effect of MoCC produce US‐amplified O_2_ and ^1^O_2_. Meanwhile, the acidic oral microenvironment‐responsive ROS storm can catalytically degrade biofilm extracellular polymeric substances (EPS) and kill bacteria to prevent dental caries and to whiten teeth.

In this study, we engineered a biomimetic Cu single‐atom MoS_2_ nanozyme, denoted as MoCC SAzymes, in which the single S‐Cu‐S site adjacent to L‐cysteine (L‐Cys) was 3D positioned on the plane of MoS_2_ nanozyme via an auto‐assembly process triggered by L‐Cys coordination. By manipulating the ratio of Cys‐Cu‐Cys to MoS_2_, a maximum Cu single‐atom loading ratio of 10.11% was achieved. The tailored MoCC featured the coexistence of well‐dispersed 3D S‐Cu‐S catalytic sites and L‐Cys bonding sites, enabling a reversible and accelerated electron flow to the MoS_2_ support. Particularly, MoCC exhibited a high maximum reaction velocity (V_max_) and affinity for the H_2_O_2_ substrate, about 5.0 and 4.5 times higher than those of MoS_2_. MoCC also exhibited approximately 16.3 times higher V_max_, 17.9 times greater affinity, and 1.2 times higher specific activity (SA) than those of natural horseradish peroxidase (HRP) for H_2_O_2_. Density functional theory (DFT) computations demonstrated that the 3D S‐Cu‐S with L‐Cys bonding sites served as a reversible electron flow workstation, for electron storage and rapid transfer, with the Cu single‐atom donating electrons to MoS_2_. The S and Cu atoms in the L‐Cys‐stabilized 3D catalytic pocket of MoCC work together like an electronic bridge to enable H_2_O_2_ to rapidly obtain or release electrons as demanded, reducing the energy barrier of POD‐like activity and converting low‐dose H_2_O_2_ into ·OH. Interestingly, MoCC also exhibited an enhanced sono‐piezocatalysis effect compared to MoS_2_ alone, which was attributed to the high loading ratio of single S‐Cu‐S atom sites. In this case, cascaded CAT‐like activity and sono‐piezocatalysis effect of MoCC triggered the generation of US‐amplified O_2_ and ^1^O_2_ in acidic oral microenvironment. Consequently, the multiple ·OH and ^1^O_2_ achieved effective and selective oral bacteria/biofilm degradation and teeth whitening (**Scheme** **1**). This study presents a unique strategy for designing SAzymes that surpass natural HRP by activating nanozyme and provides valuable insights for enhanced anti‐caries.

## 2 Results and Discussion

### 2.1 Synthesis and Characterizations

**Figure 1:**
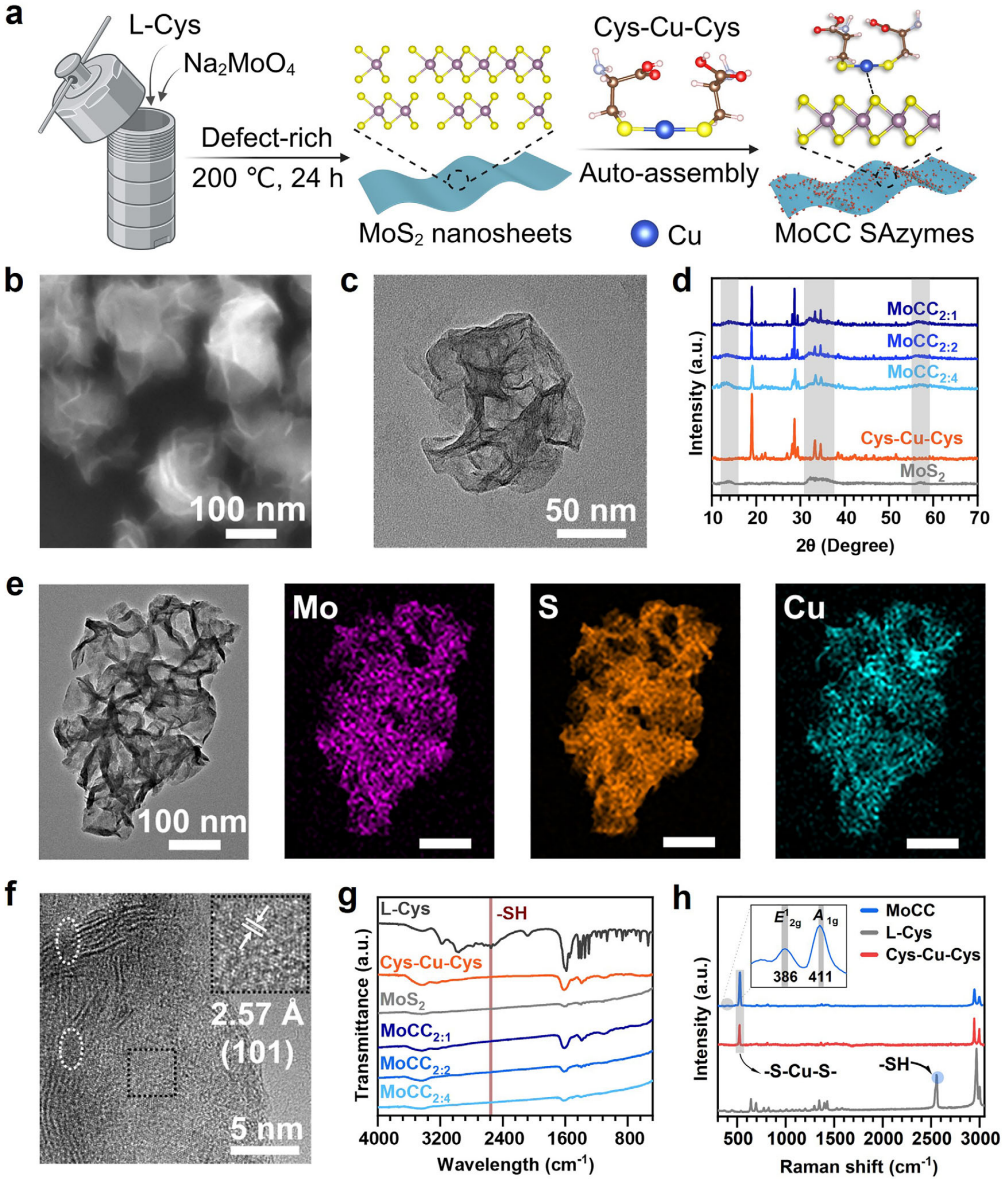
Characterizations of MoS_2_ NSs and MoCC SAzymes. a) Scheme of synthetic procedure of MoCC. b) SEM and c) TEM images of MoS_2_. d) XRD patterns of MoS_2_, Cys‐Cu‐Cys, and MoCC with different Cu single atoms loading ratios. e) Elemental mappings of Mo, S, and Cu signals corresponding to TEM image of MoCC. f) HR‐TEM image of MoCC with surface vacancies highlighted in white circles (inset was the enlarged HRTEM of black dotted box area). g) FT‐IR spectra of L‐Cys, Cys‐Cu‐Cys, MoS_2_, and MoCC with different Cu loading ratios. h) Raman spectra of MoCC, Cys‐Cu‐Cys, and L‐Cys.

MoCC SAzymes were successfully synthesized using a facile coordination‐driven auto‐assembly process (**Figure** **1**a). Initially, MoS_2_ NSs were obtained by a simple hydrothermal method at 200 °C for 24 h. Subsequently, Cu single atoms were auto‐assembled on the surface of MoS_2_ NSs via a natural amino acid L‐Cys coordination‐triggered auto‐assembly strategy to form MoCC SAzymes (Figure 1a). During the auto‐assembly process, ‐SH from L‐Cys coordinated with an appropriate amount of CuCl_2_, forming a Cys‐Cu‐Cys complex (Figure S1, Supporting Information). Then, a stabilized 3D spatial arrangement comprising S‐Cu‐S catalytic sites and L‐Cys bonding sites was auto‐assembled onto the MoS_2_ support to create MoCC SAzymes, mimicking the structure of natural HRP. As demonstrated by scanning electron microscopy (SEM) and transmission electron microscopy (TEM) images, MoS_2_ exhibited wrinkled and twisted sheet‐like structure with an average diameter of 320 nm (Figure 1b,c). MoCC with varying amounts of Cu loading were obtained by adjusting the molar ratio of Cys‐Cu‐Cys to MoS_2_ (Cys‐Cu‐Cys:MoS_2_ = 2:1, 2:2, 2:4) with the corresponding names of MoCC_2:1_, MoCC_2:2_, and MoCC_2:4_. The loading ratios of Cu atoms on MoCC_2:1_, MoCC_2:2_, and MoCC_2:4_, tested by inductively coupled plasma mass spectrometry (ICP‐MS), were 16.51%, 10.11%, and 7.76%, respectively (Figure S2a, Supporting Information). Despite the increase in Cu loading ratio, the morphology of MoCC_2:1_, MoCC_2:2_, and MoCC_2:4_ remained unchanged as shownin the SEM images (Figure S2b–d, Supporting Information), which further emphasized the significance of L‐Cys in enhancing the Cu atom loading ratio. However, for MoCC_2:1_, the Cu atoms lost their monodisperse state due to the insufficient utilization of support space. Therefore, the maximized Cu single atoms loading ratio was 10.11% in the MoCC_2:2_, which was abbreviated as MoCC in the following experiments. X‐ray diffraction (XRD) pattern of MoS_2_ (Figure S3a, Supporting Information) can be indexed as a mixture phase of hexagonal 2H‐MoS_2_ (JPCDS 37–1492) and 1T‐MoS_2_ (JCPDS 75–1539).^[^ ^42^ ^]^ Moreover, the peak of Cys‐Cu‐Cys was attributed to cystine rather than cysteine, indicating the successful coordination of Cu ions with L‐Cys and the formation of S‐Cu‐S group (Figure S3b, Supporting Information).^[^ ^43^ ^]^ The formation of mixed 2H/1T phase was observed in MoCC_2:1, 2:2, 2:4_ (grey‐shaded area in Figure 1d), indicating a poorly crystalline MoS_2_ structure. In addition, distinct diffraction peaks at approximately 19°, 29°, 33°, and 34° were observed in MoCC_2:1, 2:2, 2:4_, which could be attributed to the coordination of Cys‐Cu‐Cys, with no significant peak shift being observed. Particularly, no additional XRD diffraction peaks related to Cu compounds or metallic Cu were found. The presence of Mo, S, and Cu elements in MoCC was confirmed by energy dispersive spectroscopy (EDS) (Figure S4, Supporting Information). MoCC exhibited a morphology similar to MoS_2_ but typically had a uniformly and atomically distributed Cu element, which was further confirmed by TEM image with elemental mappings (Figure 1e). A high‐resolution TEM (HR‐TEM) image revealed that MoCC had a multilayered structure with interplanar spacings of the (101) plane of 2.57 Å and rich surface vacancies (Figure 1f). Selected area electron diffraction (SAED) demonstrated the polycrystalline structure of MoCC (Figure S5, Supporting Information). Atomic force microscopy (AFM) images indicated that the multilayered MoCC had a thickness of 11.3 nm (Figure S6, Supporting Information). Moreover, the zeta potential underwent significant changes from ‐72.2 mV (MoS_2_) to +37.6 mV (Cys‐Cu‐Cys), and finally to ‐33.8 mV (MoCC), demonstrating the successful auto‐assembly of Cu single atoms to MoS_2_ support (Figure S7, Supporting Information). To evaluate the dispersion stability, MoS_2_ and MoCC were measured by dynamic light scattering (DLS) analysis. In Figure S8 (Supporting Information), similar mean diameters of MoS_2_ and MoCC were confirmed in water, indicating their superior dispersion stability. Fourier Transform Infrared (FT‐IR) spectra (Figure 1g) showed that characteristic ‐SH at 2552 cm^−1^ of L‐Cys disappeared, indicating its transition into S‐Cu‐S bond of Cys‐Cu‐Cys in MoCC_2:1, 2:2, 2:4_.^[^ ^24^ ^]^ In contrast to the Raman spectrum of L‐Cys, the appearance of new peaks of MoCC corresponding to the S─Cu─S bond further proved the efficient auto‐assembly of Cu single atoms. In addition, the Raman spectra of MoCC (Figure 1h) displayed notable shifts in the *A*_1g_ (411 cm^−1^) and *E* ^1^_2g_ (386 cm^−1^) peaks compared to MoS_2_ NSs alone (*A*_1g_ 402 cm^−1^ and *E* ^1^_2g_ 378 cm^−1^) (Figure S9, Supporting Information).^[^ ^44^ ^]^ In Cys‐Cu‐Cys a characteristic S‐Cu–S vibrational peak at 522.44 cm^−1^ was observed, which was preserved and blue‐shifted to 527.30 cm^−1^ in MoCC (Figure 1h). This indicated coordination retention and possible charge redistribution when Cys‐Cu‐Cys interacts with MoS_2_ in MoCC.

**Figure 2:**
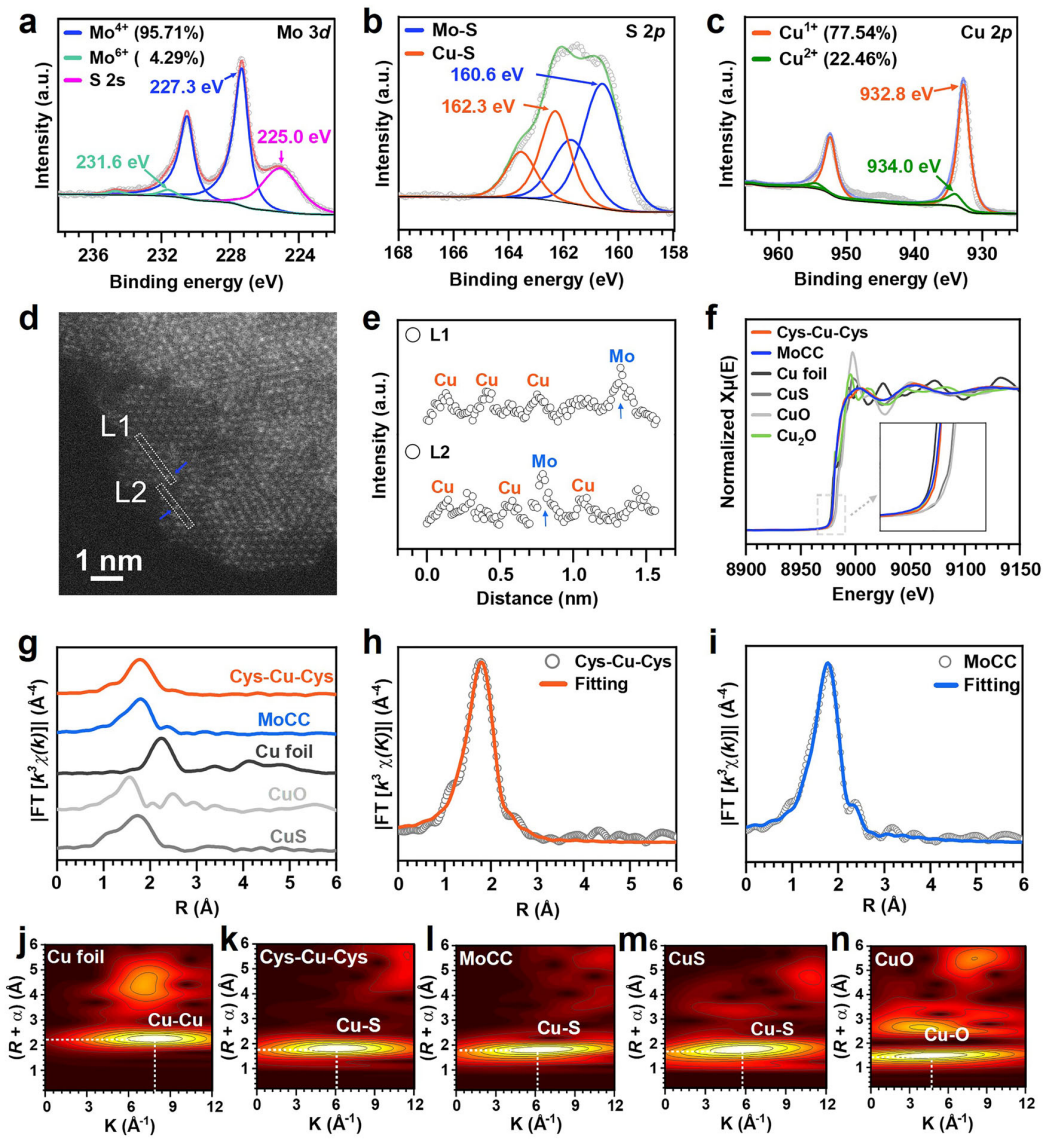
Characterizations of element valences and coordination environment of MoCC SAzymes. XPS spectra of a) Mo 3*d*, b) S 2*p*, and c) Cu 2*p* of MoCC. d) AC‐STEM of MoCC (blue arrows: Mo atom). e) Intensity profiles taken along two lines (L1, L2) in (d). f) XANES spectra of Cu *K*‐edge in MoCC, Cys‐Cu‐Cys, Cu foil, CuO, Cu_2_O, and CuS. g) Fourier transform (FT)‐EXAFS curves of Cu *K*‐edge in MoCC, Cys‐Cu‐Cys, Cu foil, CuO, and CuS. Corresponding *R* space fitting curves of h) Cys‐Cu‐Cys and i) MoCC. j–n) WT analysis of Cu in Cu foil, Cys‐Cu‐Cys, MoCC, CuS, and CuO.

X‐ray photoelectron spectroscopy (XPS) was further applied to assess the element valences and composition of MoCC SAzymes. In the XPS spectrum of MoCC, distinct peaks attributable to Cu, Mo, S, O, and N elements were detected (Figure S10a, Table S1, Supporting Information). In the Mo *3d* orbitals of MoCC (**Figure** **2**a), doublets located at 227.3 and 230.5 eV were Mo 3*d*_5/2_ and Mo 3*d*_3/2_ of Mo (4+), respectively. The relatively shorter doublets at 231.6 and 234.6 eV were designated to Mo (6+). These weak Mo (6+) signals were originated from the slight oxide Mo species during hydrothermal synthesis.^[^ ^45^ ^]^ The Mo (4+) 3*d*_5/2_ peaks of MoCC shifted to low binding energies by 0.3 eV compared to the MoS_2_ (Figure S10b, Table S1, Supporting Information), implying a slight change in the chemical environment after Cu single atoms loading.^[^ ^46^ ^]^ In the S 2*p* spectrum, the doublets at 160.8 and 162.1 eV corresponded to S 2*p*_3/2_ and S 2*p*_1/2_ of Mo‐S bond within MoS_2_ (Figure S10c, Supporting Information). In MoCC, the Mo─S bond was also observed, with a slight shift of 0.2 eV to low binding energy compared to MoS_2_ (Figure 2b). Notably, in MoCC, the doublets at 162.3 and 163.5 eV of S 2*p* confirmed the S‐Cu‐S bond after the loading of Cu single atoms.^[^ ^47^ ^]^ For Cys‐Cu‐Cys, the Cu 2*p*_3/2_ and 2*p*_1/2_ peaks were at 932.7 eV and 952.6 eV, respectively (Figure S10d, Supporting Information); For MoCC, the Cu 2*p*_3/2_ and 2*p*_1/2_ peaks were at 932.8 eV and 952.4 eV, respectively (Figure 2c). This finding suggested the existence of either Cu (0) or Cu (1+). According to the kinetic energy spectrum, Cu L_3_M_45_M_45_ Auger peaks of Cys‐Cu‐Cys (Figure S10e, Supporting Information) and MoCC (Figure S10f, Supporting Information) were located at 917.0 and 916.5 eV, respectively, suggesting that the Cu (0) (LMM at 918.7 eV^[^ ^48^ ^]^) was not detected.^[^ ^49^ ^]^ In MoCC, 934.0 and 954.6 eV corresponded to Cu (2+) 2*p*_3/2_ and Cu (2+) 2*p*_1/2_, respectively.^[^ ^43^, ^50^ ^]^ Consequently, the proportion of Cu (1+) decreased from 85.65% in Cys‐Cu‐Cys to 77.54% in MoCC, while Cu (2+) increased from 14.35% in Cys‐Cu‐Cys to 22.46% in MoCC (Figure 2c; Figure S10d, Supporting Information). This change could be ascribed to the redox driving force generated during the surface auto‐assembly involving O_2_ in air. By comparison to MoCC_2:4_ and MoCC_2:1_, MoCC_2:2_ (MoCC) exhibited significantly increased POD‐like activity, which could be attributed to the reason for the non‐single atomic state of MoCC_2:1_ and insufficient amount of Cu single atoms in MoCC_2:4_ (Figure S11, Supporting Information). As a result, MoCC was selected for further experiments.

The atomically dispersed Cu atoms were further confirmed uniformly dispersed on the MoS_2_ support by aberration‐corrected scanning transmission electron microscopy (AC‐STEM) (Figure 2d). In the AC‐STEM image, due to the high Z‐contrast sensitivity,^[^ ^51^ ^]^ elements with higher atomic numbers appear brighter compared to their lower atomic numbers counterparts. The low contrast of Cu atoms makes them more difficult to image compared with the high atomic number Mo atoms, and the extracted line intensity profiles (Figure 2e) along with the selected areas showed the atomically arrangement of Cu and Mo atoms, which validated the auto‐assembly of 3D Cys‐Cu‐Cys on MoS_2_. No cluster or nanoparticle was detected on the surface of MoCC, which was in accordance with the EDS mapping results. The structure model and free electron distribution were analyzed using electron localization functions (ELF), showing significant electron exchange and interaction between Cys‐Cu‐Cys and MoS_2_ in MoCC (Figure S12, Supporting Information).

Next, to gain insights into the atomic levels characteristics of Cu species within MoCC, synchrotron radiation (SR)‐based X‐ray absorption spectrum (XAS) was deployed. This technique, integrating X‐ray absorption near edge structure (XANES) and extended X‐ray absorption fine structure (EXAFS), facilitated a meticulous examination of coordination environment and chemical valence of Cu in MoCC. Normalized Cu *K*‐edge XANES profiles in Figure 2f disclosed that the absorption edge of Cu in MoCC exhibited a positive shift to high energy, closely resembling that of Cys‐Cu‐Cys and falling below that of CuO and CuS. This observation demonstrated that the average valance state Cu in MoCC ranged between Cu^0^ and Cu^2+^ with bonding sites of S‐Cu‐S, in accordance with what the XPS showed.

Cu *K*‐edge Fourier‐transformed (FT) ‐EXAFS curves of MoCC featured a characteristic peak at 1.78 Å, which was close to the *R* space curve of Cys‐Cu‐Cys (1.76 Å) and CuS (1.72 Å) with a slight shift, but different from CuO (1.56 Å) and Cu foil (2.24 Å) (Figure 2g). This confirmed that no Cu‐O or Cu–Cu coordination bonds existed. Through *R* space curve‐fitting (Figure 2h,i), K space EXAFS curve‐fitting (Figure S13, Supporting Information), and fitting parameters (Table S2, Supporting Information), it was revealed that the coordination structure of Cys‐Cu‐Cys and MoCC exhibited similar 3D S‐Cu‐S bonding configurations. In the case of Cys‐Cu‐Cys, the coordination number (CN) of Cu‐S was 2.0, with a Cu‐S bond distance of 2.25 ± 0.02 Å, indicating that a single Cu atom coordinated with two sulfur atoms that originated from the –SH groups of L‐Cys. For MoCC, the CN of Cu‐S was 1.8 with a bond distance of 2.23 ± 0.02 Å, closely aligning with the Cu‐S bond distance in the DFT simulated result of Cys‐Cu‐Cys in MoCC (2.12 Å) rather than S of MoS_2_ (Figure S14, Supporting Information). Since the CN of Mo in defect‐rich MoS_2_ was approximately 5.0, the CN of 1.8 for Cu clearly indicated that Mo atoms were not chemically replaced by Cu atoms.^[^ ^52^ ^]^ This finding further supported the notion that a weak interaction rather than chemical bonding existed between Cu single atoms and MoS_2_ support in MoCC. Furthermore, wavelet transforms (WT) analysis of Cu *K*‐edge EXAFS (Figure 2j–n) revealed that the peak intensity of the WT plots at 6.08 Å^−1^ and 6.12 Å^−1^ was respectively assigned to the S─Cu─S bond sites in Cys‐Cu‐Cys and MoCC, in comparison with Cu foil (Cu‐Cu, 7.87 Å^−1^), CuO (Cu‐O, 4.64 Å^−1^), and CuS (Cu‐S, 5.87 Å^−1^) references.^[^ ^24^ ^]^ In addition, Ellman's assay showed minimal free thiol content in MoCC (Figure S15, Supporting Information), confirming the effective S‐Cu‐S coordination. Therefore, the XAS analysis combined with XPS, Zeta potential, and DFT results confirmed that the Cu single atoms with 3D configuration S‐Cu‐S sites coordinated by L‐Cys were atomically dispersed to MoS_2_ support by electrostatic force to form MoCC SAzymes.

### 2.2 Enhanced POD‐Like Activity, CAT‐Like Activity, and Sono‐Piezoelectricity

**Figure 3:**
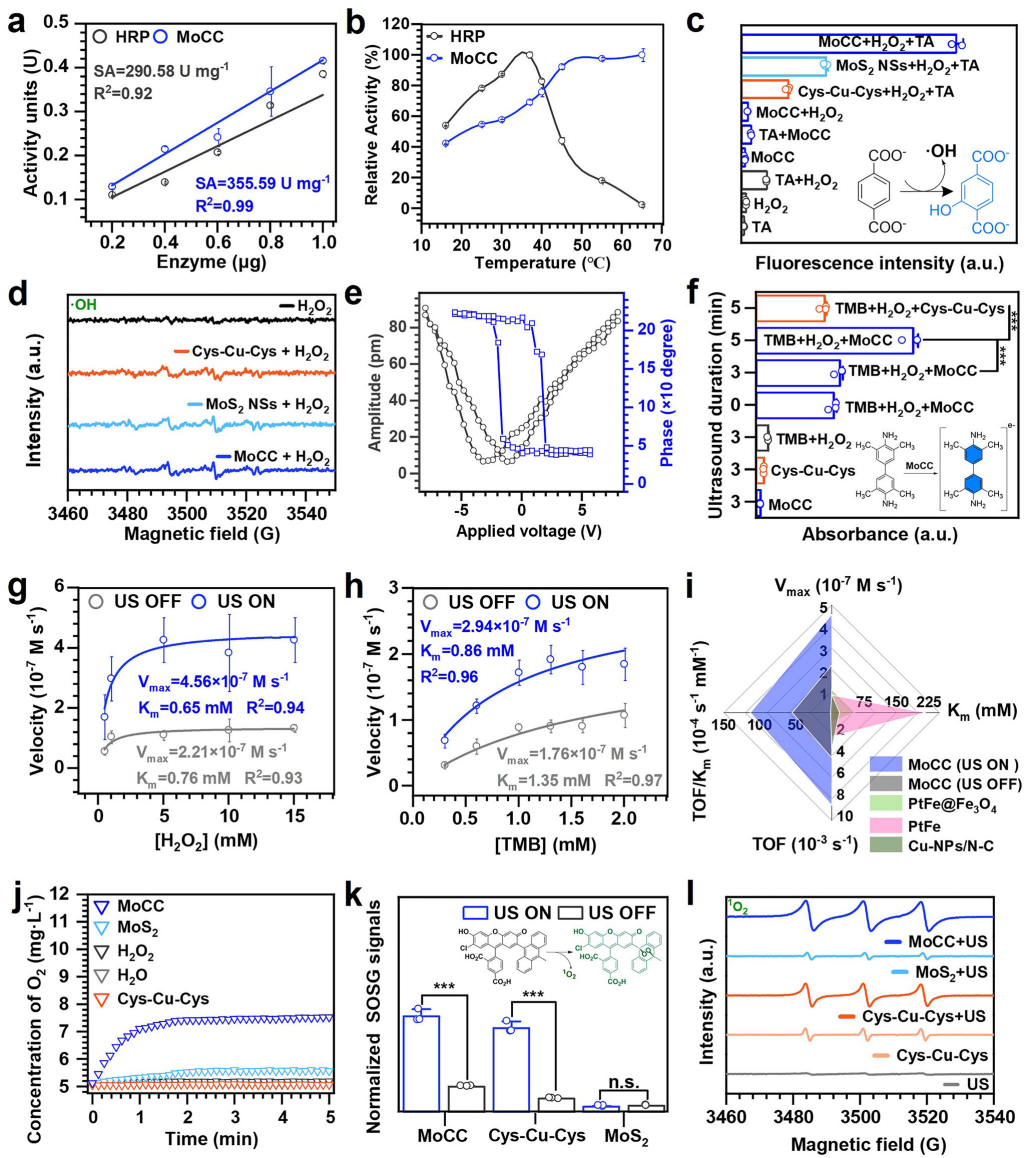
Enzyme‐like activity of MoCC SAzymes and US‐amplified catalysis performance. a) Comparison of SA (U mg^−1^) of MoCC and HRP measured in a 1 mL reaction system at pH 4.7, 37 °C. b) Comparison of POD‐like activity of HRP with MoCC at different temperatures. c) TAOH fluorescence intensity under different reaction conditions. d) ESR spectra of •OH. e) PFM characterization of MoCC. f) Absorbance at 652 nm after US irradiation of different groups. Catalytic kinetics of g) H_2_O_2_ and h) TMB with/without US irradiation of MoCC. i) Comparison of POD‐like catalytic parameters of MoCC with typical SAzymes. j) CAT‐like activities of MoCC for O_2_ generation at acidic oral microenvironment. k) Normalized fluorescence intensity of different groups for ^1^O_2_ detection using SOSG probe. *** indicates *p* < 0.001. l) Comparison of ESR spectra of ^1^O_2_ detection of MoCC, MoS_2_, and Cys‐Cu‐Cys under US irradiation.

The overexpressed H_2_O_2_ in acidic tooth biofilm could be decomposed into •OH through POD‐like activity of nanozyme.^[^ ^53^ ^]^ SA values (U mg^−1^) of MoCC as compared to natural HRP were assessed using 3,3′,5,5′‐tetramethylbenzidine (TMB) colorimetric method in the presence of H_2_O_2_ at an acidic microenvironment (**Figure** **3**a). MoCC exhibited superior POD‐like activity with SA value of 355.59 U mg^−1^. Their activity units (U) were calculated from the initial linear segments of catalytic reaction curve (Figure S16, Supporting Information). Moreover, the POD‐like activity of MoCC could experience an improvement with the elevation of temperature compared with HRP at 65 °C (Figure 3b; Figure S17, Supporting Information). The effective catalytic activity of MoCC was also comparable to HRP with a wide pH range (2.4–8.2) (Figure S18, Supporting Information). However, the activity of HRP was drastically reduced at the same storage conditions as MoCC (Figure S19, Supporting Information). These results indicated that MoCC possessed enhanced resilience to various harsh conditions. In addition, H_2_O_2_ concentration‐dependent POD‐like activity of MoCC indicated that optimal working concentration ranges of H_2_O_2_ were 10–20 mM (Figure S20a, Supporting Information). The catalytic velocity remained constant when MoCC was in the range of 0–100 µg mL^−1^ (Figure S20b, Supporting Information). Compared to MoS_2_ + H_2_O_2_ group, the absorbance at 652 nm in the MoCC + H_2_O_2_ group was found to increase by 3.47‐fold, indicating the superior POD‐like activity of MoCC activated by L‐Cys stabilized Cu single atoms (Figure S21a, Supporting Information). Following co‐incubation with terephthalic acid (TA), the fluorescence intensity in the MoCC + H_2_O_2_ group increased by 2.54‐fold compared with the MoS_2_ + H_2_O_2_ group (Figure 3c; Figure S21b, Supporting Information). These results confirmed that the POD‐like activity of MoCC was enhanced due to the maximized loading of Cu single‐atom. The formation of •OH was further confirmed by electron spin resonance (ESR) spectrum, where 5,5‐dimethyl‐1‐pyrroline N‐oxide (DMPO) served as the trapping agent (Figure 3d). MoCC + H_2_O_2_ exhibited the strongest signal of ·OH (1:2:2:1) than those of Cys‐Cu‐Cys + H_2_O_2_ and MoS_2_ + H_2_O_2_ systems.

It has been reported that MoS_2_ NSs exhibit a piezoelectric effect under US, leading to charge redistribution and enhanced generation of free radicals.^[^ ^54^ ^]^ Importantly, as previously reported, Cys‐Cu‐Cys can also function as a sonosensitizer to generate various free radicals.^[^ ^55^ ^]^ Therefore, we set out to investigate whether the Cu single‐atom‐loaded MoCC exhibit enhanced piezoelectric properties. Piezoresponse force microscopy (PFM) revealed a hysteresis amplitude‐voltage curve with a butterfly‐shape loop, suggesting a continuously varied strain under the application of an alternating current voltage to MoCC (Figure 3e). An average phase contrast of ≈180° in the hysteresis loop further confirmed the piezoelectricity of MoCC. Under US, this piezoelectricity enabled MoCC to generate positive and negative charges on its surface, consequently enhancing the POD‐like activity. Thereafter, TMB was used to measure the generation of ·OH under US (Figure 3f, Figure S22, Supporting Information). As expected, although the Cys‐Cu‐Cys + H_2_O_2_ + 3 min US and MoCC + H_2_O_2_ groups showed remarkable increase at 652 nm after incubation with TMB, MoCC + H_2_O_2_ + 5 min US group demonstrated the greatest level of absorbance in comparison to the other groups. This is because the 5‐min US enabled cumulative piezoelectric polarization of MoS_2_ to reach a critical threshold, synergizing with sonosensitive activation of Cys–Cu–Cys for much stronger cascaded ROS generation. Consequently, the US‐mediated catalytic amplification effect of MoCC arises from sonopiezoelectric polarization induced notable electron‐transfer effect. In addition, post‐catalysis XRD and morphology characterization demonstrated that MoCC maintained its crystal structure, as evidenced by unchanged diffraction peak positions (Figure S23a, Supporting Information) and preserved morphology (Figure S23b,c, Supporting Information), confirming its structural robustness under the short‐term catalytic conditions.

Steady‐state kinetic studies of MoCC exhibited a high maximum reaction velocity (4.56×10^−7^ M s^−1^) and a high affinity to the H_2_O_2_ substrate (K_m_, 0.65 mM) under US treatment, about 5.0 times and 4.5 times more superior than MoS_2_ (V_max_ = 0.91×10^−7^ M s^−1^, K_m_ = 2.92 mM) (Figure 3g; Figure S24a,c,e, Supporting Information), 16.3 times and 17.9 times superior than HRP (V_max_ = 0.28×10^−7^ M s^−1^, K_m_ = 11.63 mM, Table S3, Supporting Information) during the POD‐mimetic reactions. Similarly, kinetic studies with TMB as the substrate clearly confirmed the enhanced catalytic activity of MoCC (Figure 3h; Figure S24f, Supporting Information) over MoS_2_ (Figure S24b,d, Supporting Information). The significant increase in V_max_ and decrease in K_m_ strongly suggested that the 3D engineered electronic pocket of Cys‐Cu‐Cys effectively accelerated electron transfer, thereby substantially boosting catalytic efficiency and substrate affinity of MoCC. In addition, under US, V_max_ of MoS_2_ increased 2.12‐fold and 1.56‐fold for H_2_O_2_ and TMB, respectively (Figure S24a–d, Supporting Information), confirming piezoelectric polarization induced electron‐transfer. MoCC also demonstrated robust turnover frequency (TOF) and TOF/K_m_ values in the absence or presence of US using H_2_O_2_ as substrate, exceeding the reported typical single‐atom nanozymes and Cu single‐atom nanozymes synthesized via different strategies, further confirming its enhanced POD‐like activity and US‐amplified catalytic efficiency (Figure 3i, Tables S3–S5, Supporting Information). Nanozymes possessing CAT‐like activity are capable of decomposing H_2_O_2_ into O_2_ within tooth biofilm microenvironment to effectively kill anaerobic bacteria.^[^ ^56^ ^]^ Interestingly, we found that MoCC led to an increased generation of O_2_ bubbles in both acidic and neutral PBS solutions (Figure 3j; Figure S25a, Supporting Information). However, MoS_2_ and Cys‐Cu‐Cys presented negligible CAT‐like activity. This result indicated that MoCC also regulated catalytic performance to make CAT‐like activity emerge. Moreover, MoCC (MoCC_2:2_) had the highest O_2_ production, outperforming the MoCC_2:1_ and MoCC_2:4_, suggesting the optimum Cu single atoms loading ratio triggered the enhanced CAT‐like activity (Figure S25b, Supporting Information). Then, we plotted curves of O_2_ generation versus MoCC concentrations to derive catalytic kinetics parameters (Figure S26, Supporting Information). The V_max_ and K_m_ were determined to be 7.88×10^−7^ M s^−1^ and 3.72 mM, respectively, indicating that MoCC exhibited good affinity and fast reaction velocity for H_2_O_2_.^[^ ^57^ ^]^

Next, singlet oxygen sensor green (SOSG), which serves as a fluorescent probe for detection of ^1^O_2_, was used to measure the generation of ^1^O_2_ under US irradiation (Figure 3k; Figure S27, Supporting Information). The Cys‐Cu‐Cys + US group generated stronger fluorescence signals than MoS_2_ group in acidic PBS, further confirming the main role of Cys‐Cu‐Cys as sonosensitizer. Importantly, MoCC group presented the strongest fluorescence signal compared with other groups. Prolonged ultrasound time also resulted in increased ^1^O_2_ production of MoCC (Figure S28, Supporting Information). ESR spectra using TEMP‐^1^O_2_ trapping agent also revealed the highest sono‐piezoelectric polarization effect of MoCC among Cys‐Cu‐Cys and MoS_2_ systems in acidic PBS (Figure 3l), indicating the effective production of ^1^O_2_, which was attributed to the charge redistribution of MoCC to accelerate the electron transfer. Both the Cu single‐atom loading and US enhanced production of ·OH and ^1^O_2_ storm by MoCC, effectively targeting acidic oral biofilm microenvironment and inducing oxidative damage to eliminate bacteria and prevent dental caries.

### 2.3 DFT Calculation

**Figure 4:**
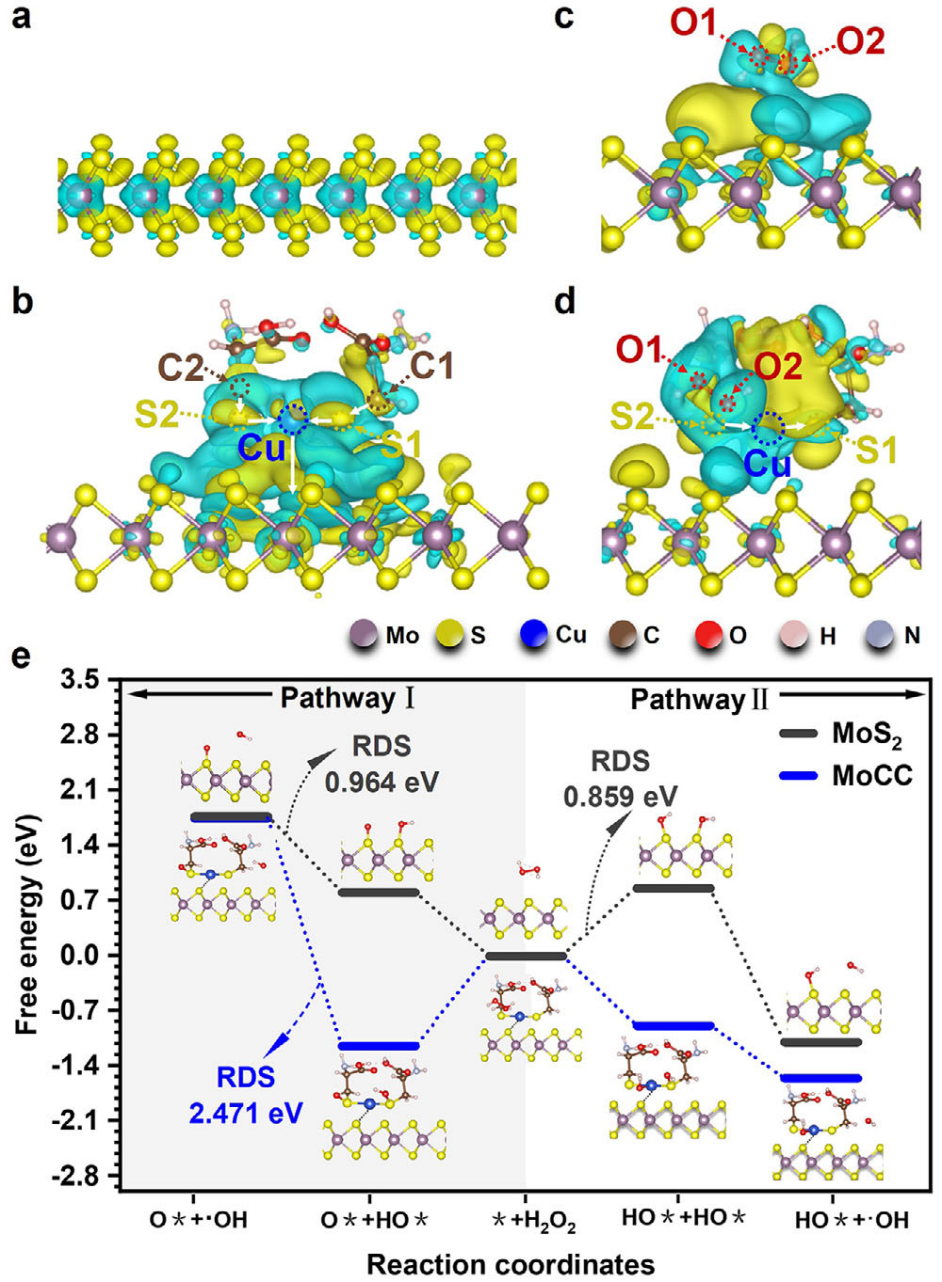
DFT analysis of the enhanced POD‐like catalytic mechanism of MoCC SAzymes. Charge density difference of a) MoS_2_ and b) MoCC. The optimized charge density difference for H_2_O_2_ absorbed on c) MoS_2_ and d) MoCC. The isosurface value was set to 0.0001 e Å^−3^. The yellow and cyan colors stood for the accumulation and depletion of electrons, respectively. The white arrows indicated electron flow directions. Atomic color coding in structure: Mo, dark purple; S, yellow; Cu, blue; C, brown; O, red; H, pink; and N, gray. e) Comparison of Gibbs free energy profiles of MoS_2_ and MoCC to produce •OH through Pathway I and Pathway II. * was denoted as the catalyst activity sites. Inserts were the corresponding atomic structure of intermediates during the absorption and decomposition of H_2_O_2_ and the production of •OH on MoS_2_ and MoCC.

The atomically dispersed Cu single atom chelated by two L‐Cys molecules auto‐assembled into a stable S‐Cu‐S active center on the MoS_2_ support to form MoCC SAzymes. The superior POD‐like catalytic performance of MoCC motivated us to comprehend the relationship between atomic structure and catalytic mechanism using DFT calculations. Differential charge density patterns (**Figure** **4**a–d) and Bader charge analysis (Table S6, Supporting Information) were conducted. MoS_2_ displayed electron flow from Mo to neighboring S atoms (Figure 4a). In contrast, the Bader charge analysis of MoCC (Table S6, Supporting Information) indicated that electrons flowed through the auto‐assembled Cu single atoms of 3D S‐Cu‐S catalytic pocket to MoS_2_ and were stored there (Figure 4b). In addition, the weak negative charge exchange (−0.017 e^−^) between Cu single‐atom and MoS_2_ in MoCC (Table S6, Supporting Information) further provided evidence that Cu single atoms were loaded onto MoS_2_ by electrostatic auto‐assembly rather than chemical bonding (Figure S29, Supporting Information).

After performing the POD‐like catalysis, it could be observed from the differential charge density analysis that the H_2_O_2_ substrate was surrounded by yellow and cyan electron clouds in MoS_2_ (Figure 4c), however, cyan was predominantly observed surrounding H_2_O_2_ in MoCC (Figure 4d). This indicated that there was a large difference between the interactions of MoS_2_ and H_2_O_2_ and those of MoCC and H_2_O_2_, which was further confirmed by H_2_O_2_^*^ adsorption energy (Figure S30, Supporting Information). The Bader charge (Tables S7, and S8, Supporting Information) also demonstrated a stronger binding ability of H_2_O_2_ on MoCC than MoS_2_. Importantly, in the S‐Cu‐S structure of MoCC, one of the original electron‐contributing sulfur atoms (S2) continued to lose electrons (‐ 0.044 e^−^), while another sulfur (S1) became an electron acceptor (+ 0.032 e^−^) (Table S8, Supporting Information). Electrons stored in MoS_2_ were reversibly transferred through the L‐Cys‐stabilized electronic bridge upon H_2_O_2_ absorption. This reversible electron‐transfer behavior of MoCC facilitated it to act as a “current bank”, with the crucial S1 flexibly adjusting electron distribution to meet the demands of the oxidation reaction.

In the POD‐like catalysis, the O‐O bond in H_2_O_2_ breaks to form free radicals, summarized by two equations (Equations (S1) and (S2) in the supporting information). In MoS_2_ model, two free radicals generation pathways were proposed based on free energy diagrams. However, intermediates OH* + O* (+ 0.964 eV) in pathway I and 2OH* (+ 0.859 eV) in pathway II had positive free energy, suggesting unfavorable adsorption (Figure 4e), limiting catalytic efficiency. In contrast, an electron‐accepting sulfur 1 (S1) was provided for oxygen 1 (O1) from H_2_O_2_, while oxygen 2 (O2), which needed to accept electron, might adsorb to Cu or sulfur 2 (S2) from MoCC (Table S8, Supporting Information). This process led to the establishment of a dual‐path electron transfer mechanism, as illustrated in Figure 4e. In pathway I of MoCC, OH* and O* combined with electron‐gaining S2 and electron‐losing S1 (−1.144 eV), respectively. In contrast, in pathway II, intermediates OH* and OH* bound to Cu and electron‐gaining S2, respectively. It should be noted that, although MoCC could produce •OH in both pathway I and pathway II, the amount of •OH in pathway II was more than that in pathway II, as a high free energy (+2.471 eV) in the rate determining step (RDS) of pathway I hindered the production of •OH, slowing the reaction. The release of •OH from S2 in pathway II maintained a negative free energy (−0.67 eV), implying a spontaneous reaction. The 3D S‐Cu‐S structure mimicked catalytic pockets of natural HRP for better substrate binding. Besides, electrons originally stored in MoS_2_ were reversibly transferred through the L‐Cys‐stabilized electronic bridge. Furthermore, this adaptability allowed MoCC to adjust the electron distribution according to the demands of H_2_O_2_ decomposition, reducing the overall reaction energy barrier. This electron flexibility was crucial for stabilizing intermediates and thus made the Cu single atoms pivotal for a continuous electron flow, enabling significantly efficient H_2_O_2_ decomposition. In comparison, the planar structure of MoS_2_ alone, with high surface energy, was prone to layer stacking and reduced surface area, which could limit its catalytic activity. Consequently, L‐Cys stabilized Cu single‐atom active centers effectively mimic the 3D structure of natural HRP, which was more beneficial to improve the POD‐like catalytic performance.

### 2.4 Antibacterial Performance

**Figure 5:**
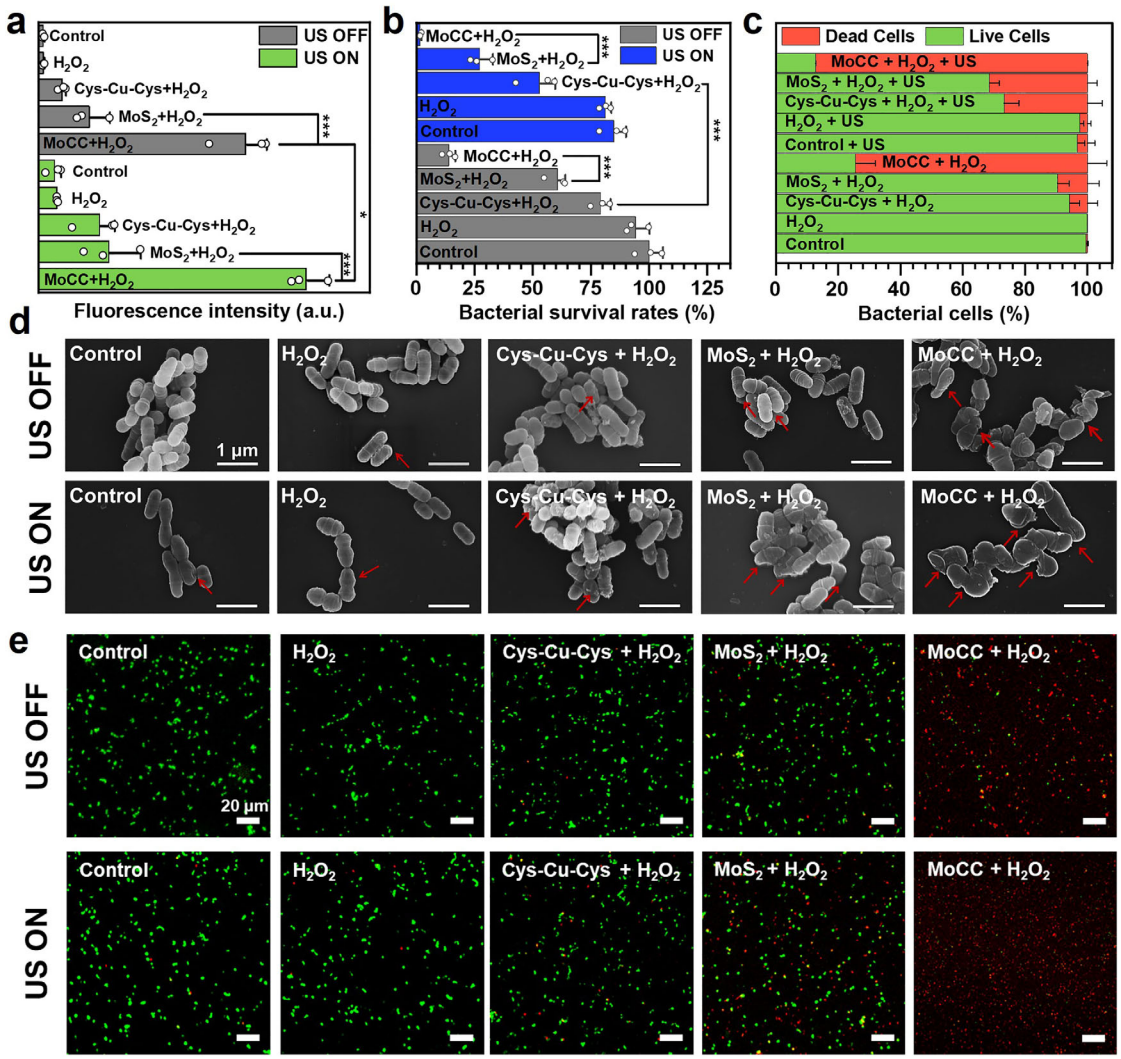
Bactericidal performance of MoCC in vitro. a) DCF fluorescence intensity after various treatments. b) Bacterial survival rates determined by plate counting method. c) Corresponding fluorescence intensity of e). d) SEM images of *S. mutans* after different treatments. (e) Confocal laser scanning microscope (CLSM) images of SYTO (green) and PI (red) stained *S. mutans* after various treatments. * and *** indicate *p* < 0.05 and *p* < 0.001, respectively.

*Streptococcus mutans* (*S. mutans*), a primary pathogen of dental caries, ferments sugars to produce lactic acid, leading to an acidic oral environment that promotes biofilm formation and induces caries.^[^ ^58^ ^]^ Inspired by the exceptional POD‐like activity and synergistic cascaded CAT‐like/sono‐piezocatalysis effect of MoCC triggered by US, which amplified O_2_ and ^1^O_2_ production, we further evaluated its bactericidal efficacy in vitro. First, to understand the potential antibacterial mechanism related to Cu ions, the pH‐responsive release of Cu ions from MoCC was evaluated. It was demonstrated that 12.3% of Cu ions were released from MoCC within 24 h (Figure S31a, Supporting Information), leading to a 20% bacterial cell death under acidic conditions mimicking oral microenvironment (Figure S31b,c, Supporting Information). This might be attributed to the damage inflicted by Cu ions.^[^ ^59^ ^]^ Next, for intracellular ROS level evaluation, 2′,7′‐dichlorodihydrofluorescein diacetate (DCFH‐DA), a typical ROS fluorescent probe, was employed. Both the control and H_2_O_2_ groups exhibited minimal green fluorescence (Figure S32, Supporting Information). In contrast, MoCC + H_2_O_2_ group displayed fluorescence intensities 8.48‐ and 2.54‐fold higher than the Cys‐Cu‐Cys + H_2_O_2_ and MoS_2_ + H_2_O_2_ groups, respectively (**Figure** **5**a; Figure S32, Supporting Information). Notably, MoCC + H_2_O_2_ + US group exhibited the most obvious increase of green fluorescence compared with other groups (Figure 5a). The fluorescence induced by ^1^O_2_ can be detected using SOSG probe, especially in MoCC + US and Cys‐Cu‐Cys + US groups, confirming the US‐induced ROS storm within bacterial cells (Figure S33, Supporting Information), further improving the antibacterial effect. The antibacterial performances were then assessed using plate counting method (Figure 5b; Figure S34, Supporting Information). In the Cys‐Cu‐Cys + H_2_O_2_ + US group, bacterial survival rate significantly decreased compared to Cys‐Cu‐Cys + H_2_O_2_ group, attributed to the US‐induced ^1^O_2_ generation. The MoS_2_ + H_2_O_2_ group demonstrated enhanced bacterial death due to the POD‐like activity elevated ROS level. Notably, MoCC + H_2_O_2_ + US group achieved the lowest bacterial survival rate of 1.41%, highlighting its superior antibacterial performance via synergistic effects of US‐amplified POD‐like cascaded ROS storm. In Figure 5d, SEM images revealed that, when compared with the control groups, the treatments of Cys‐Cu‐Cys + H_2_O_2_ and MoS_2_ + H_2_O_2_ groups caused slight morphological changes of bacterial cell walls as marked by red arrows. In contrast, MoCC + H_2_O_2_ and MoCC + H_2_O_2_ + US treatment caused extensive damage to bacterial cells, which appeared evidently flattened or fragmented. The survival rates of *S. mutants* were validated by live (green)/dead (red) staining. Predominantly green signals were observed in the control group and H_2_O_2_ group. However, after treatment with MoCC + H_2_O_2_, 74.39% red dead bacterial cells were observed, confirming the enhanced POD‐like activity induced bacterial apoptosis. Furthermore, the ratio of dead bacteria increased to 87.3% for the MoCC + H_2_O_2_ + US group compared to the Cys‐Cu‐Cys + H_2_O_2_ + US group (26.73%) and MoS_2_ + H_2_O_2_ + US (31.41%) group due to ROS storm induced by the POD‐like activity synergistic cascaded CAT‐like and sono‐piezocatalysis effect (Figure 5c,e).

### 2.5 Elimination of Oral Biofilms and Teeth Whitening In Vitro

**Figure 6:**
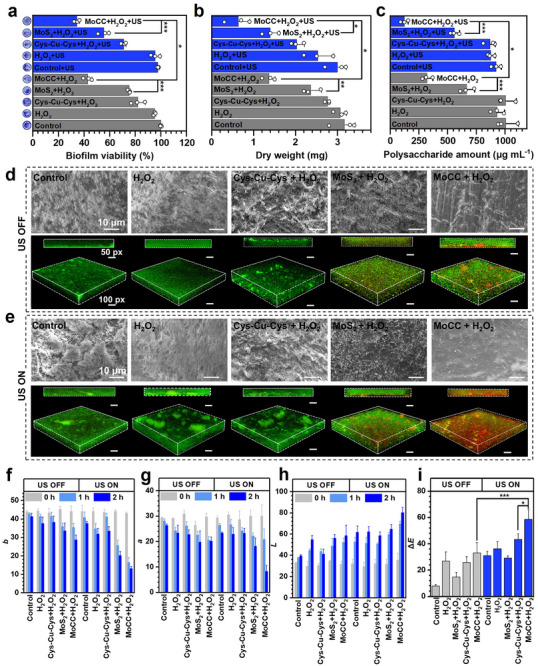
Performance of MoCC in biofilms elimination and teeth whitening. a) Biofilms viability after various treatments, inserted were images of crystal violet stained *S. mutans* biofilms. b) Dry weights and c) polysaccharides level of *S. mutans* biofilms after various treatments. d,e) SEM and corresponding CLSM (side and 3D views) images of *S. mutans* biofilms with and without US treatment. Comparison of various treatments on the teeth whitening levels characterized by CIE‐Lab system: color changes in f) red–green axis and g) blue‐yellow axis; h) lightness; i) color differences. *, **, and *** indicate *p* < 0.05, *p* < 0.01, and *p* < 0.001, respectively.

The strategy of POD‐like activity synergizing cascaded CAT‐like/sono‐piezocatalysis of MoCC was employed to evaluate the *S. mutans* biofilms elimination efficacy. Interestingly, MoCC + H_2_O_2_ readily adhered to the biofilms and induced a chromogenic reaction by oxidizing TMB within 10 min, suggesting its potential for rapid elimination of biofilms during the POD‐like catalytic reaction (Figure S35, Supporting Information). Crystal violet staining revealed that MoCC + H_2_O_2_ group achieved 56.97% bacterial biofilm inactivation, demonstrating great biofilm elimination efficiency compared with the MoS_2_ + H_2_O_2_ (24.8%) and Cys‐Cu‐Cys + H_2_O_2_ (18.05%) groups. After further US treatment, the inactivated biofilm in the MoCC + H_2_O_2_ + US group reached 65.53% (**Figure** **6**a). Consistently, the dry weights of treated biofilms reduced by 80.13% in the MoCC + H_2_O_2_ + US group (Figure 6b). Under US treatment, polysaccharides content in the MoCC + H_2_O_2_ + US group further reduced to 12.16% (Figure 6c). The obviously reduced green fluorescent signals of EPS labeled by FITC‐ConA revealed that EPS networks were rarely detected in the MoCC + H_2_O_2_ group and MoCC + H_2_O_2_ + US group, further suggesting the effective elimination of biofilms induced by the cascaded ROS storm (Figure S36, Supporting Information). SEM and CLSM were further used to evaluate the anti‐biofilm performance. Dense, intact biofilms were observed in control and H_2_O_2_ groups (Figure 6d,e). In the Cys‐Cu‐Cys group and Cys‐Cu‐Cys + US group, minor morphological changes of biofilms were detected, and the bacteria were mostly unharmed, as indicated by the predominance of green fluorescence, likely due to insufficient free radicals production. In MoS_2_ groups, although US‐enhanced POD‐like activity partially eliminated biofilms, embedded bacteria cells remained viable, as evidenced by faint red fluorescence. In contrast, the MoCC + H_2_O_2_ group demonstrated a significant reduction in biofilm density, and the addition of US led to the near‐complete eradication of embedded bacterial cells. This enhanced effect was attributable to the US‐amplified acidic biofilm‐responsive ROS storm, which effectively eliminated both biofilms and bacteria.

Since persistent biofilm is the main cause of tooth staining,^[^ ^60^ ^]^ we further evaluated the teeth‐whitening performance of MoCC‐triggered ROS storm. Rhodamine B (RhB) served as a staining agent. The MoCC + H_2_O_2_ group resulted in a significant 62.5% degradation of RhB. Upon US, only 13.9% of RhB remained (Figure S37a,b, Supporting Information), demonstrating the enhanced effectiveness of teeth‐whitening. Teeth whitening experiment was then conducted using teeth stained to mimic natural discoloration. At the beginning (0 h), stains were predominantly concentrated at the root of teeth (Figure S38, Supporting Information). After 2 h of treatment, obvious decolorization was observed in grooves area of the teeth in MoCC + H_2_O_2_ group, and the heavily stained root area was further brightened with US treatment compared with other groups. The Commission International De L'Eclairage (CIE‐lab) system was used to quantitatively evaluate teeth‐whitening effects. In MoCC + H_2_O_2_ group, the *a* and *b* values (representing staining intensity) decreased significantly, while the L value (indicating brightness) increased notably in MoCC + H_2_O_2_ + US group due to the US‐amplified ROS storm (Figure 6f–i). The ΔE value for the MoCC + H_2_O_2_ group was 1.6 times greater compared to that of the H_2_O_2_ group, and with the application of US, the color differences were further enhanced by 35%. These findings demonstrated that MoCC‐catalyzed sustained free radicals release amplified by US treatment strategy was simple, fast and easy to operate, which had great potential for clinical teeth‐whitening therapies.

### 2.6 In Vivo Prevention of Dental Caries of MoCC

Biocompatibility of nanomaterials is crucial for their bio‐applications. MoCC was found to exhibit negligible cytotoxicity at effective antibacterial concentrations (<50 µg mL^−1^), with survival rates of 95.1% for human umbilical vein endothelial cells (HUVECs) and 96.2% for human keratinocytes (HaCaT) after 24 h of incubation (Figure S39, Supporting Information). In addition, MoCC significantly enhanced normal cell migration (Figure S40, Supporting Information), suggesting its potential to promote periodontal cell proliferation.

**Figure 7:**
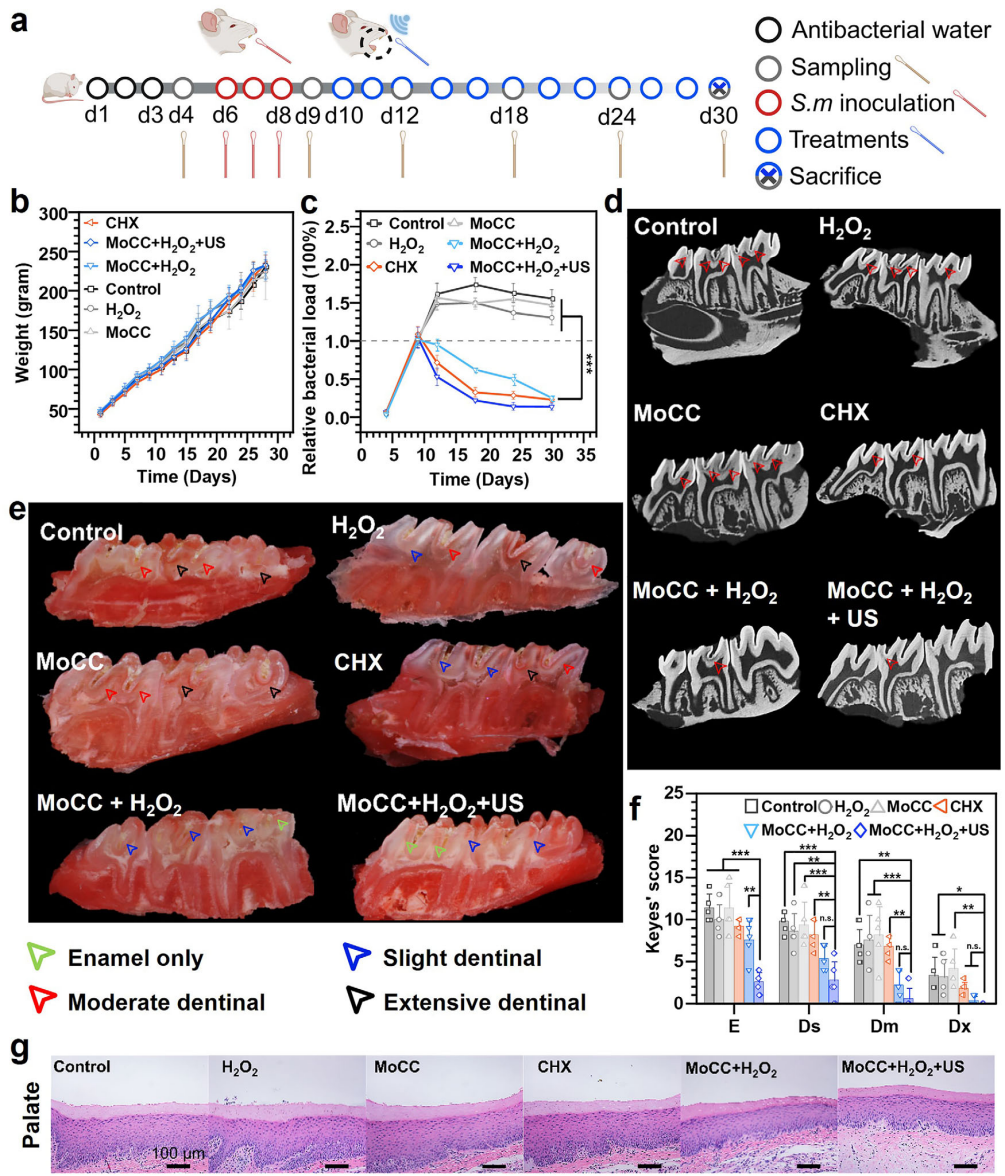
MoCC catalytically enhanced prevention of dental caries in vivo. a) Schematic illustration of the caries‐prevention model. b) Body weight records of SD rats. c) Relative bacterial loads on Days 4, 9, 12, 18, 24, 30. d) Micro‐CT images of molar teeth (red arrows indicated caries lesion sites). e) Images of molar lateral incision stained with murexide, illustrating different caries severities (green arrows: affected enamel only; blue arrows: slight dentinal involvement, within 1/4 of the dentin; red arrows: moderate dentinal involvement, 1/4 – 3/4 of the dentin; black arrows: extensive dentinal involvement, beyond 3/4 of the dentin). f) Kaye's Caries scores (E, enamel only; Ds, slight dentinal; Dm, Moderate dentinal; Dx, Extensive dentinal). g) Histological analysis of the palate tissue.*, **, and *** indicate *p* < 0.05, *p* < 0.01, and *p* < 0.001, respectively.

Next, we constructed a dental caries model using Sprague‐Dawley (SD) rats (3 weeks old, male) (**Figure** **7**a). After 3 consecutive days of infection, oral samples were collected on day 9 (d9), confirming the successful colonization of *S. mutans* (Figure 7c; Figure S41, Supporting Information). To ensure its effective retention on the teeth surface, MoCC was encapsulated in sodium alginate and cross‐linked with Ca^2+^ to form a hydrogel coating on the teeth crown (Figure S42, Supporting Information). After 20 days of treatment, all groups exhibited consistent weight gain, indicating no adverse effects on the overall health of SD rats (Figure 7b). To compare the therapeutic effect of MoCC‐catalyzed ROS storm with clinical anti‐caries therapy, chlorhexidine (CHX), a broad‐spectrum antibacterial agent that inhibits the growth of cariogenic bacteria,^[^ ^61^ ^]^ was employed as a positive group. Compared with the control, MoCC, and H_2_O_2_ groups, the oral bacterial loads significantly decreased in the CHX, MoCC + H_2_O_2_, and MoCC + H_2_O_2_ + US groups (Figure 7c). On day 18 (d18), the oral bacterial loads in control, MoCC, and H_2_O_2_ groups increased by 74%, 50%, and 59%, respectively, compared to d9. However, the bacterial loads in MoCC + H_2_O_2_, CHX, and MoCC + H_2_O_2_ + US groups decreased to 25.3%, 22.7%, and13.6%, respectively, by day 30 (d30), demonstrating the outstanding caries prevention effect of MoCC SAzymes. The main organs of the SD rats were collected on the final day. Molars from the control, H_2_O_2_, and MoCC groups displayed dark caries lesions (Figure S43, Supporting Information), confirming the successful model establishment. Caries severity was obviously reduced in the MoCC + H_2_O_2_ and CHX groups, with minimal caries in the MoCC + H_2_O_2_ + US group, demonstrating the effective inhibition of cariogenic bacteria. Micro‐computed tomography (Micro‐CT) imaging revealed reduced enamel density in caries lesions, with the MoCC + H_2_O_2_ and MoCC + H_2_O_2_ + US groups exhibiting minimal radiolucent lesions (red arrows) (Figure 7d). Murexide staining was further applied to quantify caries severity in these groups. As shown in Figure 7e, the dye penetrated carious enamel, resulting in varying staining levels, whereas intact enamel remained mostly unaffected. Blinded Kaye's scoring revealed minor differences in caries severity among the control, MoCC, and H_2_O_2_ groups, while the MoCC + H_2_O_2_ and MoCC + H_2_O_2_ + US groups showed significantly lower scores, with caries limited to enamel and superficial dentin (Figure 7f). Hematological parameter analysis showed no abnormalities after different treatments (Figure S44, Supporting Information), confirming the safety of MoCC to the liver and spleen functions. The hemolysis rate at all tested concentrations remained below 4%, indicating good blood compatibility of MoCC (Figure S45, Supporting Information). Hematoxylin and eosin (H&E) staining showed no tissue damage, inflammation, or MoCC accumulation at the palates, tongues, or major organs (Figure 7g; Figure S46, Supporting Information), further demonstrating the potential of MoCC SAzymes for anti‐caries applications.

### 2.7 Changes in the Oral Microbial Communities by MoCC Catalysis‐Triggered Caries Prevention

**Figure 8:**
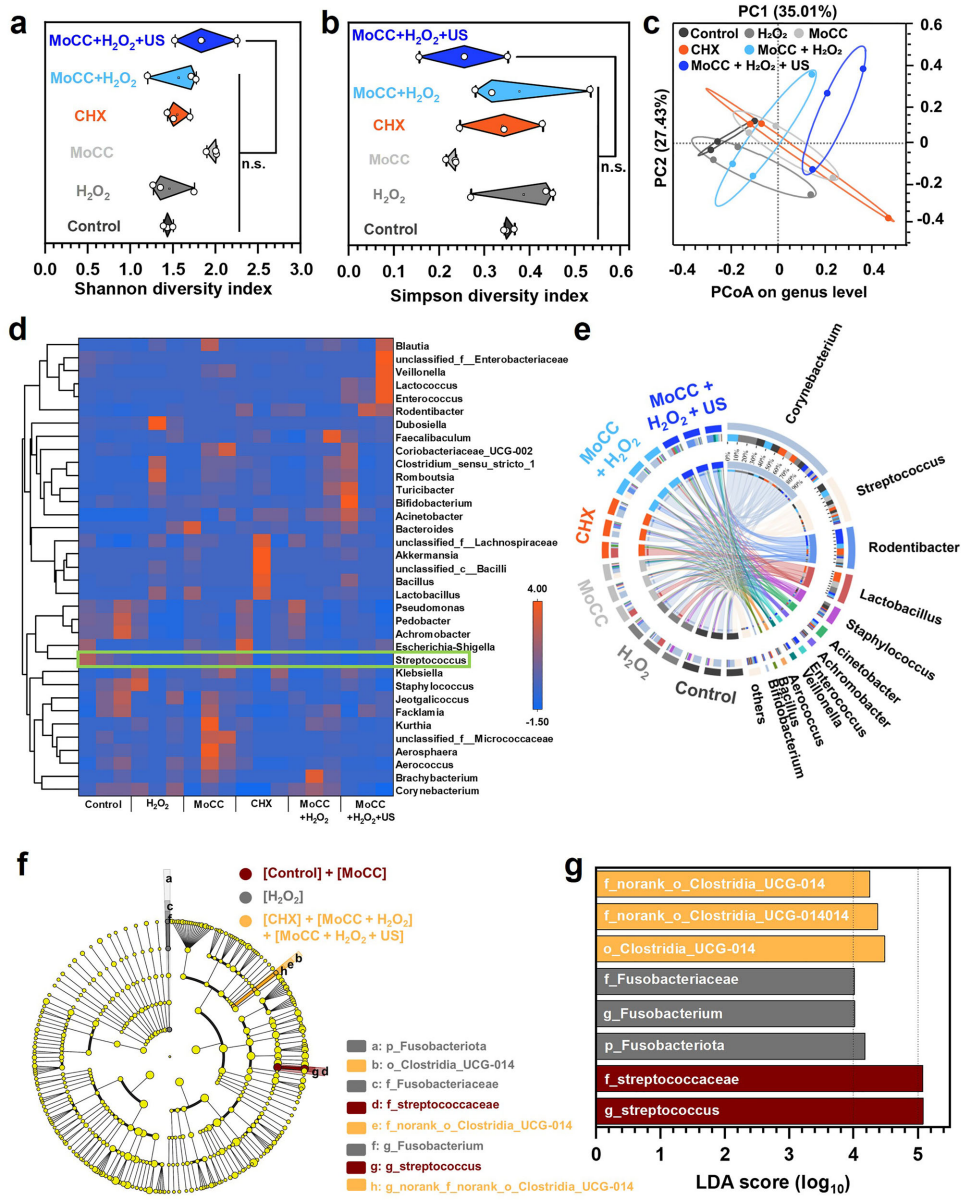
Oral microbiota analysis through 16s ribosomal RNA (16S rRNA). The α‐diversity of Oral microbiome illustrated by a) Shannon index and b) Simpson index. c) β‐diversity of oral microbiome demonstrated by PCoA at the genus‐level. d) Heat map for visualizing the relative abundance of taxon (rows) at the genus level between various groups. e) Circular plot description. f) Cladogram based on LEfse analysis revealing the community composition of the oral microbiota in rats with various treatments. Yellow circles depicted the taxa presented but not enriched. Wine‐red, grey, and orange circles were enriched in the [control] + [MoCC], [H_2_O_2_], and [CHX] + [MoCC + H_2_O_2_] + [MoCC + H_2_O_2_ + US] groups, respectively. g) Taxa listed according to their LDA values determined from comparisons between the [control] + [MoCC], [H_2_O_2_], and [CHX] + [MoCC + H_2_O_2_] + [MoCC + H_2_O_2_ + US] using the LEfSe method. The LDA cut‐off values were set at 4.0. Data were presented as mean ± s.d. from *n* = 3 biological replicates.

The human oral cavity harbors a diverse microbial community, in which symbiotic bacteria are crucial for maintaining oral health.^[^ ^62^ ^]^ While eliminating pathogenic microorganisms is essential, preserving the stability of symbiotic microbiota is equally important. Disruptions in this balance might lead to halitosis, periodontal disease, and even endodontic infections. Therefore, 16s rRNA sequencing was performed to analyze microbial diversity and composition in the oral cavity of SD rats after MoCC SAzymes triggered ROS‐mediated caries prevention. Alpha diversity, evaluated using Shannon and Simpson indices, revealed no significant differences among the groups (**Figure** **8**a,b), suggesting that microbial richness and evenness remained consistent. Principal Coordinates Analysis (PCoA) demonstrated that the microbial composition of the H_2_O_2_, MoCC, and CHX groups exhibited similarities to that of the control group, forming overlapping clusters. In contrast, the microbial components of the MoCC + H_2_O_2_ and MoCC + H_2_O_2_ + US groups exhibited minimal similarity to the control group, indicating significant differences in certain microbial components (Figure 8c). The genus‐level heatmap further confirmed the significantly reduced abundance of *Streptococcus* in the MoCC + H_2_O_2_ and MoCC + H_2_O_2_ + US groups, consistent with previous animal experiments, highlighting the superior antibacterial effects of ROS storm induced by MoCC (Figure 8d). The Circos plot also confirmed the reduction of *Streptococcus* in the MoCC + H_2_O_2_ and MoCC + H_2_O_2_ + US groups (Figure 8e), while *Corynebacterium* remained predominant among all the groups, contributing to oral health by aiding digestion and preventing pathogen colonization. This demonstrated that MoCC can effectively inhibit pathogenic colonization with minimal impact on native microbes, underscoring its safety for oral use. The six experimental groups in the above animal antibacterial study were then classified as untreated ([control] + [MoCC]), moderately effective ([H_2_O_2_]), and significantly effective groups ([CHX] + [MoCC + H_2_O_2_] + [MoCC + H_2_O_2_ + US]). LEfSe and Linear Discriminant Analysis (LDA) (Figure 8f,g) identified *Streptococcus* as a key marker distinguishing the significantly effective group, thereby confirming the strong antibacterial effects of MoCC + H_2_O_2_ and MoCC + H_2_O_2_+ US groups. These findings highlighted that MoCC had effective pathogen‐clearing ability while preserving beneficial bacteria, suggesting it as a promising candidate for dental caries prevention.

## 3 Conclusion

This study reports biomimetic Cu single‐atom MoCC SAzymes, which possess 3D S‐Cu‐S catalytic sites and are formed through the L‐Cys‐triggered auto‐assembly process, achieving a high Cu single‐atom loading ratio of 10.11%. The optimized MoCC demonstrates exceptional POD‐like activity, with a V_max_ and affinity that are 16.3‐ and 17.9‐fold, respectively, higher than those of natural HRP. DFT calculation reveals that the S and Cu atoms in 3D ‐S‐Cu‐S‐ catalytic pocket work in concert as an electron flow workstation for electron storage and transfer, with the S atoms experiencing reversible electron transfer and the Cu atom donating electrons to MoS_2_ for store. During the POD‐like process, the electrons stored in MoS_2_ flow reversibly to 3D catalytic pocket, enabling rapid electron exchange with H_2_O_2_ and reducing the reaction energy barrier. Compared with the MoS_2_ alone, which lacks the 3D‐biomimetic configuration, MoCC binds H_2_O_2_ more efficiently, lowers the reaction energy barrier and boosts H_2_O_2_ decomposition efficiency, offering a distinctive electronic regulation mode for efficient POD‐like catalysis. Compared to previously reported MoS_2_‐supported SAzymes, including electrostatically adsorbed Cu–MoS_2_ and multi‐step leached Co–MoS_2_ systems, MoCC features a one‐step L‐Cys–guided assembly with high Cu single‐atom loading, and superior POD‐like catalytic kinetic performance. Compared with conventional Cu‐based nanozymes, MoCC uniquely combines mild, room‐temperature preparation with atomically dispersed and well‐defined active centers, thereby offering both structural precision and practical scalability. Although L‐Cys can coordinate with a variety of transition metals, including Fe, Co, Ni, and Cd, via thiol, amine, and carboxyl groups,^[^ ^63^ ^]^ Cu‐L‐Cys complex exhibits its distinctive advantages: (i) a strong and stable affinity for sulfur ligands, enabling robust S–Cu–S active site formation; (ii) favorable redox flexibility (Cu^+^/Cu^2+^), which promotes efficient electron transfer and H_2_O_2_ activation; (iii) good biocompatibility and low toxicity, which are particularly important for biomedical applications; and (iv) low cost and synthetic scalability compared to noble metals.^[^ ^24^, ^43^ ^]^ Furthermore, MoCC exhibits cascaded CAT‐like and sono‐piezocatalytic activities under US stimulation, synergistically producing •OH, O_2_, and ^1^O_2_. This multi‐radical system demonstrates potent antibacterial efficacy against *S. mutans*, achieving 98.59% bacterial eradication in vitro and significantly reducing dental caries severity in vivo. The acidic oral microenvironment‐responsive ROS storm effectively degrades biofilms and restores tooth whiteness by degrading organic stains. Importantly, MoCC maintains good biocompatibility and stability under physiological conditions, with minimal impact on symbiotic oral microbiota, supporting its potential translation into oral healthcare formats such as coatings, rinses, or US‐triggered dental patches. This work establishes a biomimetic, non‐invasive catalytic strategy for activating nanozymes into 3D L‐Cys stabilized Cu SAzymes for promoting the POD‐mimicking activity surpassing natural HRP. The L‐Cys‐triggered auto‐assembly and reversible electron transfer mechanism provide valuable insights for designing high‐performance nanozymes.

## Conflict of Interest

The authors declare no conflict of interest.

## Supporting information



Supporting Information

## Data Availability

The data that support the findings of this study are available from the corresponding author upon reasonable request.
