# Supplementary material for: Engineering Dimensional Configuration of Single‐Atom S‐Cu‐S Sites as Reversible Electron Station for Enhanced Peroxidase‐Mimicking
Source: Adv Sci (Weinh). 2025 Oct 3;13(7):e10133. doi: 10.1002/advs.202510133 (PMC12866787; doi:10.1002/advs.202510133)
Supplement: Supplementary file 1 — Supporting Information [file ADVS-13-e10133-s001.docx]

***Supporting Information***

**Engineering Dimensional Configuration of Single-Atom S-Cu-S Sites as Reversible Electron Station for Enhanced Peroxidase-Mimicking**

Wenjie Ma,^1, 2^ Qian He,^3^ Jiancheng Sun,^3^ Yiqing Chen,^1, 6^ Hongfei Su,^1^ Ludan Zhang,^5^ Xiao He,^1^ Yuguang Wang,^5^ Changjian Xie,^6^ Zhiyong Zhang,^1, 4^ Xin Zhou,^2,^ * Yuliang Zhao,^1^ and Wenyan Yin^1, 4,^ *

^1^CAS Key Laboratory for Biomedical Effects of Nanomaterials and Nanosafety & CAS Center for Excellent in Nanoscience, Institute of High Energy Physics and National Center for Nanoscience and Technology of China, Chinese Academy of Sciences, Beijing 100049, China

^2^College of Veterinary Medicine, Institute of Comparative Medicine, Yangzhou University, Yangzhou 225009, China

^3^School of Light Industry and Food Engineering, Guangxi University, Nanning 530004, China

^4^Jinan Laboratory of Applied Nuclear Science, Jinan 250131, China

^5^Center of Digital Dentistry, Peking University School and Hospital of Stomatology & National Center of Stomatology, Beijing 100081, China

^6^School of life Sciences and medicine, Shandong University of Technology, Zibo 255000, Shandong, China

*Corresponding authors.

Email: [zhou_xin@126.com](mailto:zhou_xin@126.com) (X. Z.), [yinwy@ihep.ac.cn](mailto:yinwy@ihep.ac.cn) (W. Y.)

**Table of Contents**

[**Supporting Methods** 4](#_Toc210060765)

[Chemicals 4](#_Toc210060766)

[Preparation of MoS_2_ NSs 4](#_Toc210060767)

[Synthesis of MoCC SAzymes 4](#_Toc210060768)

[Characterizations 5](#_Toc210060769)

[Chemical speciation analysis 5](#_Toc210060770)

[Ellman’s assay 5](#_Toc210060771)

[Cu single-atom enhanced POD-like activity and US-amplified catalytic activity 6](#_Toc210060772)

[Catalase-like activity measurement 7](#_Toc210060773)

[Detection of sono-piezocatalysis triggered ^1^O_2_ 8](#_Toc210060774)

[S. mutans culture 8](#_Toc210060775)

[In vitro bacterial killing assay 9](#_Toc210060776)

[Bacterial morphology 9](#_Toc210060777)

[SYTO/PI staining 9](#_Toc210060778)

[ROS detection 10](#_Toc210060779)

[Anti-biofilm efficiency in vitro 10](#_Toc210060780)

[RhB degradation assay 11](#_Toc210060781)

[Biofilms morphology 11](#_Toc210060782)

[Teeth whitening experiments in vitro 11](#_Toc210060783)

[In vitro cytotoxicity 12](#_Toc210060784)

[Cell migration test 12](#_Toc210060785)

[In vivo anti-caries model 12](#_Toc210060786)

[In vivo dental caries treatments 13](#_Toc210060787)

[Anti-caries efficiency in animal models 13](#_Toc210060788)

[Micro-architecture and carious levels after MoCC treatments 13](#_Toc210060789)

[Hemolysis assay 14](#_Toc210060790)

[Computational methods 14](#_Toc210060791)

[Statistical analysis and graphics 14](#_Toc210060792)

[**Supporting Tables** 15](#_Toc210060793)

[Supporting Table S1. 15](#_Toc210060794)

[Supporting Table S2. 16](#_Toc210060795)

[Supporting Table S3. 17](#_Toc210060796)

[Supporting Table S4. 18](#_Toc210060797)

[Supporting Table S5. 19](#_Toc210060798)

[Supporting Table S6. 20](#_Toc210060799)

[Supporting Table S7. 21](#_Toc210060800)

[Supporting Table S8. 22](#_Toc210060801)

[**Supporting Figures** 23](#_Toc210060802)

[Figure S1. 23](#_Toc210060803)

[Figure S2. 23](#_Toc210060804)

[Figure S3. 24](#_Toc210060805)

[Figure S4. 24](#_Toc210060806)

[Figure S5. 24](#_Toc210060807)

[Figure S6. 25](#_Toc210060808)

[Figure S7. 25](#_Toc210060809)

[Figure S8. 26](#_Toc210060810)

[Figure S9. 26](#_Toc210060811)

[Figure S10. 27](#_Toc210060812)

[Figure S11. 28](#_Toc210060813)

[Figure S12. 28](#_Toc210060814)

[Figure S13. 28](#_Toc210060815)

[Figure S14. 29](#_Toc210060816)

[Figure S15. 29](#_Toc210060817)

[Figure S16. 30](#_Toc210060818)

[Figure S17. 30](#_Toc210060819)

[Figure S18. 30](#_Toc210060820)

[Figure S19. 31](#_Toc210060821)

[Figure S20. 31](#_Toc210060822)

[Figure S21. 32](#_Toc210060823)

[Figure S22. 32](#_Toc210060824)

[Figure S23. 32](#_Toc210060825)

[Figure S24. 33](#_Toc210060826)

[Figure S25. 34](#_Toc210060827)

[Figure S26. 34](#_Toc210060828)

[Figure S27. 35](#_Toc210060829)

[Figure S28. 35](#_Toc210060830)

[Figure S29. 36](#_Toc210060831)

[Figure S30. 36](#_Toc210060832)

[Figure S31. 37](#_Toc210060833)

[Figure S32. 37](#_Toc210060834)

[Figure S33. 38](#_Toc210060835)

[Figure S34. 38](#_Toc210060836)

[Figure S35. 39](#_Toc210060837)

[Figure S36. 39](#_Toc210060838)

[Figure S37. 40](#_Toc210060839)

[Figure S38. 40](#_Toc210060840)

[Figure S39. 41](#_Toc210060841)

[Figure S40. 41](#_Toc210060842)

[Figure S41. 42](#_Toc210060843)

[Figure S42. 42](#_Toc210060844)

[Figure S43. 43](#_Toc210060845)

[Figure S44. 44](#_Toc210060846)

[Figure S45. 44](#_Toc210060847)

[Figure S46. 45](#_Toc210060848)

[**Supporting References** 46](#_Toc210060849)

**Supporting Methods**

**Chemicals**

Anhydrous sodium molybdate (Na_2_MoO_4_), rhodamine B (RhB), and urea (NH_2_CONH_2_) were purchased from Alfa Aesar. Calcium chloride (CaCl_2_), chlorhexidine (CHX), 5,5-dimethyl-1-pyrroline N-oxide (DMPO), 4-amino-2,2,6,6-tetramethylpiperidine (TEMP), sodium dihydrogen phosphate (NaH_2_PO_4_), horseradish peroxidase (HRP) (≥150 units mg^-1^), and murexide (C_8_H_8_N_6_O_6_) were obtained from Aladdin. Ethanol absolute (CH_3_CH_2_OH), sulfuric acid (H_2_SO_4_), sodium hydroxide (NaOH), acetic acid (HAc), nitric acid (HNO_3_), hydrogen peroxide (H_2_O_2_), and sodium chloride (NaCl) were obtained from Beijing Chemical Industry Co., Ltd. Potassium chloride (KCl), Copper chloride dihydrate (CuCl_2_·2H_2_O), and disodium terephthalate (TA) were purchased from Macklin. Sodium acetate (NaAc) and methanol (CH_3_OH) were obtained from Sigma. Glucose (C_6_H_12_O_6_) was obtained from TCI. 3,3',5,5'-tetramethylbenzidine (TMB), crystal violet staining solution, free sulfhydryl assay kit with DTNB, cell counting kit-8 (CCK-8), 2',7'-dichlorodihydroflurescein diacetate (DCFH-DA) and propidium iodide (PI) were obtained from Beyotime. SYTO green was obtained from Keygen Biotechnology Co., Ltd. Singlet oxygen sensor green (SOSG) probe was provided by Meilun Biotechnology Co., Ltd. FITC labeled Concanavalin A (FITC-ConA) was obtained from Shanghai Maokang Biotechnology Co., Ltd. Yeast extract and tryptone were obtained from OXID. L-cysteine (L-Cys, C_3_H_7_NO_2_S) and agar were provided by KEHBIO. Sodium alginate (viscosity 200 ± 20 mpa.s) was obtained from Innochem. Tribromoethanol (Br_3_CCH_3_OH, 1.25%) was obtained from Nanjing Aibei Biotechnology Co., Ltd.

**Preparation of MoS_2_ NSs**

MoS_2_ nanosheets (NSs) were prepared using a facile hydrothermal method. Typically, 100 mg of Na_2_MoO_4_ and 200 mg of L-Cys were dissolved in 30 mL of deionized water under stirring. HCl (0.1 M) was used to adjust the pH value to 5.6. Then, the solution was placed in a Teflon-lined autoclave and maintained at 200℃ for twenty-four hours. After the reaction, the obtained products were purified by washing with H_2_O and CH_3_CH_2_OH.

**Synthesis of MoCC SAzymes**

To prepare the Cys–Cu–Cys complex, a L-Cys : Cu molar ratio of 2 : 1 was adopted. Specifically, 24.2 mg of L-Cys (0.2 mmol) and 17.1 mg of CuCl_2_·2H_2_O (0.1 mmol) were dissolved in 10 mL of deionized water in a flat-bottomed beaker. The mixture was then subjected to ultrasound treatment (40 kHz, 150 W) at room temperature for 10 min. The resulting solution was centrifuged , and the precipitate was washed three times with deionized water. Finally, the purified Cys–Cu–Cys was resuspended in 10 mL deionized water for further use.

To obtain Cu single-atom loaded MoS_2_ nanozyme (MoCC SAzymes) with different Cu loading ratios, 8 mg (0.05 mmol), 16 mg (0.1 mmol), or 32 mg (0.2 mmol) of MoS_2_ NSs were respectively added into 10 mL of pre-prepared Cys-Cu-Cys solutions containing 0.1 mmol of Cu. The mixtures were gently stirred at room temperature for 1 h. The resulting products with Cys-Cu-Cys:MoS_2_ molar ratios of 2:1, 2:2, and 2:4 were designated as MoCC_2:1_, MoCC_2:2_ (MoCC for short), and MoCC_2:4_, respectively. After reactions, the suspensions were centrifuged and washed three times with deionized water and ethanol. The final products were freeze-dried for later use.

**Characterizations**

Morphology of as-obtained products was observed by scanning electron microscopy (SEM, S-4800, Hitachi, Japan). Point scan distributions of the Mo, S and Cu elements in MoCC were obtained by an energy dispersive X-ray spectrometer (EDS) equipped on S-4800. Transmission electron microscopy (TEM, TECNAI F20, USA) was applied to investigate the 2D structures and interfaces based on elemental mapping, selective area electron diffraction (SAED) and high-resolution TEM (HR-TEM) image of samples. Atomic resolution images of samples were recorded by an aberration-corrected TEM (AC-TEM, JEM ARM-200F, JEOL, Japan). Crystal phase and chemical compositions were studied by X-ray powder diffraction (XRD, D8 Advance, Bruker, USA), with Cu K_α_ radiation, and Raman spectroscopy (T64000, Horiba, Japan). X-ray photoelectron spectroscopy (XPS, ESCALAB 250Xi, Thermo Fisher, USA) and Fourier transform infrared spectroscopy (FT-IR, Nicolet iS10, Thermo fisher, USA) were used to analyze the surface structures. The Cu loading ratios in MoCC_2:1_, MoCC_2:2_, and MoCC_2:4_ were determined by inductively coupled plasma mass spectrometer (ICP-MS, Elemental X7, Thermo Scientific, USA). Hydrodynamic diameters and ζ potential were determined by dynamic light scattering (DLS, Omni, Brookhaven, USA). The optical absorbance was measured by Ultraviolet-Visible-Near infrared (UV-Vis-NIR) spectrophotometer (U-3900, Hitachi, Japan) and microplate reader (MK3, Thermo Fisher, USA). Fluorescence (FL) was detected by FluoroLog-3 optical system (Horiba, Japan). Piezo-response force microscope (PFM, MFP-D-SA-DV, Asylum Research, UK) was applied to determine the piezoelectric performance by alternating voltage across the samples. A 10 W 50 KHz portable ultrasound device (NSE-UPH-I, Nasonic, China) with a 1.0 cm^2^ acoustic head was applied to perform US treatment in vitro.

**Chemical speciation analysis**

For analysis of the chemical speciation of Mo and Cu in the samples, the 31109.02.BSRF.1W1B beamline at Beijing [Synchrotron Radiation](https://www.sciencedirect.com/topics/chemistry/synchrotron-radiation) Facility (BSRF) was used to investigate X-ray adsorption spectroscopy (XAS) of Cu *K*-edge and Mo *K*-edge, including extended X-ray adsorption fine structure (EXAFS) and X-ray adsorption near-edge structure (XANES). The powder samples were either pressed into disks with a diameter of 1 cm or evenly adhered to adhesive tapes. The EXAFS and XANES data were collected by transmission mode with a seven-element Ge detector.

**Ellman’s assay**

Samples were prepared in PBS at the following concentrations: MoS_2_ (0.1 mmol L^-1^), L-Cys (0.2 mmol L^-1^), Cys–Cu–Cys (0.1 mmol L^-1^, calculated based on Cu content), and MoCC (0.1 mmol L^-1^, calculated based on Cu content). According to the kit instructions, 2 mg of DTNB was dissolved in 0.5 mL of Assay Buffer and thoroughly mixed to obtain the DTNB stock solution. The working Ellman's Reagent Solution was prepared by diluting the DTNB stock with Assay Buffer at a 1:35 ratio. For the assay, 100 μL of each sample was mixed with 900 μL of Ellman’s Reagent Solution and incubated at room temperature for 15 min. PBS was used as the blank control. Absorbance at 412 nm was measured using a microplate reader.

**Cu single-atom enhanced POD-like activity and US-amplified catalytic activity**

The TMB colorimetric assay was employed to detect peroxidase (POD)-like activity of MoCC, which facilitates the decomposition of H_2_O_2_ to produce hydroxyl radicals (·OH). These radicals subsequently oxidize the TMB substrate, resulting in the formation of blue oxidized TMB (oxTMB) with a characteristic absorption peak at 652 nm. To compare the impact of different Cu loading ratios (16.51% for MoCC_2:1_, 10.11% for MoCC_2:2_ (MoCC), and 7.76% for MoCC_2:4_) on ·OH generation, reactions were conducted at 37℃ for 5 min in 0.1 M sodium acetate buffer (pH 4.7) with working concentrations of 1 mM TMB, 1 mM H_2_O_2_, and 33 μg mL^-1^ of either MoS_2_ (control), MoCC_1:2_, MoCC_2:2_, or MoCC_4:2_. After centrifugation, the supernatants were collected for measurement of optical density at 652 nm.

To compare the catalytic performance of MoCC with that of natural HRP, their corresponding specific activity (SA, U mg^-1^) was calculated. Typically, MoCC or HRP was added to a pH 4.7 HAc/NaAc buffer. The total masses of MoCC or HRP were 0.2, 0.4, 0.6, 0.8, and 1.0 µg, respectively, with the working concentrations of 1 mM TMB and 1 mM H_2_O_2_. The OD_652_ was measured over a period of 5 min using time-scan mode at room temperature. For each MoCC or HRP mass, the absorbance-time curves were plotted. For each curve, the initial linear part (where the coefficient of determination, R², was close to 1) was selected. The enzyme activity, expressed in units (U), was calculated from the slope of this linear portion. One U equals to the amount of enzyme needed to catalyze one micromole substrate in one minute. The SA was determined by dividing enzyme activity by the enzyme mass.

Additional assays were applied to investigate the effect of temperature, pH, and storage conditions on enzyme activities of MoCC and natural HRP. First, the enzymatic reactions were carried out at settled temperatures: 16°C, 25°C, 30°C, 37°C, 40°C, 45°C, 55°C, and 65°C. The reactions were performed in 0.1 M pH 4.7 sodium acetate buffer. For MoCC, the reaction system contained TMB (1 mM) and H_2_O_2_ (1 mM). For HRP, it consisted of 1 mM TMB and 0.1 mM H_2_O_2_. The quantities of HRP and MoCC were adjusted to ensure enzymatic activity equivalent to (U) in each reaction system. Then, the enzymatic reactions were conducted at different pH values: 2.4, 3.6, 4.7, 5.6, 6.1, 6.6, 7.4, and 8.2. The HAc/NaAc buffer was used for the reactions. The amounts of HRP and MoCC, as well as the reaction systems, were maintained consistent with those in the temperature experiments.

For the storage study, HRP and MoCC were stored under four different conditions: pH 4.0 at 20°C, pH 4.0 at 80°C, pH 8.0 at 20°C, and pH 8.0 at 80°C. After 24 h of storage, the POD-like activity of each sample was measured in a reaction system identical to the previous experiments, performed at 37°C in pH 4.7 sodium acetate buffer. In all experiments, the OD_652_ was recorded using a UV-Vis spectrophotometer. The highest absorbance recorded for each enzyme was considered as 100% relative enzyme activity, and relative activity plots were generated based on these values.

The impact of MoCC concentrations (0-100 μg mL^-1^), and H_2_O_2_ concentrations (0-100 mM) on the POD-like performance of MoCC was investigated using TMB assay. To assess the ·OH generation ability of MoCC, the experimental groups were categorized as follows: (1) TMB, (2) TMB + H_2_O_2_, (3) MoCC, (4) TMB + MoCC, (5) H_2_O_2_ + MoCC, (6) TMB + H_2_O_2_ + MoS_2_ NSs (33 μg mL^-1^), (7) TMB + H_2_O_2_ + Cys-Cu-Cys (33 μg mL^-1^), and (8) TMB + H_2_O_2_ + MoCC (33 μg mL^-1^). Reactions were conducted at 37℃ in 3 mL of HAc/NaAc buffer (0.1 M, pH 4.7) with final concentrations of 1 mM TMB and 1 mM H_2_O_2_. After a 5-minute reaction, supernatants were collected by centrifugation and were tested by UV-Vis absorption spectrophotometer at 652 nm.

TA, an ·OH-specific indicator, was applied to detect ·OH. In the presence of ·OH, TA is reduced to 2-hydroxy terephthalic acid (TAOH), which has a specific emission of fluorescence at 435 nm. In 0.1 M, pH 4.7 sodium acetate buffer, reactions were conducted with TA (0.1 mM), H_2_O_2_ (0.1 mM), and 10 μg mL^-1^ MoCC. Experimental groups were set as follow: (1) TA, (2) H_2_O_2_, (3) TA + H_2_O_2_, (4) MoCC, (5) TA + MoCC, (6) MoCC + H_2_O_2_, (7) TA + H_2_O_2_ + MoS_2_ NSs (10 μg mL^-1^), (8) TA + H_2_O_2_ + Cys-Cu-Cys (10 μg mL^-1^), and (9) TA + H_2_O_2_ + MoCC. After being incubated at 37℃ for 12 h, the supernatants were collected by centrifugation for fluorescence measurement at 435 nm.

To study the US-enhanced catalytic performance, a 40.0 W, 50 kHz ultrasound vibration was applied for 0, 3, and 5 min in the corresponding groups, which were set as follows: (1) MoCC + US 3 min, (2) Cys-Cu-Cys + US 3 min, (3) TMB + H_2_O_2_ + US 3 min, (4) TMB + H_2_O_2_ + MoCC, (5) TMB + H_2_O_2_ + MoCC + US 3 min, (6) TMB + H_2_O_2_ + Cys-Cu-Cys + US 5 min, and (7) TMB + H_2_O_2_ + MoCC + US 5 min. The working concentrations were 1 mM for TMB, 1 mM for H_2_O_2_, and 33 μg mL^-1^ for both Cys-Cu-Cys and MoCC.

To detect ·OH, Electron spin resonance (ESR) spectra (Bruker E500, USA) were also conducted, with DMPO serving as trapping agent. In a quartz capillary, 10 mM DMPO and 100 mM H_2_O_2_ were added into 0.1 M sodium acetate buffer (pH 4.7), in the presence of 250 μg mL^-1^ of Cys-Cu-Cys, MoS_2_ or MoCC, and then the ESR spectra were recorded at room temperature.

Enzyme-catalyzed kinetic studies were performed at room temperature using the time-scan mode to measure the absorption of oxTMB at 652 nm over 15 min. In 0.1 M, pH 4.7 sodium acetate buffer, reactions were carried out with 33 μg mL^-1^ of MoS_2_ or MoCC. The working concentrations of H_2_O_2_ ranged from 1.0 to 20.0 mM, with 1.0 mM TMB as the other substrate. Similarly, 0.3 to 2.0 mM of TMB were used with 1.0 mM H_2_O_2_ as the other substrate. For piezo-enhanced steady-state kinetic measurements, a 5 min of US treatment was applied before the measurement. Absorbance-time curves were plotted at 652 nm, and initial velocities were calculated from the linear portion of each curve. The Michaelis constant (*K_m_*) and maximum velocity (*V_max_*) were determined by fitting the data to the Lineweaver-Burk double-reciprocal equation:

$$V\text{=}\frac{V_{max}+\text{[S]}}{K_{m}+\text{[S]}}$$

$$\frac{1}{V}=\frac{K_{m}}{V_{max}}\cdot\frac{1}{\text{[S]}}+\frac{1}{V_{max}}$$

Here, *V* represents the initial velocity, *K_m_* represents the Michaelis constant, [S] is the concentration of substrate, and *V_max_* is the maximal reaction velocity.

**Catalase-like activity measurement**

To compare the catalase (CAT)-like performance of MoCC with different Cu loading ratios in decomposing H_2_O_2_ into O_2_, 150 μL of H_2_O_2_ (20 mM) were added to 0.1 M, pH 4.7 sodium acetate buffer at 37℃. The final concentrations of MoCC_2:1_, MoCC_2:2_, MoCC_2:4_, or MoS_2_ were 33 μg mL^-1^. A dissolved oxygen meter (JPSJ-605F, Leici, China) was applied to record the generation of O_2_. Additionally, photographs of O_2_ generation were captured in a 24-well plate at pH 4.7 and pH 7.4.

To investigate the influence of pH on O_2_ generation, 33 μg mL^-1^ MoCC was added to HAc/NaAc buffer with pH ranging from 3.0 to 9.0.

To compare the O_2_ generation capability of MoCC with MoS_2_, and Cys-Cu-Cys, the reactions were conducted in 0.1 M, pH 4.7 sodium acetate buffer at 37℃, with a working concentration of 1.0 mM H_2_O_2_ and 33 μg mL^-1^ for MoS_2_, Cys-Cu-Cys, or MoCC.

For the kinetic analysis of the CAT-like activity of MoCC, reactions were carried out within 10-min catalysis period. H_2_O_2_ with final concentrations ranging from 0 to 30 mM (0, 0.5, 1.0, 2.0, 5.0, 10.0, 20.0, 30.0 mM) were added to 0.1 M, pH 4.7 sodium acetate buffer in the presence of 33 μg mL^-1^ MoCC. Reaction velocities at each H_2_O_2_ concentration were calculated. Experimental data were fitted with Michaelis-Menten equations to calculate the *V_max_* and *K_m_* values for the CAT-like activity of MoCC.

**Detection of sono-piezocatalysis triggered ^1^O_2_**

Singlet oxygen sensor green (SOSG) probe was utilized to detect singlet oxygen (^1^O_2_). SOSG reacts with ^1^O_2_ and produces fluorescence emission at 530 nm when excited at 488 nm. The reactions were conducted with final concentrations of 50 μg mL^-1^ MoCC, and 0.05 mM SOSG in 0.1 M, pH 4.7 sodium acetate buffer. The effect of US on the generation of ^1^O_2_ of MoCC was studied at various US durations (0, 0.5, 1.0, 3.0, and 5.0 min).

To compare the sono-piezocatalysis triggered generation of ^1^O_2_ by MoCC with Cys-Cu-Cys, and MoS_2_, the following experimental groups were set up: (1) control (pH 4.7 HAc/NaAc buffer) + SOSG, (2) Cys-Cu-Cys + SOSG, (3) MoS_2_ + SOSG, (4) MoCC + SOSG, (5) control + SOSG + US, (6) Cys-Cu-Cys + SOSG + US, (7) MoS_2_ + SOSG + US, and (8) MoCC + SOSG + US. The US groups received 5 min of US irradiation before measurement. The final concentrations of MoS_2_, Cys-Cu-Cys, and MoCC were 50 μg mL^-1^ each, while the working concentration of SOSG was 0.05 mM. After a 30-min incubation, the fluorescence signal at 535 nm was measured.

ESR spectroscopy was performed to detect ^1^O_2_ using TEMP as a trapping agent, and 5 experimental groups were established: (1) ultrasound (US), (2) Cys-Cu-Cys, (3) Cys-Cu-Cys + US, (4) MoS_2_ + US, and (5) MoCC + US. In a quartz capillary, 10 mM TEMP was added into 0.1 M sodium acetate buffer (pH 4.7), in the presence of 250 μg mL^-1^ of Cys-Cu-Cys, MoS_2_ or MoCC, and then the ESR spectra were recorded at room temperature. The US groups were subjected to ultrasound vibration for 5 min prior to measurement.

***S. mutans* culture**

*Streptococcus mutans* (*S. mutans*) strain UA159 was utilized as a representative pathogenic bacterium associated with dental caries. To culture *S. mutans*, a glycerol stock solution was added to ultra-filtered tryptone-yeast extract (UFYTE) broth at a ratio of 1:100. After three passages, *S. mutans* was streaked onto UFTYE agar plates using an inoculation loop to obtain single colonies at 37℃, with 5% CO_2_. Single colony was inoculated into UFYTE and cultured to logarithmic phase in advance before each experiment.

**In vitro bacterial killing assay**

To investigate the effect of Cu ions released from MoCC on bacterial activity, the MoCC in HAc-NaAc buffer (pH 4.7, simulating oral acid microenvironment) for different time intervals (1, 12, and 24 h) were centrifuged and the supernatant was collected. Cu ions present in the supernatant were quantified using ICP-MS. After adding H_2_O_2_ (1.0 mM) and 50 μL of the supernatants to *S. mutans* (10^6^ CFU mL^-1^), the mixtures were cultured at 37 ℃, 5% CO_2_ for 1 h and were inoculated onto UFTYE medium.

To evaluate the bacterial killing efficacy of MoCC in the absence or presence of ultrasound in vitro, 10 experimental groups were established: (1) control (pH 4.7 0.1 M sodium acetate buffer), (2) H_2_O_2_, (3) H_2_O_2_ + Cys-Cu-Cys, (4) H_2_O_2_ + MoS_2_, (5) H_2_O_2_ + MoCC, (6) control + US, (7) H_2_O_2_ + US, (8) H_2_O_2_ + Cys-Cu-Cys + US, (9) H_2_O_2_ + MoS_2_ + US, and (10) H_2_O_2_ + MoCC + US. The concentrations of *S. mutans* and H_2_O_2_ were 10^6^ CFU mL^-1^ and 1.0 mM, respectively. The concentrations of Cys-Cu-Cys, MoS_2_, and MoCC were maintained at 50 μg mL^-1^. *S. mutans* were diluted in sterile PBS, and the treatments were conducted in UFTYE broth (pH = 4.7). After 1-h of incubation, groups (1) to (5) were inoculated onto UFTYE agar plates. For the US groups, the mixtures were subjected to 50 KHz ultrasound treatment prior to plating. All plates were cultured at 37 ℃, 5% CO_2_ for one day.

**Bacterial morphology**

To investigate morphology changes of bacterial cells after undergoing the POD-like activity synergistic cascaded sono-piezocatalysis effect of MoCC, bacterial cultures were treated with the following groups: (1) control (pH 4.7 0.1 M sodium acetate buffer), (2) H_2_O_2_, (3) H_2_O_2_ + Cys-Cu-Cys, (4) H_2_O_2_ + MoS_2_, (5) H_2_O_2_ + MoCC, (6) control + US, (7) H_2_O_2_ + US, (8) H_2_O_2_ + Cys-Cu-Cys + US, (9) H_2_O_2_ + MoS_2_ + US, and (10) H_2_O_2_ + MoCC + US. The working concentration of H_2_O_2_, Cys-Cu-Cys, MoS_2_, and MoCC were 1 mM, 50 μg mL^-1^, 50 μg mL^-1^, and 50 μg mL^-1^. For the US groups, a 50 KHz ultrasound vibration was applied for 10 min. After the treatment, bacterial samples were collected by centrifugation and washed three times with sterile PBS. The collected precipitates were then fixed with 4% polyoxymethylene for 4 h. Subsequently, the precipitates underwent sequential dehydration using ethanol with increasing concentrations: 30%, 50%, 70%, 80%, 90%, and 100%, with each concentration applied for 10 min. Finally, the bacterial morphology of dehydrated samples was carefully observed using SEM.

**SYTO/PI staining**

The live and dead cells staining assay of *S. mutans* was further used to investigate the POD-like activity synergistic cascaded sono-piezocatalysis effect of MoCC. The experimental groups were set as follow: (1) control (pH 4.7 0.1 M sodium acetate buffer), (2) H_2_O_2_, (3) H_2_O_2_ + Cys-Cu-Cys, (4) H_2_O_2_ + MoS_2_, (5) H_2_O_2_ + MoCC, (6) control + US, (7) H_2_O_2_ + US, (8) H_2_O_2_ + Cys-Cu-Cys + US, (9) H_2_O_2_ + MoS_2_ + US, and (10) H_2_O_2_ + MoCC + US. In brief, *S. mutans* (10^6^ CFU mL^-1^) was exposed to 50 μg mL^-1^ of Cys-Cu-Cys, MoS_2_, or MoCC, in the presence or absence of H_2_O_2_ (1 mM) in pH 4.7 sodium acetate buffer. The treated bacterial were then incubated at 37 ℃, 5% CO_2_ for 1 h. For the US-treated groups, the bacterial mixtures were ultrasonicated (50 kHz, 1.0 W cm^-2^, 50% duty cycles) for 30 min. After centrifugation (8,000 rpm, 1 min), the bacteria were collected and SYTO (20 μM) and PI (30 μM) were used to stain the resulting precipitates for 30 min. Sterile PBS was used to wash the stained bacteria to remove excess dye. Finally, confocal laser scanning microscope (CLSM) (A1R-Si, Nikon, Japan) was applied for observation.

**ROS detection**

DCFH-DA, a cell-permeable fluorescent probe, was selected to detect reactive oxygen species (ROS). Once inside bacterial cells, the DCFH-DA is deacetylated to DCFH, which is subsequently oxidized by ROS to produce fluorescent DCF, allowing ROS quantification. Ten experimental groups were set as follow: (1) control (pH 4.7 0.1 M HAc/NaAc buffer), (2) H_2_O_2_, (3) H_2_O_2_ + Cys-Cu-Cys, (4) H_2_O_2_ + MoS_2_, (5) H_2_O_2_ + MoCC, (6) control + US, (7) H_2_O_2_ + US, (8) H_2_O_2_ + Cys-Cu-Cys + US, (9) H_2_O_2_ + MoS_2_ + US, and (10) H_2_O_2_ + MoCC + US. In brief, *S. mutans* (10^6^ CFU mL^-1^) was incubated with MoS_2_ (50 μg mL^-1^), Cys-Cu-Cys (50 μg mL^-1^), or MoCC (50 μg mL^-1^), in the presence or absence of H_2_O_2_ (150 μM) at 37 ℃, 5% CO_2_ for 4 h. For US-treated groups, the solutions underwent ultrasonication (50 kHz, 1.0 W cm^-2^, 50% duty cycle) for 30 min. After centrifugation, mixtures were collected. The collected precipitates were added with 20 μM DCFH-DA and incubated for 20 min. Subsequently, sterile PBS was used to wash samples before CLSM observation.

The generation of ^1^O_2_ within bacterial cells was detected using SOSG under CLSM as well, and the groups were set as follows: (1) control (pH 4.7 0.1 M HAc/NaAc buffer), (2) Cys-Cu-Cys, (3) MoS_2_, (4) MoCC, (5) Control + US, (6) Cys-Cu-Cys + US, (7) MoS_2_ + US, and (8) MoCC + US. *S. mutans* (10^6^ CFU mL^-1^) was incubated with MoS_2_, Cys-Cu-Cys, or MoCC (50 μg mL^-1^) at 37 ℃, 5% CO_2_ for 3 h. The samples of US groups were applied ultrasound vibration (50 kHz, 1.0 W cm^-2^, 50% duty cycle) for 30 min. The mixtures were collected by centrifugation and the precipitates were then subjected to incubation with SOSG probe (5 μM) for 20 min at 37 ℃. The samples were washed with sterile PBS before CLSM observation.

**Anti-biofilm efficiency in vitro**

Mid-log phase *S. mutans* were cultured in UFTYE broth and inoculated into a 24-well plate and cultured at 37℃ with 5% CO_2_ until the biofilm can be observed. Every 24 h, biofilms were washed gently with sterile PBS and replaced with fresh UFTYE. The biofilms were subjected to the following treatments: (1) control (pH 4.7 0.1 M sodium acetate buffer), (2) H_2_O_2_, (3) H_2_O_2_ + Cys-Cu-Cys, (4) H_2_O_2_ + MoS_2_, (5) H_2_O_2_ + MoCC, (6) control + US, (7) H_2_O_2_ + US, (8) H_2_O_2_ + Cys-Cu-Cys + US, (9) H_2_O_2_ + MoS_2_ + US, and (10) H_2_O_2_ + MoCC + US. Biofilms were incubated with these groups for 1 h, with US-treated groups receiving ultrasound treatment (50 kHz, 1.0 W cm^-2^, 50% duty cycle) for 30 min and then washed with sterile PBS gently. The washed biofilms were fixed with methanol for 15 min and air-dried.

For semi-quantitative biofilm assessment, biofilms were washed with PBS for 3 times before adding 0.2% crystal violet and incubated for 10 min. Then, the crystal violet was discarded, and photographs of each well were taken. To semi-quantify the viable biofilms after various treatments, 33% glacial acetic acid was used, and the OD_590_ was recorded (MK3, Thermo Fisher, USA). Additionally, biofilms from each treatment group freeze-dried to obtain dry weight.

Polysaccharide content within the biofilms was quantified using phenol-H_2_SO_4_ method. In this process, mature biofilms in microplate were treated sequentially with 5% phenol solution and concentrated H_2_SO_4_. The mixtures were thoroughly homogenized, and OD_490_ was measured. Polysaccharide concentrations in each biofilm sample were determined by extrapolating absorbance values against glucose calibrated standard curve.

To assess the live/dead ratio within the biofilm after various treatments, mature biofilms were grown in confocal dishes and stained with 20 μM SYTO and 30 μM PI for 30 min. Excess dye was removed by washing with PBS prior to observation using CLSM.

To examine the degradability of extracellular polymeric substance (EPS) induced by the ROS storm from the MoCC catalytic process, mature biofilms were prepared, and free-floating bacteria were carefully removed using phosphate buffer saline. FITC-ConA (10 μg mL^-1^) was applied to stain the polyoxymethylene (4%) fixed biofilms for 0.5 h. Excess FITC-ConA was washed with PBS before observation using CLSM.

**RhB degradation assay**

Rhodamine B (RhB) was utilized to evaluate the enhanced POD-like performance synergistic cascaded sono-piezocatalysis effect of MoCC in dye degradation. The groups were designated as follows: (1) control (pH 4.7 0.01 M PBS), (2) H_2_O_2_, (3) H_2_O_2_ + MoCC, (4) control + US, (5) H_2_O_2_ + US, and (6) H_2_O_2_ + MoCC + US. A solution containing 50 μg mL^-1^ MoCC and H_2_O_2_ (50 mM) was mixed with RhB solution (20 μg mL^-1^), incubating at 37℃, and US irradiation (50 kHz, 1.0 W cm^-2^, 50% duty cycle) was applied to the US-treated groups. After being incubated for different times (0, 6, 12, and 24 h), the mixtures were collected by centrifugation (12,000 rpm for 1 min), and the OD_554_ of the supernatants was recorded. The degradation rate was evaluated by A/A_0_, where A and A_0_ were the residual and control OD_554_, respectively, at 24 h. Photographs of each group were recorded by iPhone (14 Pro, Apple, USA) in Pro-Raw mode.

**Biofilms morphology**

To simulate the growth of oral biofilm in vitro, tooth slices were fixed in each well of a 24-well plate, with each well containing *S. mutans* (10^6^ CFU mL^-1^) for 48h, at 37℃ with 5% CO_2_, and medium was replaced every 24 h. After the biofilms matured, the following treatment groups were designated: (1) control (pH 7.4 0.01 M phosphate buffer saline buffer), (2) H_2_O_2_, (3) H_2_O_2_ + Cys-Cu-Cys, (4) H_2_O_2_ + MoS_2_, (5) H_2_O_2_ + MoCC, (6) control + US, (7) H_2_O_2_ + US, (8) H_2_O_2_ + Cys-Cu-Cys + US, (9) H_2_O_2_ + MoS_2_ + US, and (10) H_2_O_2_ + MoCC + US. Mature biofilms on the tooth slices were subjected to treatments with Cys-Cu-Cys (50 μg mL^-1^), MoS_2_ (50 μg mL^-1^), and MoCC (50 μg mL^-1^), with or without H_2_O_2_ (1.0 mM), with the negative control group receiving only PBS. In the US-treated groups, a 50 kHz ultrasound was applied at 1 W cm^-2^ with a 50% duty cycle. After treatment, biofilms were fixed overnight with 4% polyoxymethylene and sequentially dehydrated. The dehydrated biofilms were observed using SEM to assess the surface morphology.

**Teeth whitening experiments in vitro**

Teeth were extracted from donors with informed consent and washed with purified water. Non-caries teeth were selected, and soft tissues were removed before being soaked in artificial saliva for one hour. Artificial saliva, which was composed of NaCl 0.4 g L^-1^, KCl 0.4 g L^-1^, NaH_2_PO_4_ 0.69 g L^-1^, CaCl_2_ 0.79 g L^-1^, urea 1.0 g L^-1^, was used to simulate the environment of human oral cavity^[1]^. To create experimental stained teeth models, all teeth were immersed in a solution of coke, coffee, and black tea for a period of 2 weeks. Before bleaching, purified water was used to wash teeth thoroughly until the rinse water ran clear. The whitening experiment was divided into the same groups as the biofilm degradation group above. The working concentrations of H_2_O_2_, MoS_2_, Cys-Cu-Cys, and MoCC were maintained at 0.3 M (1%), 50 μg mL^-1^, 50 μg mL^-1^, and 50 μg mL^-1^, respectively. All groups were incubated at 37℃ for 2 h, and 5 min ultrasound treatment (50 kHz, 1 W cm^-2^, 50% duty cycle) was applied to group (6) to (10) at each time point. Photographs of treated teeth were recorded by iPhone in Pro-Raw mode. Sampling and quantitative characterization of the color of tooth enamel was carried out under the guide of the Commission Internationale De L’Eclairage (CIELab) system, as described by the following equation:

$$\Delta E=\sqrt{(\Delta{L)}^{2}+(\Delta{a)}^{2}+(\Delta{b)}^{2}}$$

where *L* demonstrates luminance (brightness) from 0 to 100, *a* demonstrates color values on the red-green axis from -128 to 128, *b* demonstrates color values on the blue-yellow axis from -128 to 128, and *ΔE* was calculated to describe the effect of tooth whitening.

**In vitro cytotoxicity**

Human umbilical vein endothelial cells (HUVECs) and human keratinocyte cells (HaCaT) were cultured at 37℃ with 5% CO_2_ in 10% fetal bovine serum (FBS) supplemented Dulbecco's Modified Eagle Medium (DMEM) with penicillin-streptomycin solution (1%). HUVECs and HaCaT cells were inoculated in 96-well plates with a density of 5 × 10^3^ cells/well and incubated at 37℃ with 5% CO_2_. After 24 h, the culture medium was replaced with fresh DMEM medium containing different concentrations of MoCC (0, 10, 25, 50, 100, 150, and 200 μg mL^-1^). After being co-incubated for one day, a cytotoxicity assay was applied.

**Cell migration test**

To assess the cell migration and proliferation induced by MoCC, a scratch assay was performed. Typically, in a six well plate, HUVECs were plated at a density of 3 × 10^5^ cells/well. Sterile pipette tips were applied to scratch cell layers along with a ruler to create four parallel lines in each well. Floating cell fragments were gently removed by rinsing the wells with PBS.

After that, MoS_2_ or MoCC (50 μg mL^-1^) were added, using DMEM with 2% FBS. The cells were then co-incubated for 0, 12, and 24 h. Inverted microscope (IX71, Olympus, Japan) was applied to photograph migrations of HUVEC at each time point.

**In vivo anti-caries model**

All in vivo experiments of this study were performed in accordance with the guidelines approved by biomedical ethics committee of Peking University (LA2022469). Male Sprague Dawley (SD) rats (21-day old) were obtained from Beijing Huafukang Biotechnology Co., LTD and housed within sterile barrier system.

The day on which the rats were 21-day old was designated as day 1 (d1) in the in vivo experiment. From d1 to day 3 (d3), rats were administered a regular diet along with antibiotic water containing penicillin (200 mg L^-1^) and streptomycin (1500 mg L^-1^) to prevent the presence of endogenous dental pathogens. On day 4 (d4), oral samples were collected using sterile swabs from each rat and then were inoculated onto MSB agar plates to verify the clearance of endogenous dental bacteria. SD rats that showed negative bacterial culture results were selected for subsequent experiments. From day 6 (d6) to day 8 (d8), mid-log phase suspensions of *Streptococcus mutans* (UA159) were applied twice daily onto the molars of SD rats. After inoculation, the rats were fasted for 30 min before being given cariogenic diet (Cariogenic Diet-2000, TrophicDiet, China) and 5% sucrose water. On day 9 (d9), 18 rats were confirmed to have colonization of *S. mutans* through mouth sampling.

During the period of animal experiment, body weights of each rat were recorded daily. In animal experiment, an ultrasound vibration probe (0816, PERSMAX, China) was applied (45 KHz) to stimulate the ultrasound toothbrush in daily teeth cleaning.

**In vivo dental caries treatments**

Sprague Dawley rats were randomly assigned to 6 groups (n = 3) as follows: (1) control (pH 7.4 PBS), (2) 1% H_2_O_2_, (3) MoCC, (4) 0.4 % CHX, (5) H_2_O_2_ + MoCC, (6) H_2_O_2_ + MoCC + US. The anesthetization of SD rats was carried out using 1.25% tribromoethanol by intraperitoneal (i.p.) injection (10 mL Kg^-1^) and subjected to the respective treatments. For groups (1), (2), and (4), 200 μL of PBS, 1% H_2_O_2_, and 0.4 % CHX was respectively dipped directly to the tooth surface. For groups (3), (5), and (6), MoCC was encapsulated in cross-linked 2% sodium alginate hydrogel assisted by Ca^2+^ ions and applied to the molar surface. Specifically, using micro applicators (MA01, Huanghua Promisee Dental Co., Ltd, China), 0.5 mL of 2% sodium alginate containing 100 μg mL^-1^ MoCC was evenly applied to each side of the molar surface. The treatments were administered on days 10, 11, 12, 14, 16, 18, 20, 22, 24, 26, 28, and 30. After treatments, the rats were fasted for 30 min and were provided with cariogenic diet and 5% sucrose water.

**Anti-caries efficiency in animal models**

Plate counting method was applied to evaluate anti-caries efficiency. Oral bacterial samples from each group were collected using sterile swabs on days 4, 9, 12, 18, 24, 30. The collected swabs were soaked in PBS and were diluted to be inoculated onto MSB agar plates at 37℃ with 5% CO_2_.

On the final day of the experiment, blood samples were collected for blood routine tests. Oral bacterial samples were collected for microbiota analysis by gently rubbing the oral cavity and tooth surface with sterile swabs to prevent bleeding, and they were stored in bacteria-free tubes. The bacterial samples were commissioned to Majorbio Bio-Pharm Technology Co. Ltd. (Shanghai, China). All SD rats were euthanized using CO_2_. Palates, upper jaws, tongues, teeth, and main organs were harvested and preserved. Soft tissues were dehydrated and embedded for Hematoxylin & Eosin (H&E) staining. Photographs of the molars were taken from a top-down view using iPhone in Pro-Raw mode. The morphology of tissue slices stained with H&E were imaged using an inverted microscopy.

**Micro-architecture and carious levels after MoCC treatments**

Spectral micro computed tomography (micro-CT) (μColor SA, Institute of High Energy Physics, CAS) was performed to compare the change of teeth density caused by dental caries among different groups. The upper molars were then stained using a 0.4% murexide solution and shaken (90 rpm) for 24 h. Then, a diamond coating tool was applied to section the teeth sagittally, followed by rinsing with deionized water. Carious levels were then assessed by two professionals under stereomicroscope based on the Keyes’ score principle.

**Hemolysis assay**

Red blood cells (RBCs) from SD rats were harvested through centrifugation (10,000 rpm, 3 min) in anticoagulant tubes, and were diluted with phosphate buffer saline to obtain 20% RBCs solution. MoCC (1 mg mL^-1^) were added into 500 μL phosphate buffer saline containing 20% RBCs to obtain a series of final concentrations (10, 30, 60, 120, and 180 μg mL^-1^) of MoCC. Simultaneously, phosphate buffer saline and water were set as negative and positive groups, respectively. After 4-hour co-incubation at room temperature, the supernatants were collected by centrifugation (10,000 rpm, 3 min) to record OD_570_, and the ratio of hemolysis was calculated.

**Computational methods**

Structural simulations and energy calculations were carried out using density functional theory (DFT) within Vienna ab initio simulation package (VASP), employing the projector augmented plane-wave (PAW) method^[2]^. For the exchange-correlation potential, the Perdew-Burke-Ernzerhof (PBE) formulation within the generalized gradient approximation (GGA) was utilized^[3]^. A plane-wave basis set with a cut-off energy of 500 eV was chosen. The iterative solution of the Kohn-Sham equation was converged to an accuracy of 10⁻⁵ eV. Given variations in atomic composition and unit cell dimensions, the Brillouin zone was sampled using the Monkhorst–Pack scheme with a grid accuracy of 0.03 2𝜋 Å^−1^ across all configurations^[4]^. To mitigate spurious interactions between periodic images, a vacuum layer of 20 Å was introduced along the direction perpendicular to the sheet. Structural relaxation was performed until residual atomic forces were reduced to 0.02 eV/Å or lower.

The Cu single-atom enhanced POD-like activity of MoCC to degrade H_2_O_2_ pathways are performed as the following equations:

H_2_O_2_ + * → OH* + O* + H^+^ + e^-^; OH* + O* → •OH + O* (Eq. S1)

H_2_O_2_ + * → OH* + OH*; OH* + OH* → •OH + OH* (Eq. S2)

**Statistical analysis and graphics**

OriginPro v.9.8.200 was applied to conduct the data analysis in this study. Analysis of significant differences between multiple groups was based on Tukey’s multiple comparisons post-test of one-way ANOVA. Comparisons between two groups were carried out based on two-sided t-test. *, **, and *** indicate p < 0.05, p < 0.01, and p < 0.001, respectively.

Scheme 1, Figure 1 (a), Figure 7 (a) and Table of Contents Images were Created in BioRender. Com.

**Supporting Tables**

**Supporting Table S1.** XPS binding energy of the synthesized samples.

| **Peak** | **Binding energy (eV)** | | |
| --- | --- | --- | --- |
|  | **Cys-Cu-Cys** | **MoS_2_** | **MoCC** |
| Mo3d (IV) 5/2 | **/** | 227.6 | 227.3 |
| Mo3d (IV) 3/2 | **/** | 230.8 | 230.5 |
| Mo3d (VI) 5/2 | **/** | 231.7 | 231.6 |
| Mo3d (VI) 3/2 | **/** | 234.8 | 234.6 |
| Cu2p (I) 3/2 | 932.7 | **/** | 932.8 |
| Cu2p (I) 1/2 | 952.6 | **/** | 952.4 |
| Cu2p (II) 3/2 | 934.0 | **/** | 934.0 |
| Cu2p (II) 1/2 | 954.8 | **/** | 954.6 |

**Supporting Table S2.** EXAFS fitting parameters at the Cu K-edge (S_0_^2^=0.82)

| samples | path | C. N.^[a]^ | R (Å) ^[b]^ | σ^2^ (× 10^−3^ Å^2^) ^[c]^ | ΔE (eV) ^[d]^ | R factor^[e]^ |
| --- | --- | --- | --- | --- | --- | --- |
| Cys-Cu-Cys | Cu-S | 2.0±0.2 | 2.25±0.02 | 9.8±1.4 | 5.2±1.5 | 0.01 |
|  | Cu-S-C | 3.9±0.7 | 3.16±0.02 | 3.5±3.3 |  |  |
| MoCC | Cu-S | 1.8±0.2 | 2.23±0.02 | 9.6±1.1 | 3.9±1.1 | 0.01 |
|  | Cu-Mo | 1.0±0.2 | 2.72±0.02 | 3.4±1.9 |  |  |

*^a^*C. N.: coordination numbers; *^b^R*: bond distance; *^c^σ*^2^: Debye-Waller factors; *^d^*Δ*E*_0_: the inner potential correction. *^e^R* factor: goodness of fit.

**Supporting Table S3.** Comparison of kinetics parameters based on the concentration of Cu active sites in the MoCC with HRP and SAzymes.

| Enzymes/  Nanozymes | Substrate | [E] (M) | V_max_  (10^-7^ M S^-1^) | K_m_  (mM) | TOF  (10^-3^ S^-1^) | TOF/K_m_  (10^-4^ S^-1^ mM^-1^) | Ref. |
| --- | --- | --- | --- | --- | --- | --- | --- |
| HRP | H_2_O_2_ | / | 0.28 | 11.63 | / | / | [5] |
|  | TMB | / | 0.14 | 3.75 | / | / |  |
| NH-MoO_3_-x | H_2_O_2_ | / | 1.66 | 2.41 | / | / | [6] |
|  | TMB | / | 1.30 | 5.06 | / | / |  |
| Co-NC | H_2_O_2_ | / | 1.96 | 41.64 | / | / | [7] |
|  | TMB | / | 2.58 | 0.96 | / | / |  |
| Ni@Co-NC | H_2_O_2_ | / | 2.34 | 26.45 | / | / | [7] |
|  | TMB | / | 5.10 | 1.16 | / | / |  |
| Cu-NPs/N-C | H_2_O_2_ | 2.58×10^-5^ | 0.86 | 17.98 | 3.32 | 1.85 | [8] |
|  | TMB | 2.58×10^-5^ | 1.28 | 1.57 | 4.95 | 31.50 |  |
| PtFe | H_2_O_2_ | 3.72×10^-5^ | 0.82 | 217.60 | 2.20 | 0.10 | [9] |
|  | TMB | 3.72×10^-5^ | 0.60 | 0.24 | 1.62 | 68.28 |  |
| PtFe@Fe_3_O_4_ | H_2_O_2_ | 1.25×10^-4^ | 1.08 | 53.55 | 0.86 | 0.16 | [9] |
|  | TMB | 1.25×10^-4^ | 0.55 | 0.21 | 0.44 | 20.55 |  |
| MoS_2_  US OFF | H_2_O_2_ | / | 0.43 | 20.43 | / | / | This  work |
|  | TMB | / | 0.59 | 1.66 | / | / |  |
| MoS_2_  US ON | H_2_O_2_ | / | 0.91 | 2.92 | / | / | This  work |
|  | TMB | / | 0.92 | 1.70 | / | / |  |
| MoCC  (US OFF) | H_2_O_2_ | 5.3×10^-5^ | 2.21 | 0.76 | 4.17 | 54.87 | This  work |
|  | TMB | 5.3×10^-5^ | 1.76 | 1.35 | 3.32 | 24.50 |  |
| MoCC  (US ON) | H_2_O_2_ | 5.3×10^-5^ | 4.56 | 0.65 | 8.60 | 113.2 | This  work |
|  | TMB | 5.3×10^-5^ | 2.94 | 0.86 | 5.55 | 64.5 |  |

[E] is the molar concentration of active sites in enzyme or nanozyme concentration. *K_m_* is the Michaelis constant, *V_max_* is the maximal reaction velocity. Turnover frequency (TOF) is the turnover number (TON) per active site in unit time^[10, 11]^, where TOF= *V_max_*/[E].

**Supporting Table S4.** Comparative analysis of MoCC and representative MoS_2_-supported single-atom nanozymes

|  | MoCC | Cu@MoS_2_^[12]^ | Co–MoS_2_^[13]^ |
| --- | --- | --- | --- |
| Single atom site & coordination | 3D-biomimetic S-Cu-S single-atom catalytic sites | Cu single atom was adsorbed at the hollow site above the Mo atom | Single Co atom sat on top of the Mo atoms in 1T |
| Catalytic reaction type | POD-like | POD-like | POD-like |
| Kinetic parameters  (H_2_O_2_ as substrate) | K_m_= 0.65 mM  V_max_= 4.56×10^⁻7^ M s^⁻1^ | K_m_= 2.05 mM  V_max_= 1.09×10^⁻10^ M s^⁻1^ | K_m_= 3.35 mM  V_max_= 6.49×10^⁻7^ M s^⁻1^ |

**Supporting Table S5.** Comparison of MoCC with various Cu-based nanozymes

|  | Synthetic route | Active‑site | Catalytic performance |
| --- | --- | --- | --- |
| PdCu  nanocorals^[14]^ | One-pot microwave wet‑chemical growth of branched PdCu | surface Cu(0)/Pd(0) alloy | POD-like activity  K_m_ (TMB) = 0.25 mM  V_max_ (TMB) =1.19 × 10^-8^ M s⁻¹ |
| CuBi bimetallic aerogel^[15]^ | Chemical reduction + gelation (RT) | Bi^0^+ Cu^0^ dispersed in porous aerogel | POD-like activity, SA = 152 U mg^-1^  K_m_ (TMB) = 0.43 mM |
| GMP–Cu clusters^[16]^ | Self-assembly of Cu^2+^ with GMP under mild aqueous conditions | Cu atoms bridged by phosphate/nucleobase groups | Laccase-like activity  K_m_ (2,4-DP) = 0.59 mM  V_max_ (2,4-DP) =1.4 × 10^-5^ M s⁻¹ |
| CuO nanoparticles^[17]^ | Precipitation / thermal oxidation methods | Surface Cu–O motifs, mixed Cu^2+^/Cu^+^ sites | Laccase-like activity  K_m_ (2,4-DP) = 0.2 mM  V_max_ (2,4-DP) =1.1 × 10^-7^ M s⁻¹ |
| Cu_2_O nanozyme^[18]^ | wet-chemical alkaline precipitation method | Cu–O surface sites | / |
| NZ-Cu^[19]^ | Supramolecular self-assembly driven by chemical fuels | Non-Cu active motifs, arising from supramolecular interactions | Laccase-like activity  K_m_ (2,4-DP) = 0.12 mM  V_max_ (2,4-DP) =1.5 × 10^-9^ M s⁻¹ |
| CuN_4_^[20]^ | Pyrolysis of Cu precursors with N-doped carbon frameworks | Well-defined Cu–N₄ centers | POD-like activity  K_m_ (TMB) = 14.2 mM  V_max_ (TMB) = 1.3 × 10^-7^ M s⁻¹ K_m_ (H_2_O_2_) = 752 mM  V_max_ (H_2_O_2_) = 0.8 × 10^-7^ M s⁻¹ |
| CDPhs^[21]^ | Coordination-driven assembly with organic diphenyl ligands | Cu–ligand motifs with diphenyl coordination | POD-like activity  K_m_ (H_2_O_2_) = 14.7 mM  V_max_ (H_2_O_2_) = 1.78 × 10^-7^ M s⁻¹ |
| Cu-DCA NZs^[22]^ | Co‑assembly (RT, 4 h) of Cu^2+^ with 2,5‑dimercaptoterephthalic acid | Cu(I/II)–S₂O₂ chelates | Superoxide dismutase and catalase mimic activity |
| P/M-PANI-TA- Cu^2+[23]^ | Cu^2+^ doped into the helical PANI-TA supramolecular structure, assisted by TA to facilitate the binding and structural stability | Cu^2+^ centers | As peroxidase mimics towards the 3, 4-dihydroxy-S/R-phenylalanine (S/R-DOPA) oxidation |
| MoCC | Cys–Cu–Cys auto-assembly (RT, 1 h) onto MoS_2_ | 3D S–Cu–S single atoms | POD-like activity, SA = 355 U mg⁻¹  K_m_ (TMB) = 0.86 mM  V_max_ (TMB) = 2.94×10^-7^ M s⁻¹  K_m_ (H_2_O_2_) = 0.65 mM  V_max_ (H_2_O_2_) = 4.56 × 10^-7^ M s⁻¹ |

**Supporting Table S6.** Bader charge results of MoS_2_, MoCC, and Cys-Cu-Cys. “Elements” series and “Number” indicated the notation and serial number of corresponding atoms, respectively, in the nanozyme system.

| Element | Number | MoCC | Number | MoS_2_ | Number | Cys-Cu-Cys | Transferred charge |
| --- | --- | --- | --- | --- | --- | --- | --- |
| S1 | S26 | 6.246076 | - | - | S26 | 6.27321 | -0.027134 |
| S2 | S27 | 6.237063 | - | - | S27 | 6.259998 | -0.022935 |
| Cu | Cu25 | 10.524725 | - | - | Cu25 | 10.541958 | -0.017233 |
| C1 | C15 | 4.142463 | - | - | C15 | 4.135081 | 0.007382 |
| C2 | C18 | 4.12807 | - | - | C18 | 4.121702 | 0.006368 |
| S3 | S90 | 6.584679 | S63 | 6.599377 | - | - | -0.014698 |
| S4 | S92 | 6.590557 | S65 | 6.603313 | - | - | -0.012756 |
| S5 | S74 | 6.590506 | S47 | 6.599145 | - | - | -0.008639 |
| S6 | S76 | 6.613594 | S49 | 6.597383 | - | - | 0.016211 |
| S7 | S78 | 6.59161 | S51 | 6.599086 | - | - | -0.007476 |
| S8 | S94 | 6.592304 | S67 | 6.596423 | - | - | -0.004119 |
| S9 | S88 | 6.58999 | S61 | 6.597842 | - | - | -0.007852 |
| S10 | S58 | 6.600493 | S31 | 6.596997 | - | - | 0.003496 |
| S11 | S60 | 6.605308 | S33 | 6.598099 | - | - | 0.007209 |
| S12 | S62 | 6.604956 | S35 | 6.598674 | - | - | 0.006282 |
| S13 | S64 | 6.60082 | S37 | 6.597079 | - | - | 0.003741 |
| S14 | S72 | 6.600307 | S45 | 6.596078 | - | - | 0.004229 |
| S15 | S80 | 6.600918 | S53 | 6.596719 | - | - | 0.004199 |
| S16 | S104 | 6.596956 | S77 | 6.598531 | - | - | -0.001575 |
| S17 | S106 | 6.600851 | S79 | 6.5977 | - | - | 0.003151 |
| S18 | S108 | 6.597794 | S81 | 6.599123 | - | - | -0.001329 |
| Mo1 | Mo148 | 12.804021 | Mo121 | 12.802768 | - | - | 0.001253 |
| Mo2 | Mo149 | 12.803657 | Mo122 | 12.80153 | - | - | 0.002127 |
| Mo3 | Mo150 | 12.804409 | Mo123 | 12.801083 | - | - | 0.003326 |
| Mo4 | Mo151 | 12.801519 | Mo124 | 12.800757 | - | - | 0.000762 |
| Mo5 | Mo171 | 12.801707 | Mo144 | 12.801458 | - | - | 0.000249 |
| Mo6 | Mo172 | 12.802107 | Mo145 | 12.80177 | - | - | 0.000337 |
| Mo7 | Mo173 | 12.802531 | Mo146 | 12.802116 | - | - | 0.000415 |
| Mo8 | Mo163 | 12.802066 | Mo136 | 12.802092 | - | - | -0.000026 |
| Mo9 | Mo164 | 12.808404 | Mo137 | 12.803602 | - | - | 0.004802 |
| Mo10 | Mo165 | 12.801373 | Mo138 | 12.798234 | - | - | 0.003139 |
| Mo11 | Mo166 | 12.800987 | Mo139 | 12.801029 | - | - | -0.000042 |
| Mo12 | Mo155 | 12.803051 | Mo128 | 12.803275 | - | - | -0.000224 |
| Mo13 | Mo156 | 12.813258 | Mo129 | 12.810931 | - | - | 0.002327 |
| Mo14 | Mo157 | 12.807362 | Mo130 | 12.802072 | - | - | 0.00529 |
| Mo15 | Mo158 | 12.802097 | Mo131 | 12.800082 | - | - | 0.002015 |
| Mo16 | Mo159 | 12.801337 | Mo132 | 12.801578 | - | - | -0.000241 |
| Mo17 | Mo141 | 12.801627 | Mo114 | 12.801976 | - | - | -0.000349 |
| Mo18 | Mo142 | 12.800534 | Mo115 | 12.80028 | - | - | 0.000254 |
| Mo19 | Mo143 | 12.800687 | Mo116 | 12.80071 | - | - | -0.000023 |

**Supporting Table S7.** Bader charge before and after H_2_O_2_ absorbing to MoS_2_. “Elements” and the “Number” indicated the notation and serial number of corresponding atoms, respectively, in the MoS_2_-H_2_O_2_ system.

| Elements | Number | MoS_2_-H_2_O_2_ | Number | MoS_2_ | Number | H_2_O_2_ | Transferred charge |
| --- | --- | --- | --- | --- | --- | --- | --- |
| H1 | H1 | 0.405126 | - | - | H1 | 0.398372 | 0.006754 |
| H2 | H2 | 0.385219 | - | - | H2 | 0.38624 | -0.001021 |
| O1 | O3 | 6.598881 | - | - | O3 | 6.598757 | 0.000124 |
| O2 | O4 | 6.626304 | - | - | O4 | 6.616631 | 0.009673 |
| S3 | S67 | 6.586104 | S63 | 6.599377 | - | - | -0.013273 |
| S4 | S69 | 6.603271 | S65 | 6.603313 | - | - | -0.000042 |
| S5 | S51 | 6.571681 | S47 | 6.599145 | - | - | -0.027464 |
| S6 | S53 | 6.569726 | S49 | 6.597383 | - | - | -0.027657 |
| S7 | S55 | 6.59472 | S51 | 6.599086 | - | - | -0.004366 |
| S11 | S37 | 6.574307 | S33 | 6.598099 | - | - | -0.023792 |
| S12 | S39 | 6.580696 | S35 | 6.598674 | - | - | -0.017978 |

**Supporting Table S8.** Bader charge results before and after H_2_O_2_ absorbing to MoCC. “Elements” and “Number” series indicated the notation and serial number of corresponding atoms, respectively, in the MoCC-substrate system.

| Elements | Number | MoCC-H_2_O_2_ | Number | MoCC | Number | H_2_O_2_ | Transferred charge |
| --- | --- | --- | --- | --- | --- | --- | --- |
| H1 | H13 | 0.403371 | - | - | H1 | 0.39837 | 0.005001 |
| H2 | H14 | 0.394985 | - | - | H2 | 0.396465 | -0.00148 |
| O1 | O27 | 6.604811 | - | - | O3 | 6.601471 | 0.00334 |
| O2 | O28 | 6.586071 | - | - | O4 | 6.603694 | -0.017623 |
| Cu | Cu129 | 10.523472 | Cu25 | 10.524725 | - | - | -0.001253 |
| S1 | S29 | 6.27799 | S26 | 6.246076 | - | - | 0.031914 |
| S2 | S30 | 6.193515 | S27 | 6.237063 | - | - | -0.043548 |
| C1 | C17 | 4.141442 | C15 | 4.142463 | - | - | -0.001021 |
| C2 | C20 | 4.130667 | C18 | 4.12807 | - | - | 0.002597 |
| C3 | C15 | 3.6495 | C13 | 3.649859 | - | - | -0.000359 |
| C4 | C16 | 2.567229 | C14 | 2.565256 | - | - | 0.001973 |

**Supporting Figures**

**Figure S1.** Synthesis of Cys-Cu-Cys.


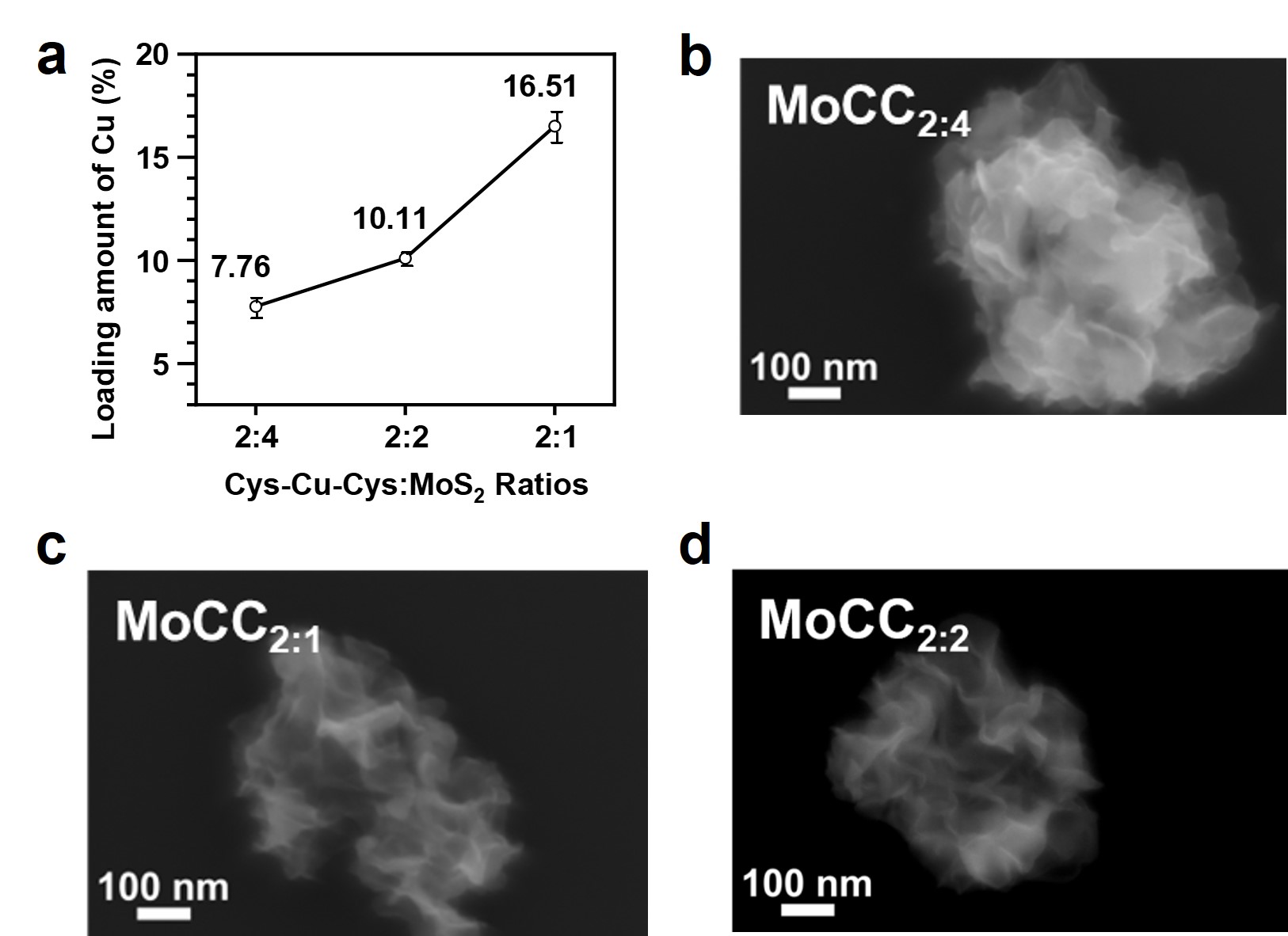


**Figure S2.** (a) Variable Cu loading ratios. SEM images of (b) MoCC_2:4_, (c) MoCC_2:1_, and (d) MoCC_2:2_ (MoCC).


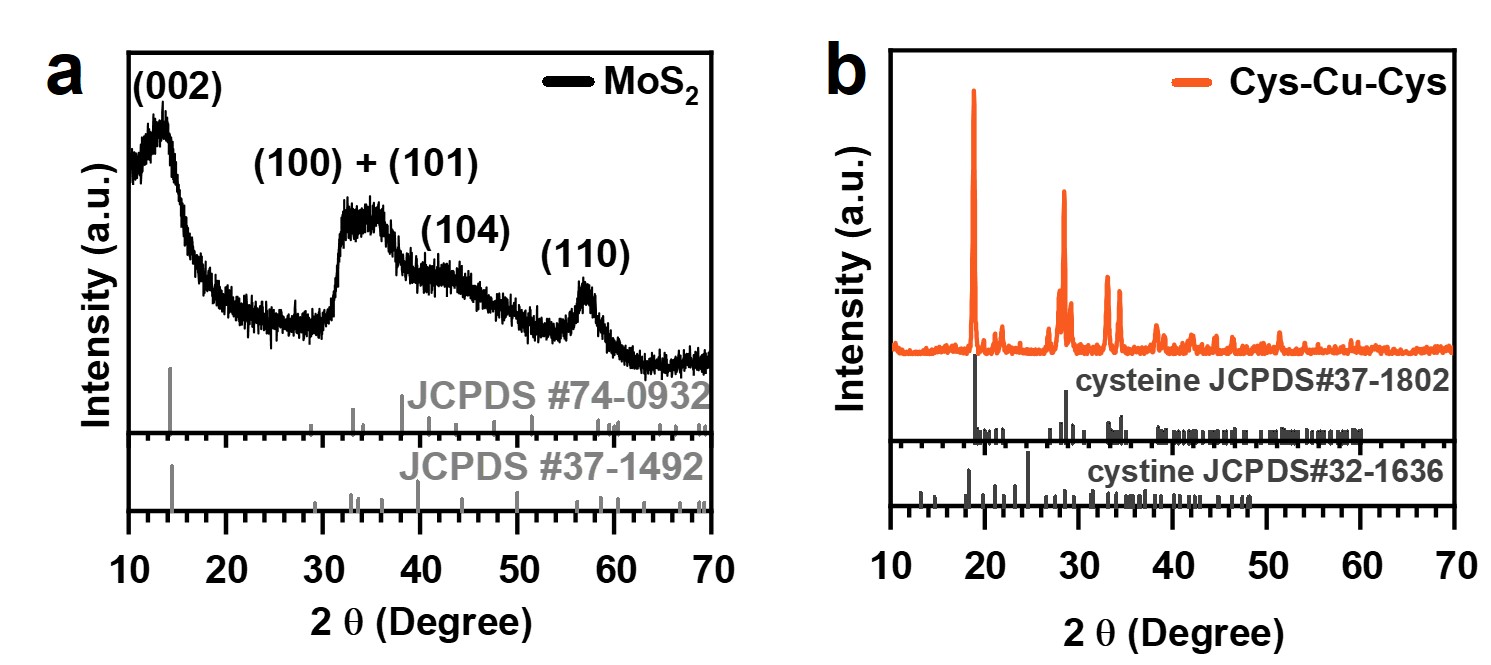


**Figure S3.** XRD patterns of (a) MoS_2_ and (b) Cys-Cu-Cys.


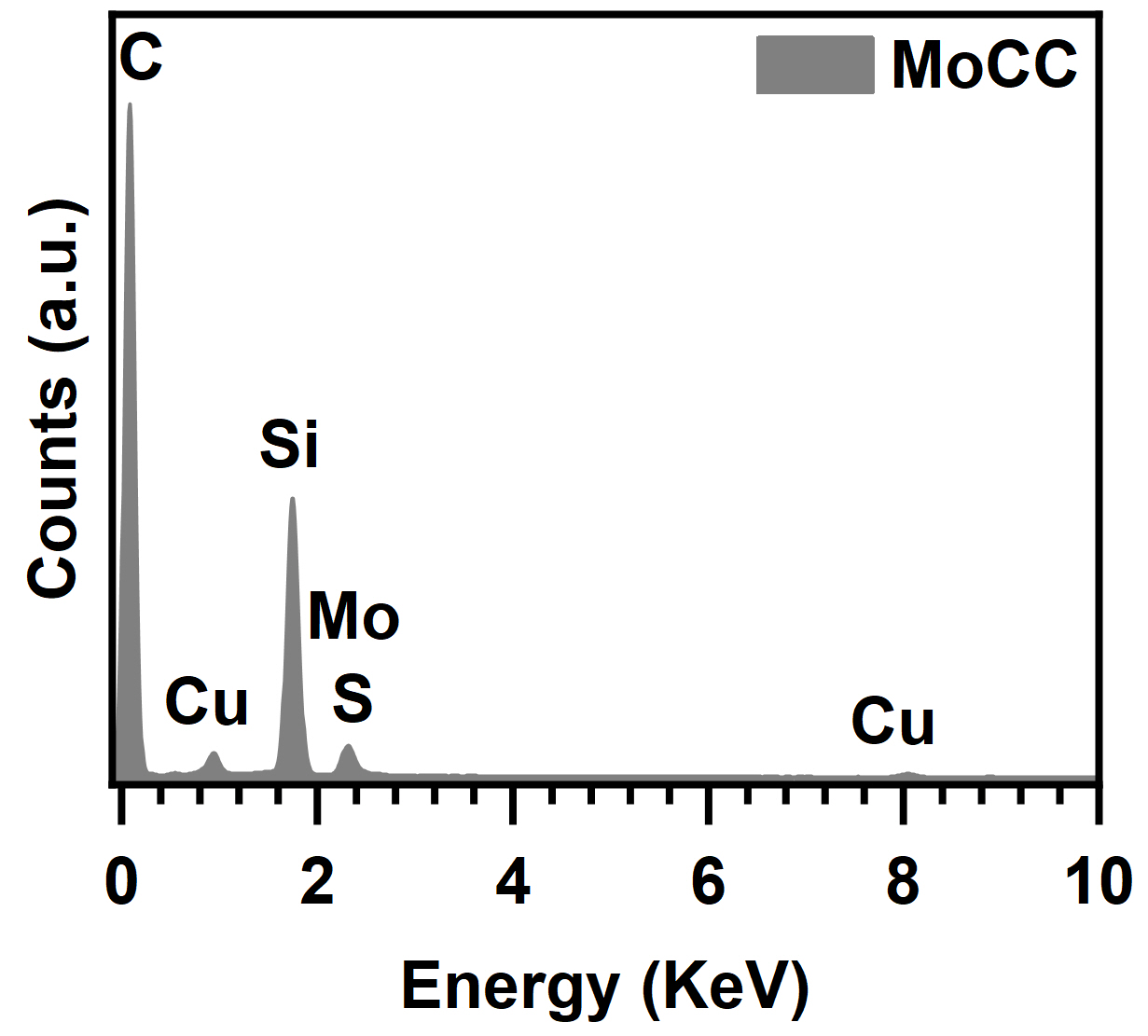


**Figure S4.** EDS of MoCC.


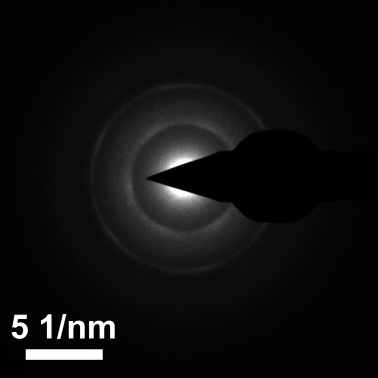


**Figure S5.** SAED pattern of MoCC.


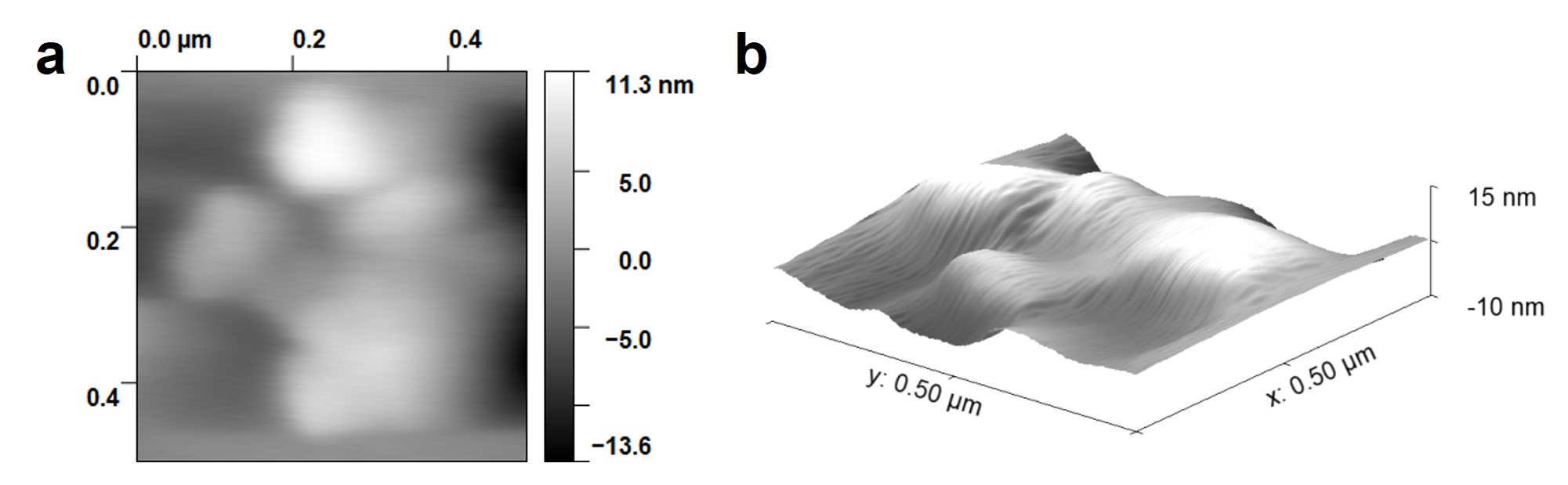


**Figure S6.** AFM images of (a) 2D image and (b) reconstructed 3D image of MoCC.


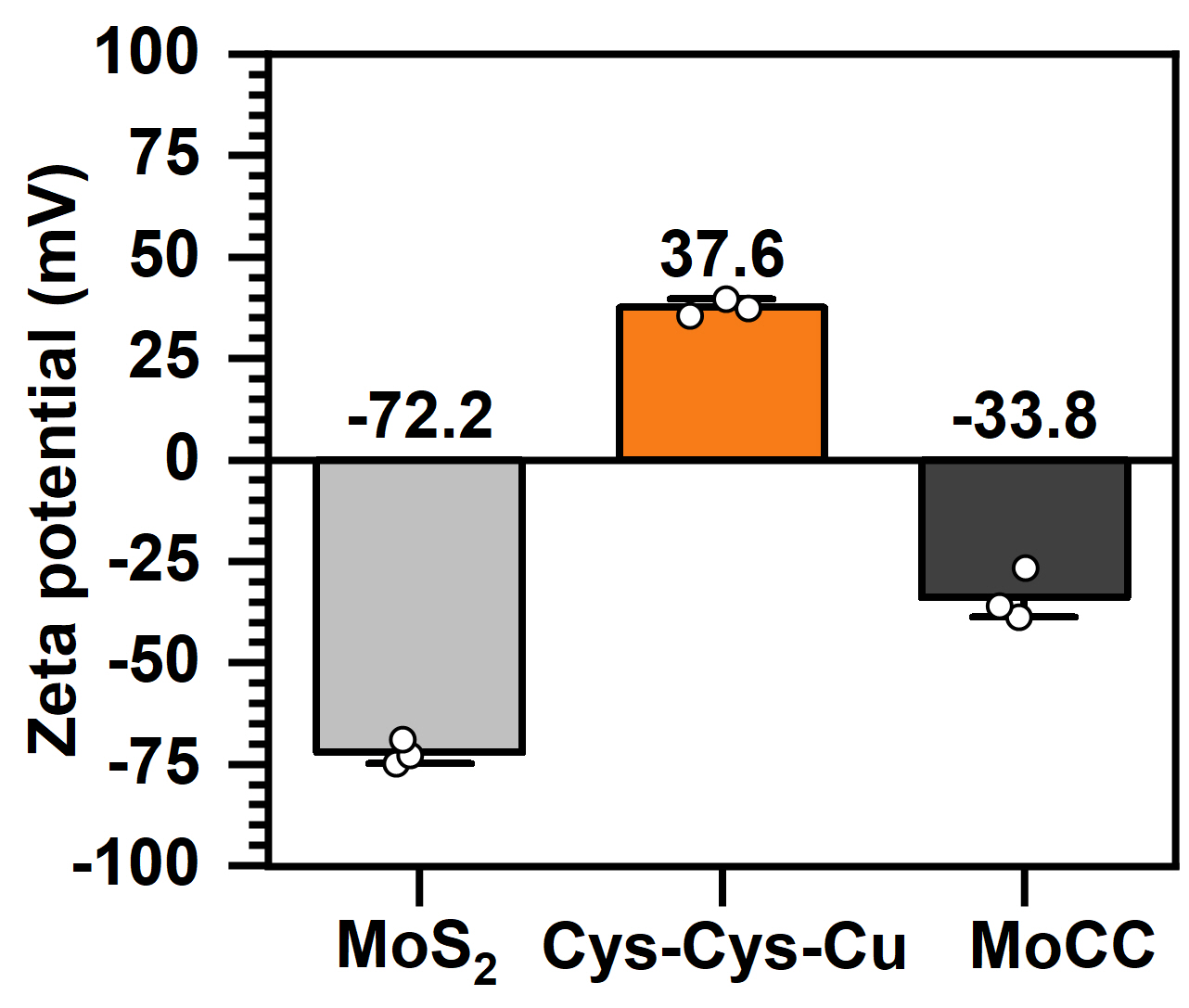


**Figure S7.** Zeta potential of MoS_2_, Cys-Cu-Cys, and MoCC.


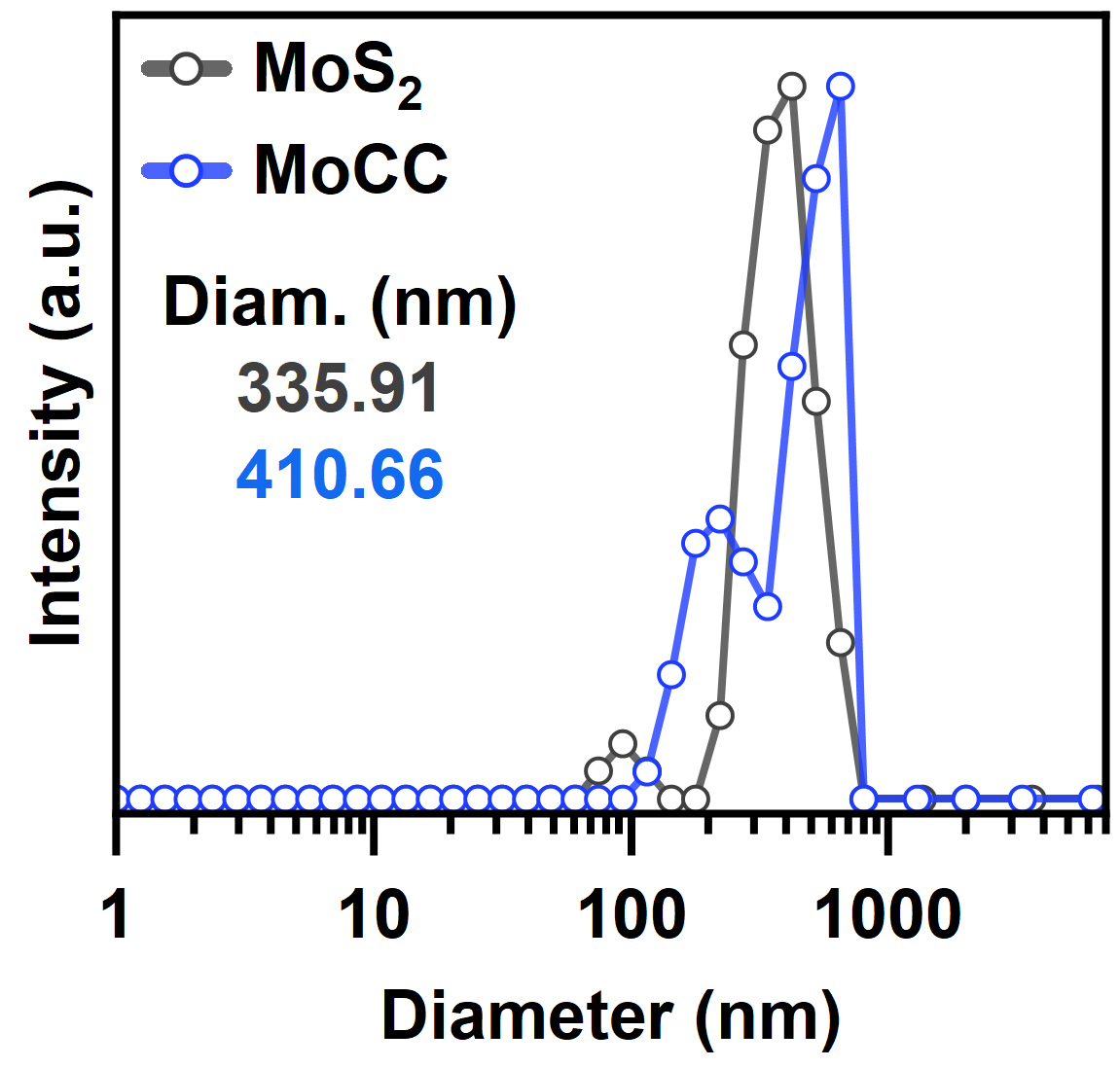


**Figure S8.** Dynamic light scattering (DLS) distribution of MoS_2_ and MoCC.

**
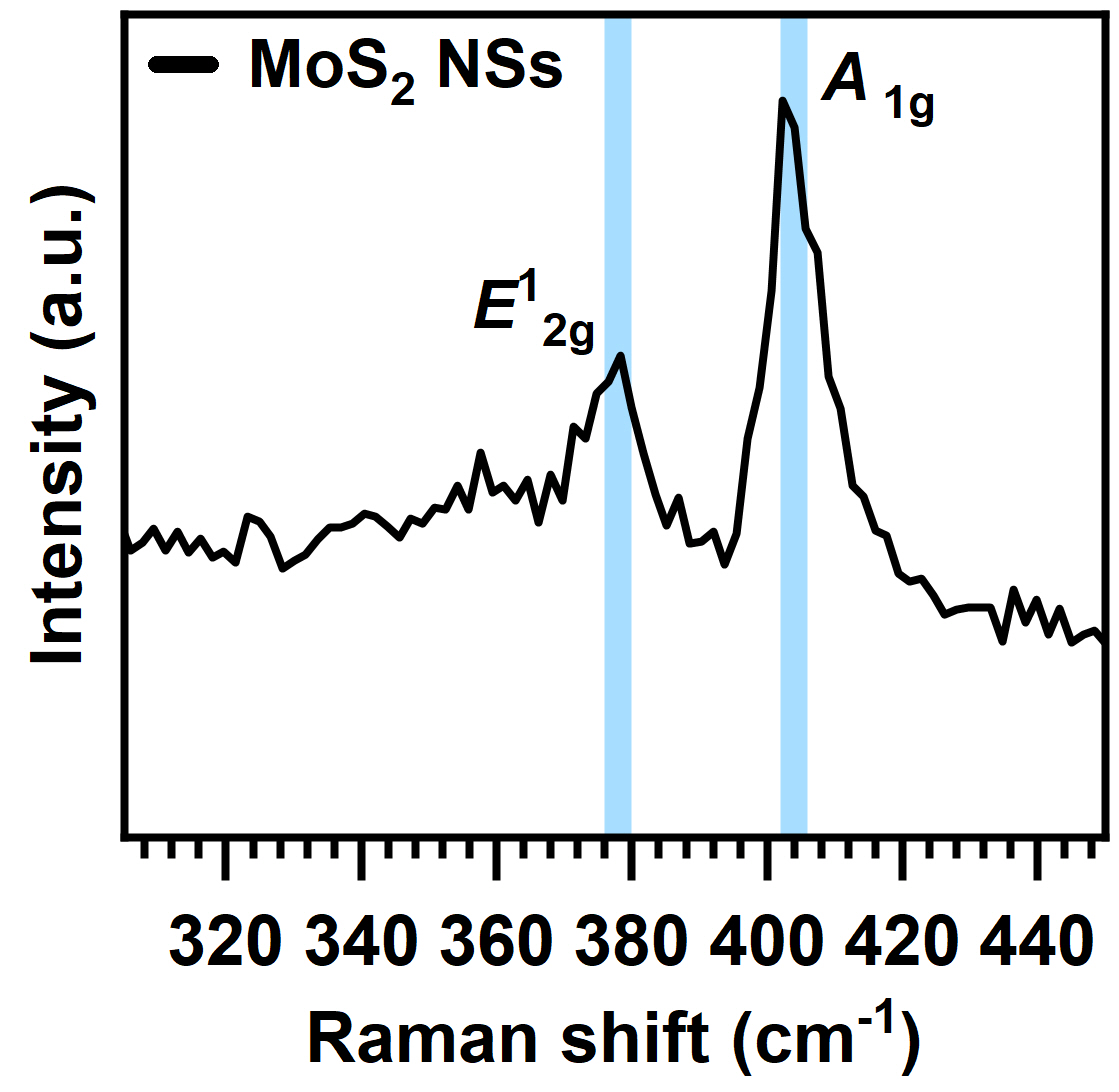
**

**Figure S9.** Raman spectrum of MoS_2_.


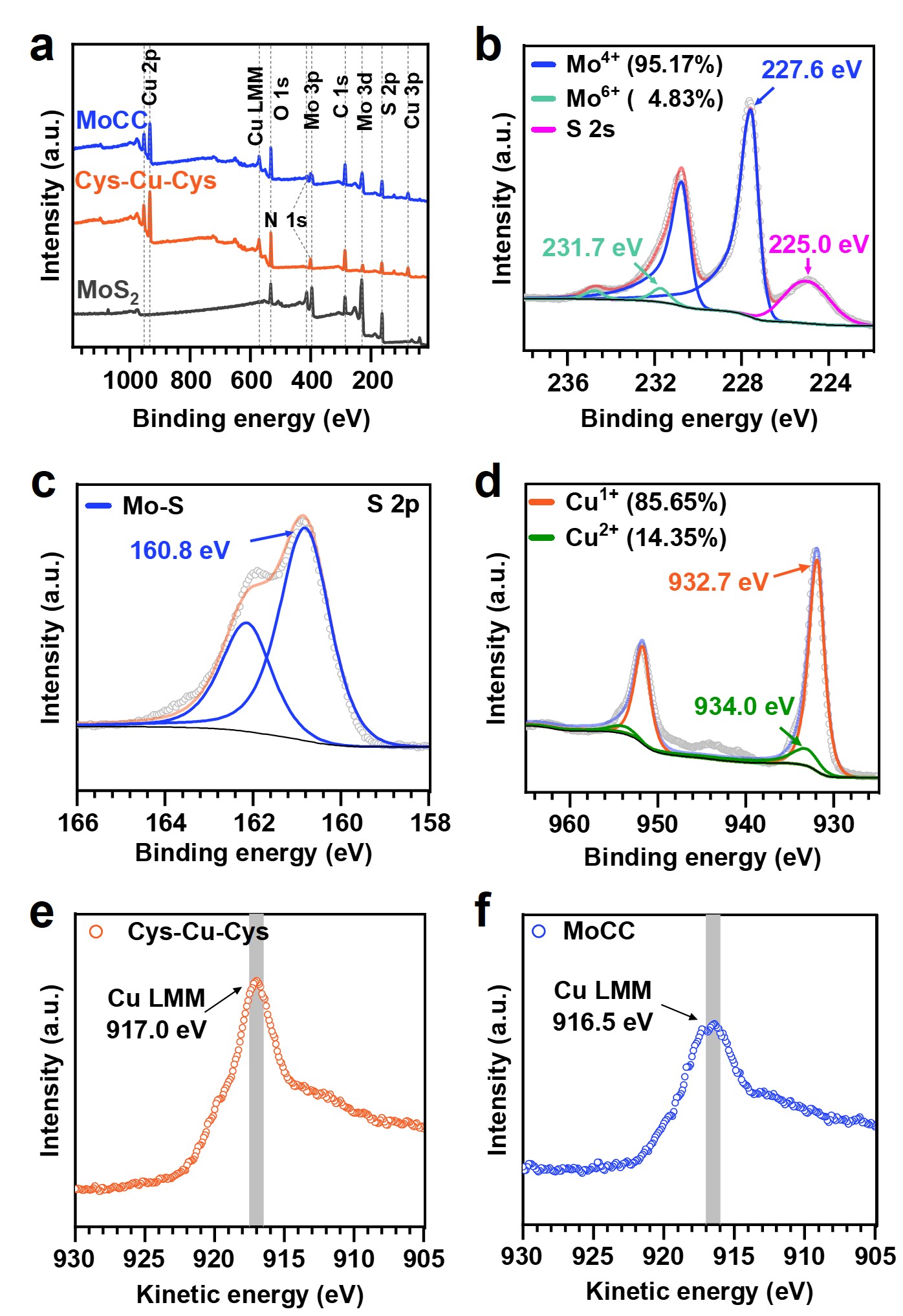


**Figure S10.** (a) XPS spectra of MoS_2_, Cys-Cu-Cys, and MoCC. High resolution XPS spectra of (b) Mo *3d* and (c) S *2p* of MoS_2_. (d) Cu *2p* of Cys-Cu-Cys. Auger Cu LMM spectra of (e) Cys-Cu-Cys and (f) MoCC.


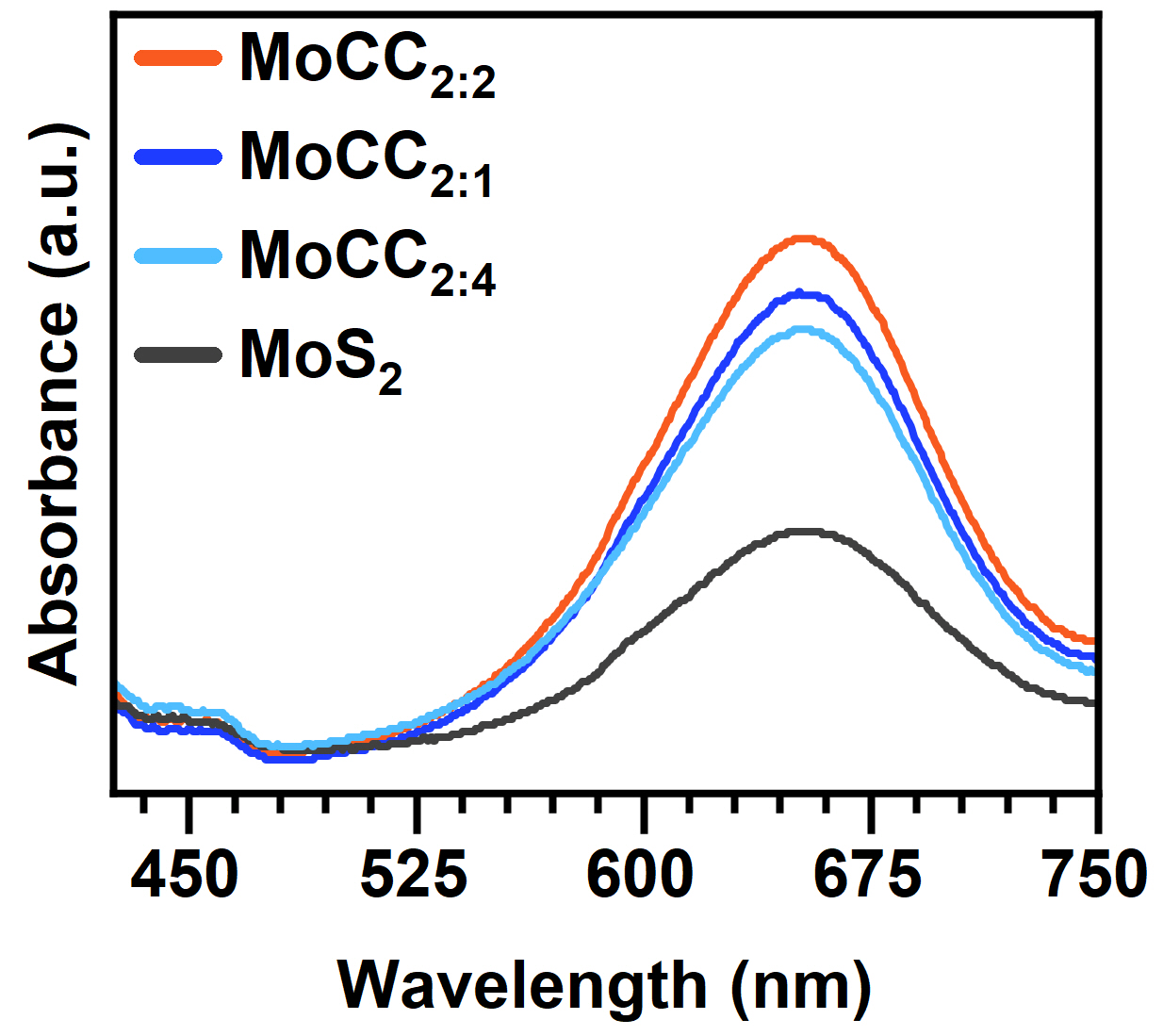


**Figure S11.** Comparison of POD-like activity of MoS_2_ and MoCC with various Cu loading ratios.


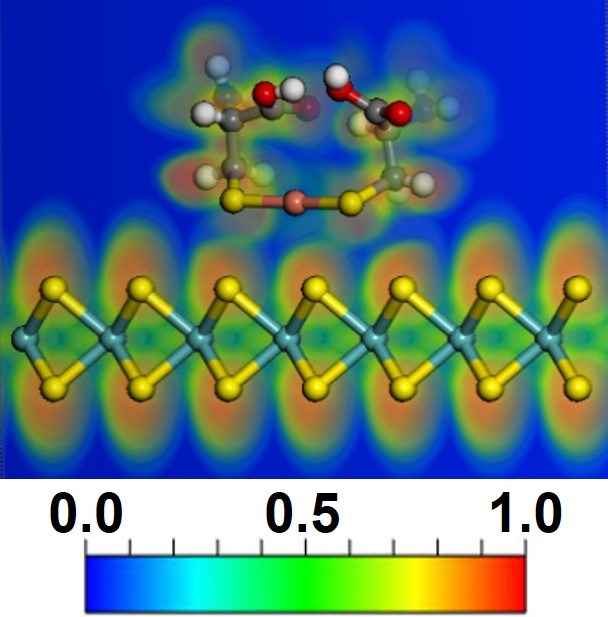


**Figure S12.** The calculated Electron Localization Functions (ELF) of MoCC (n=0-1).


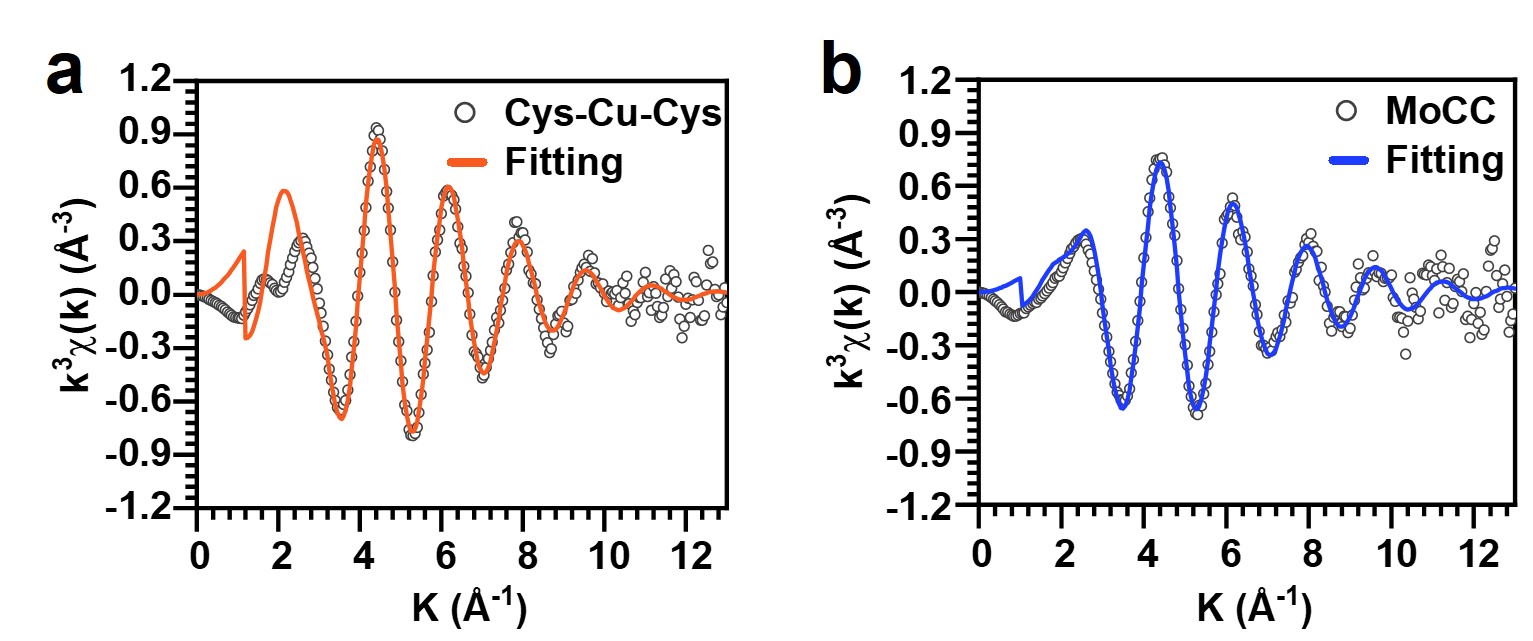


**Figure S13.** K space fitting curve at Cu K-edge of (a) Cys-Cu-Cys and (b) MoCC.
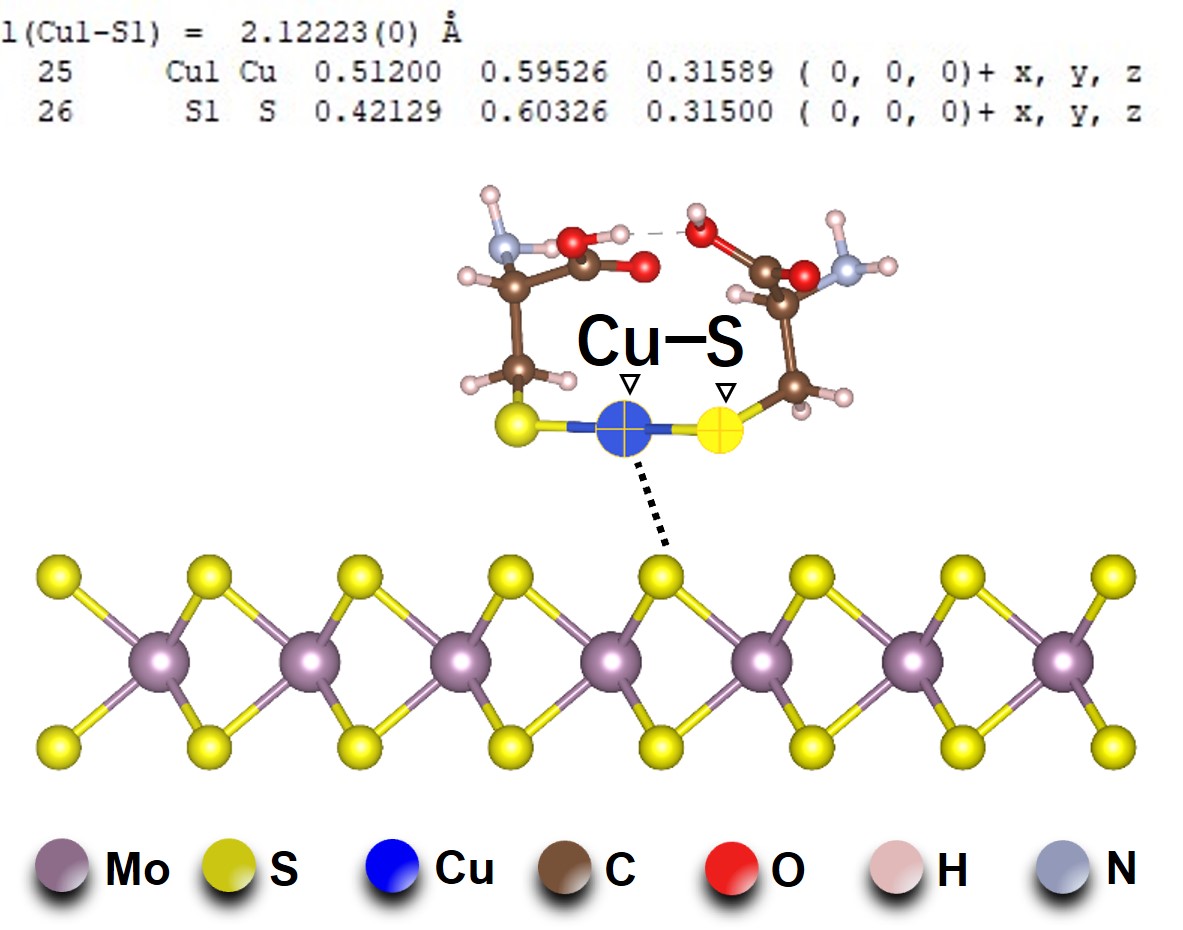


**Figure S14.** The Cu-S bond distance of Cys-Cu-Cys in MoCC predicted by DFT calculations.


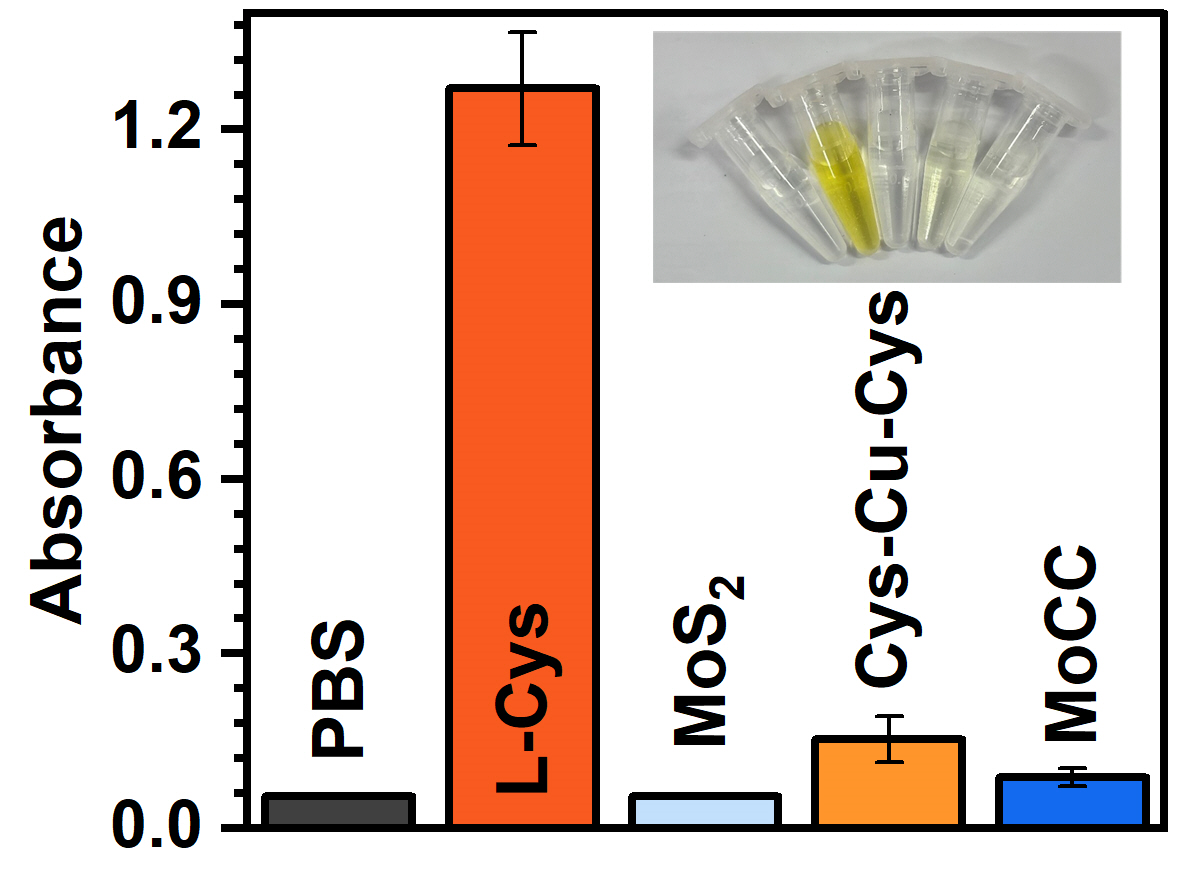


**Figure S15.** Ellman’s assay showing residual free thiol content in each sample, based on absorbance at 412 nm. Insert is the corresponding photograph of samples from left to right of X-axis.


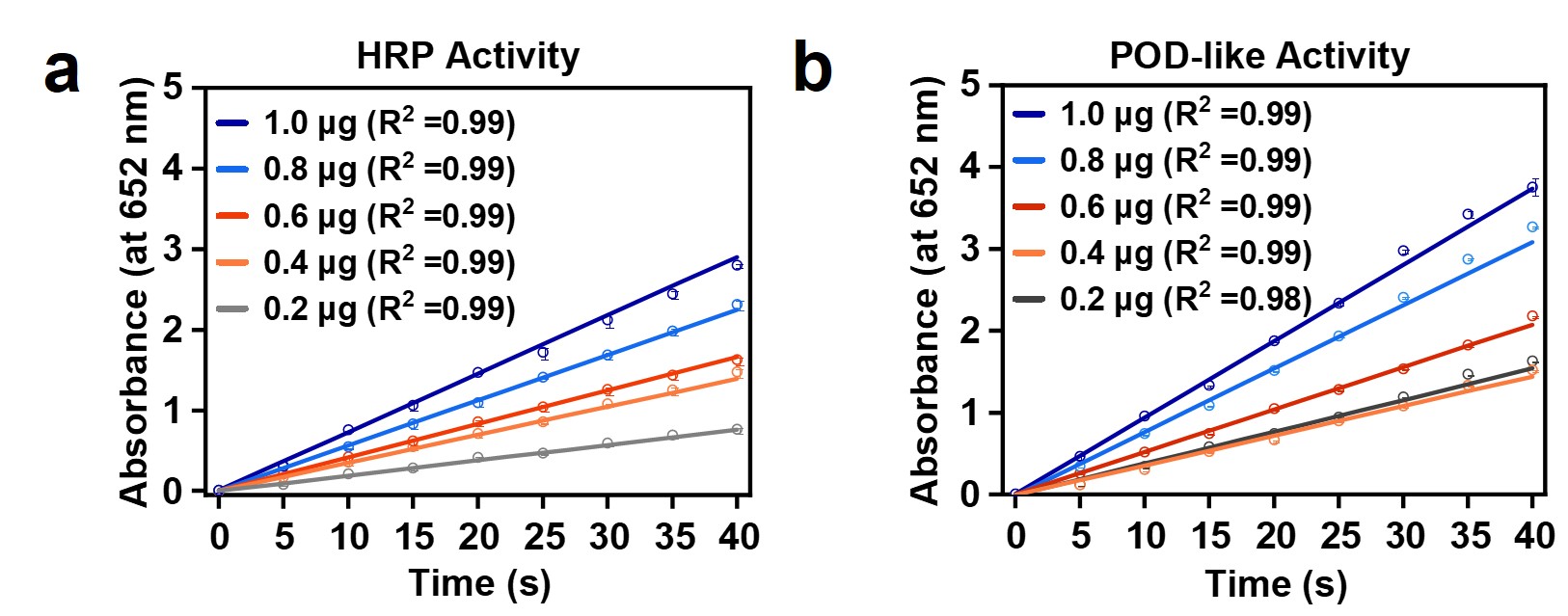


**Figure S16.** The magnified initial linear portion of (a) HRP and (b) MoCC reaction-time curves with corresponding weights. 40 s was chosen for the initial rate period.


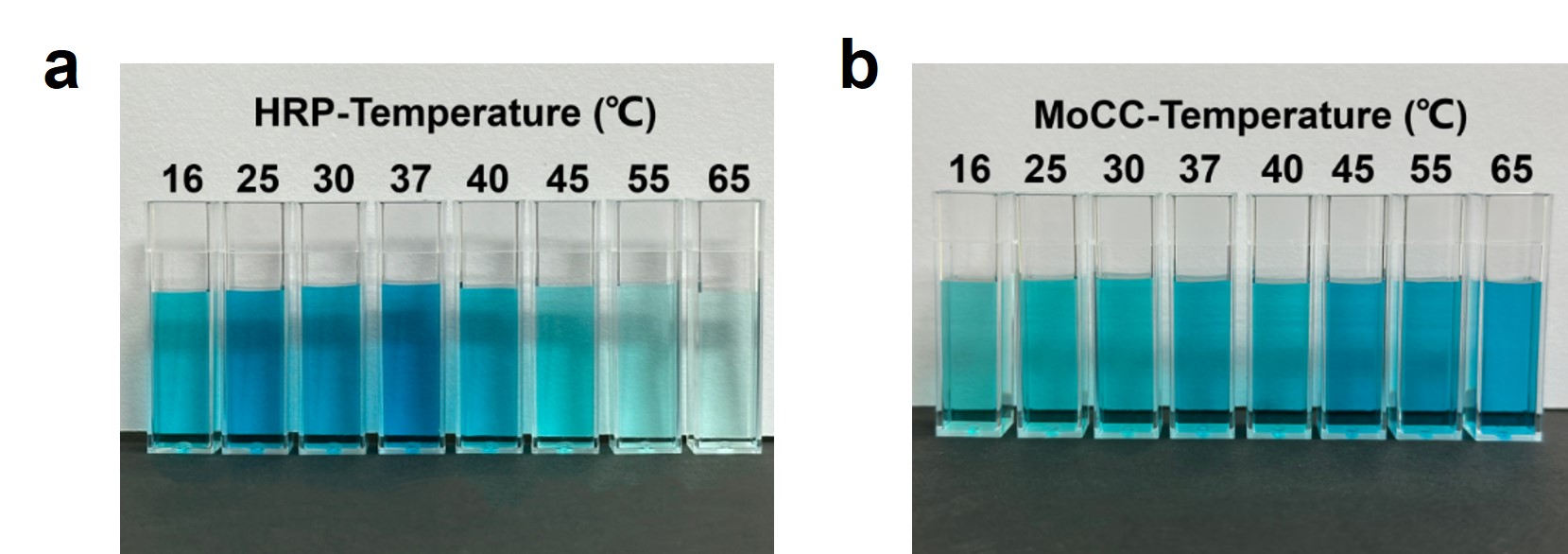


**Figure S17.** The color change of temperature-dependent POD-like catalytic activities of (a) HRP and (b) MoCC corresponding to Figure 3b.


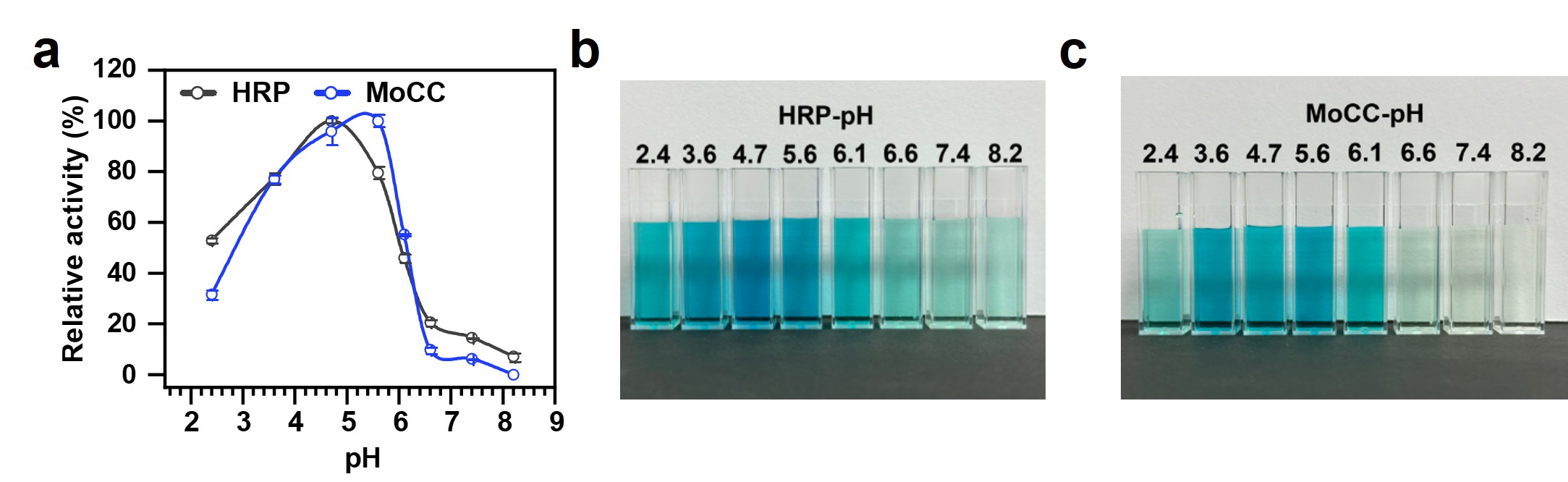


**Figure S18.** (a) Comparison of pH-dependent POD-like activities of HRP and MoCC. The color changes of pH-dependent POD-like activity of (b) HRP and (c) MoCC corresponding to (a).


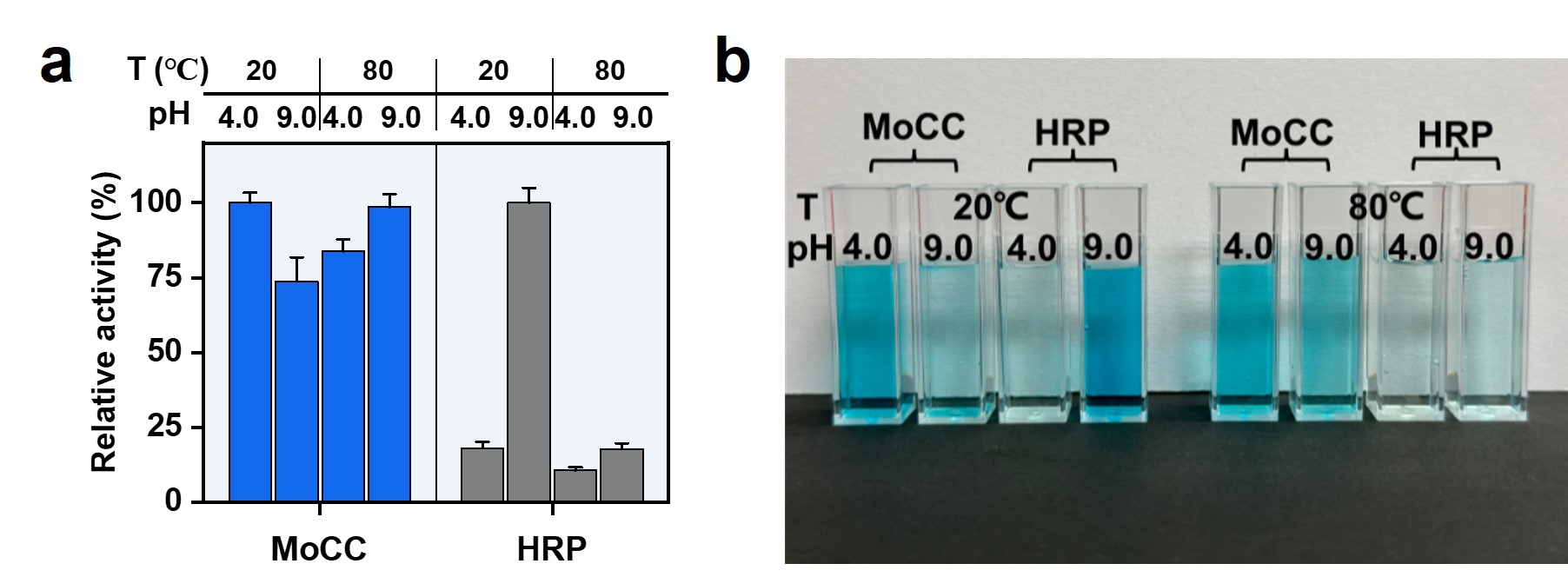


**Figure S19. (**a) Comparison of POD-like activities of HRP and MoCC after undergoing acid-alkaline store conditions of pH 4.0 and 9.0 at 20°C and 80°C for 24 h. (b) The corresponding color changes in (a).


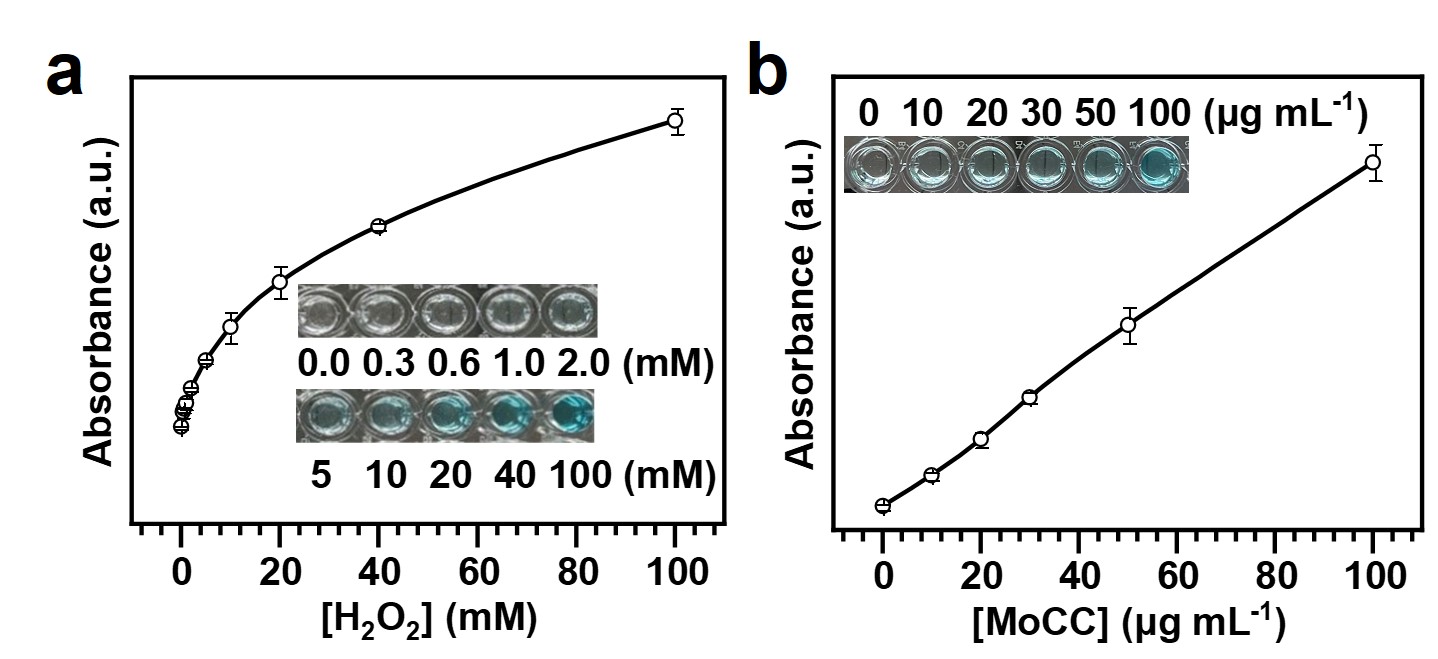


**Figure S20.** (a) H_2_O_2_ and (b) MoCC concentration-dependent POD-like activity. Insets showed the corresponding color changes.

**
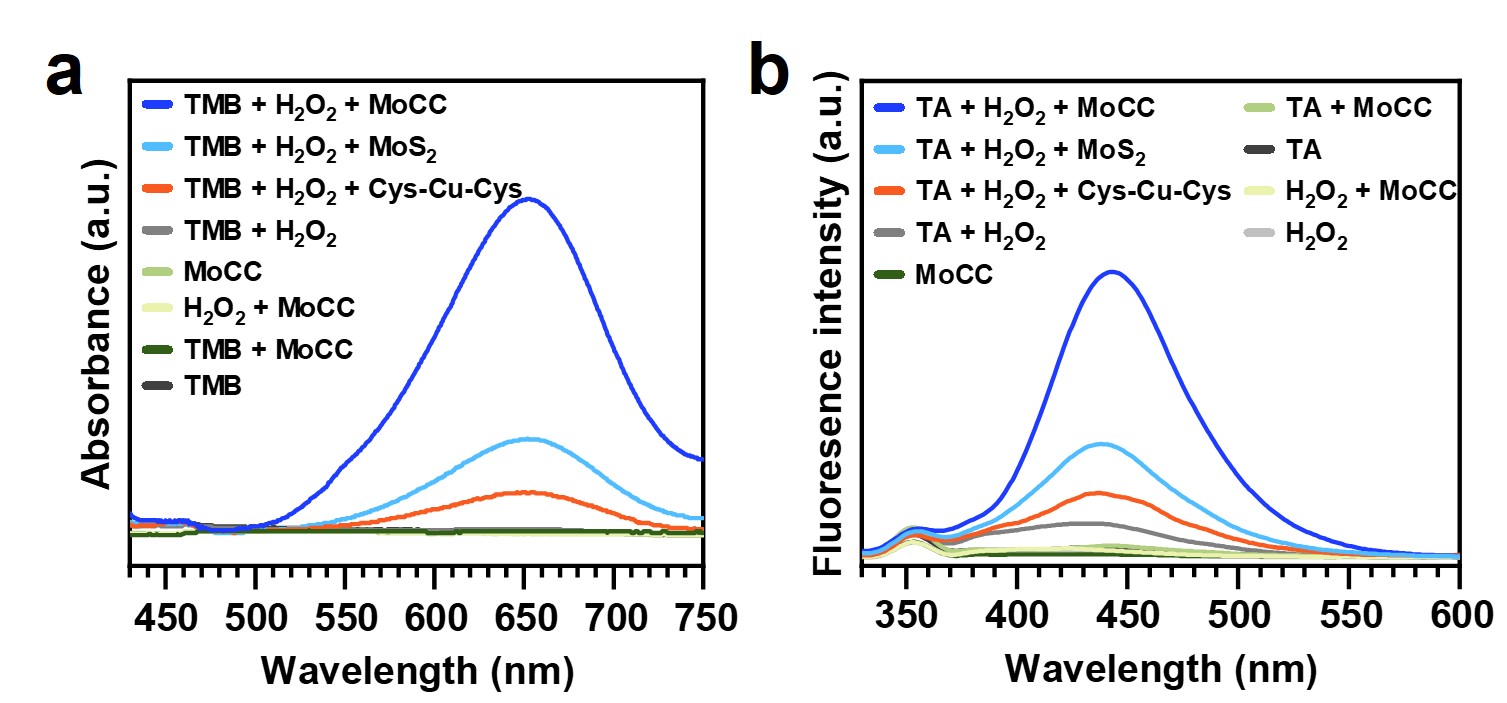
**

**Figure S21.** •OH detection by (a) TMB and (b) TA under different treatments.

**
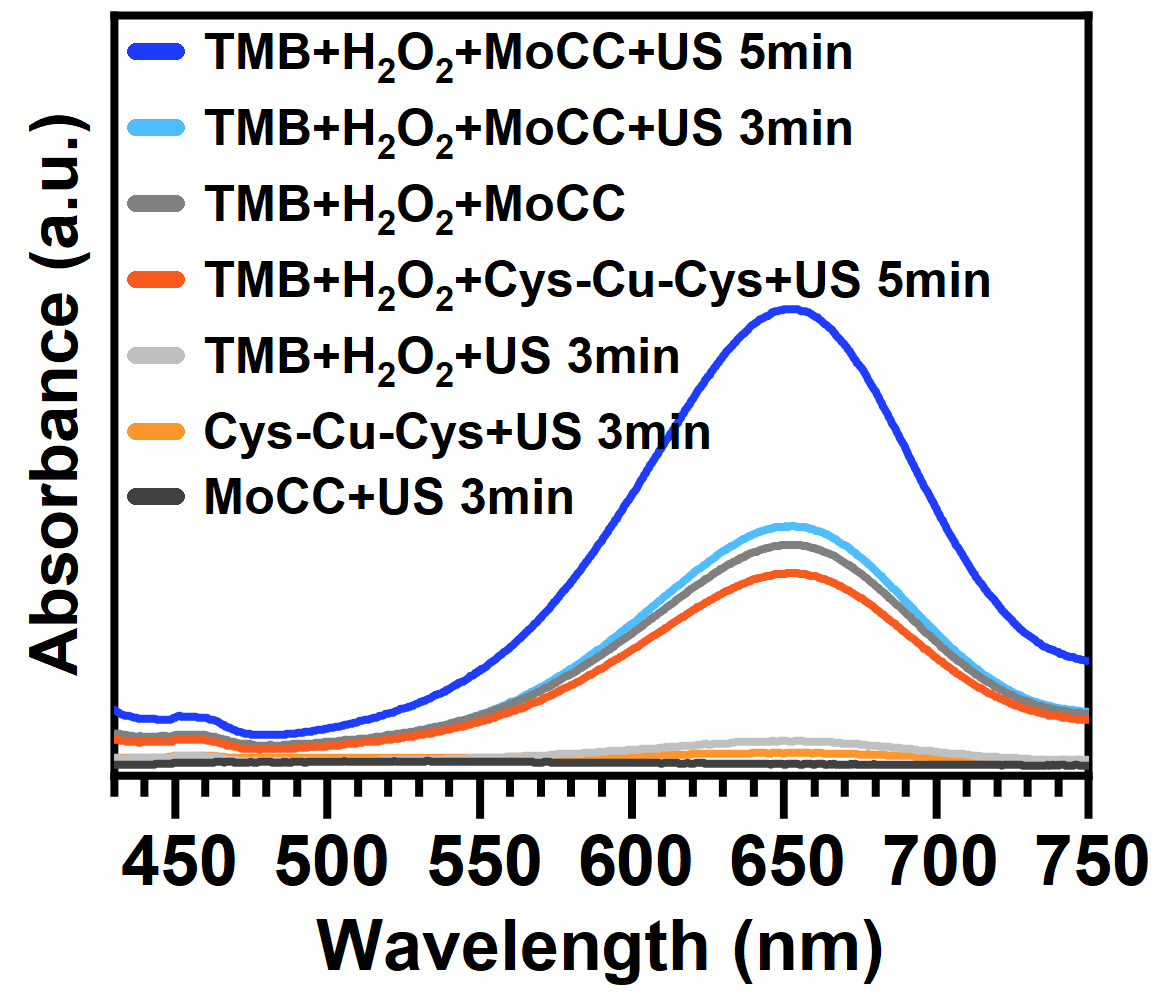
**

**Figure S22.** •OH detection using TMB colorimetric method under different US conditions.

**
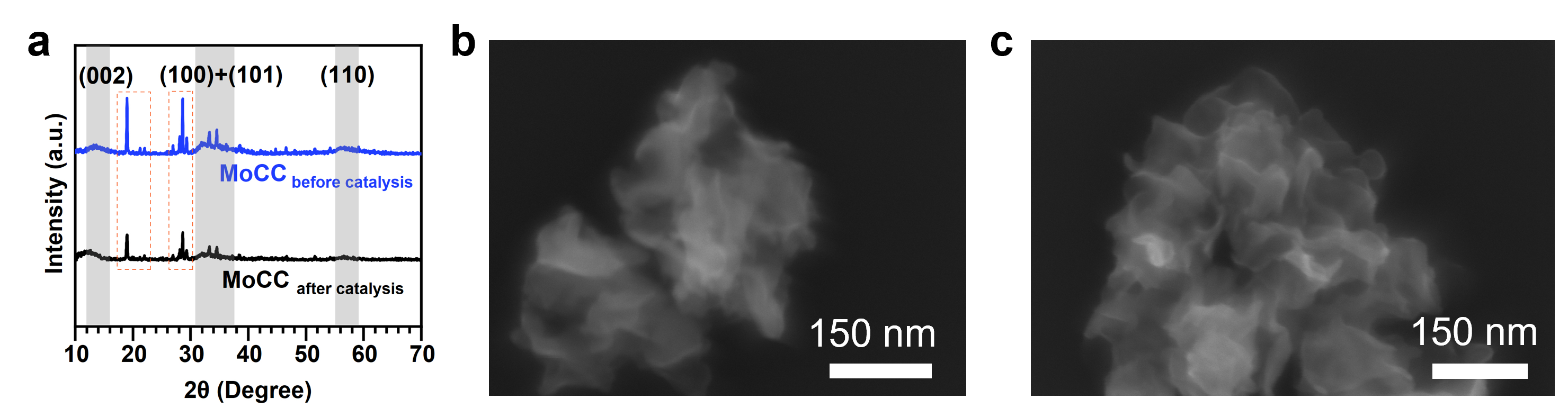
**

**Figure S23.** Structural stability of MoCC before and after catalysis (TMB + H_2_O_2_ + MoCC + US 5 min). (a) XRD patterns of MoCC. SEM images of MoCC (b) before and (c) after catalysis.

**
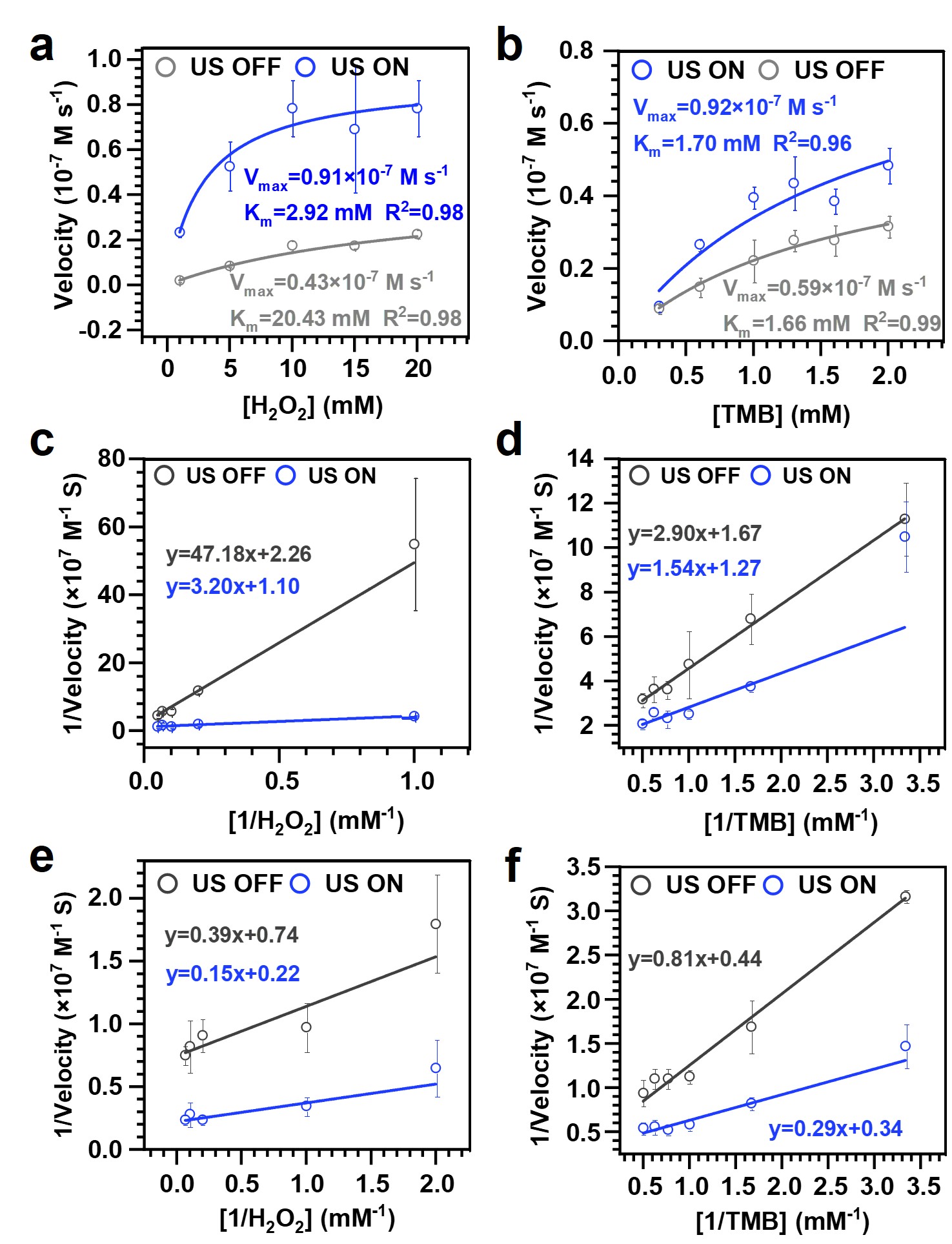
**

**Figure S24.** Characterization of the POD-like kinetics under different conditions. Michaelis–Menten fitted curves for MoS_2_ with (a) H_2_O_2_ and (b) TMB as substrates, and corresponding (c, d) Lineweaver–Burk fitted curves. (e, f) Lineweaver–Burk fitted curves for MoCC, corresponding to Figure 3g, h.

**
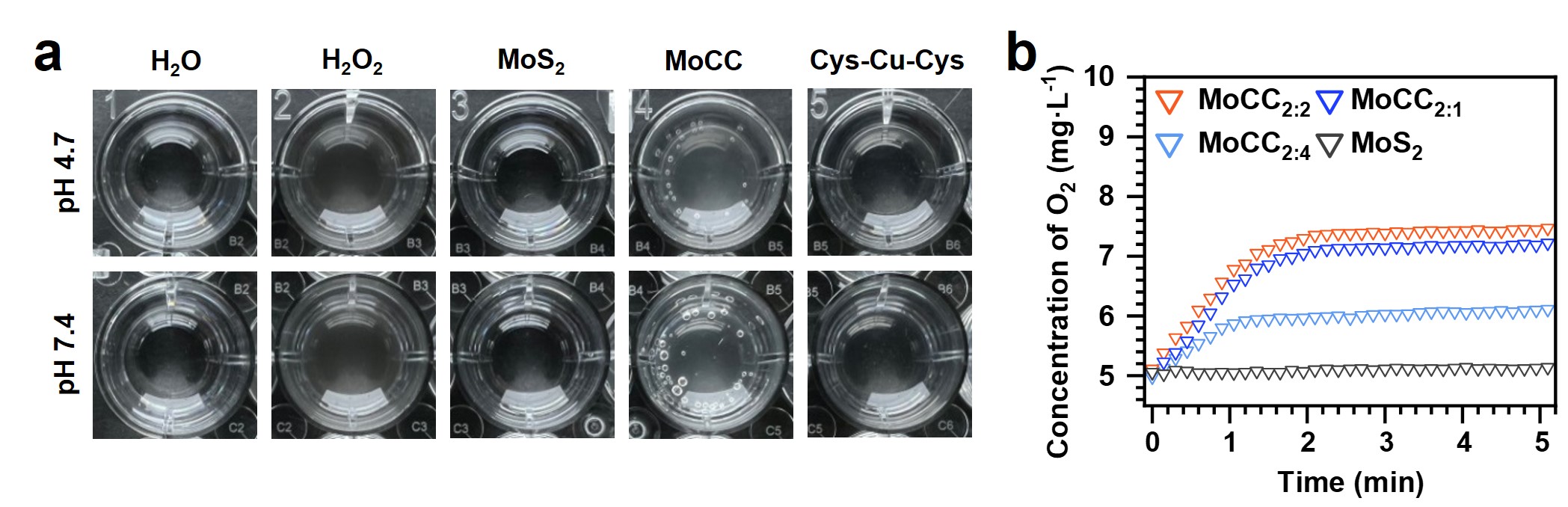
**

**Figure S25.** (a) Photos of CAT-like activity with different pH treatments, (b) CAT-like activity of MoS_2_ and MoCC with different Cu loading ratios.

**
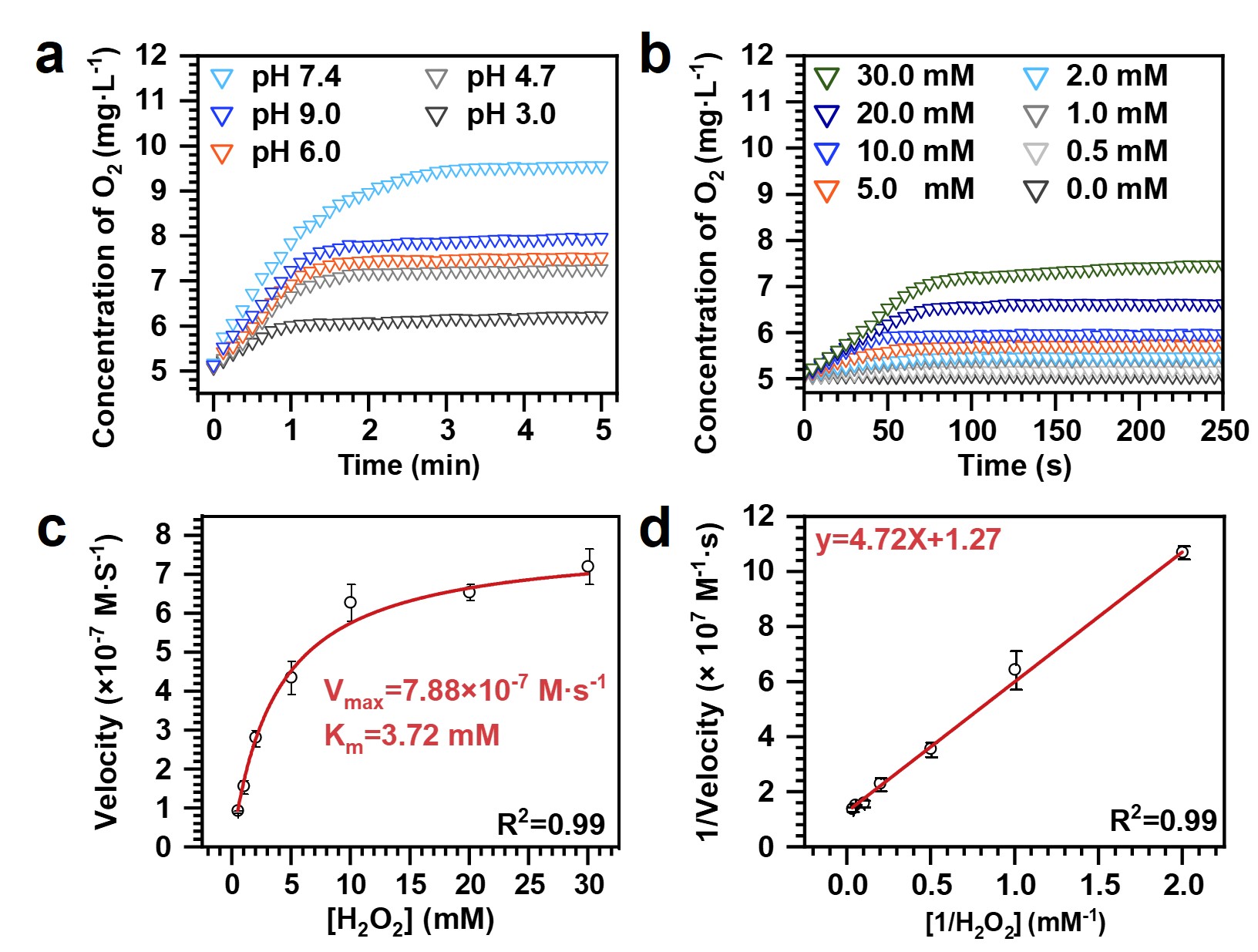
**

**Figure S26.** Reaction-time curves of CAT-like activity under different (a) pH and (b) H_2_O_2_ concentrations; (c) Michaelis-Menten and (d) Lineweaver–Burk fittings for the CAT-like activities of MoCC at different H_2_O_2_ concentrations.

**
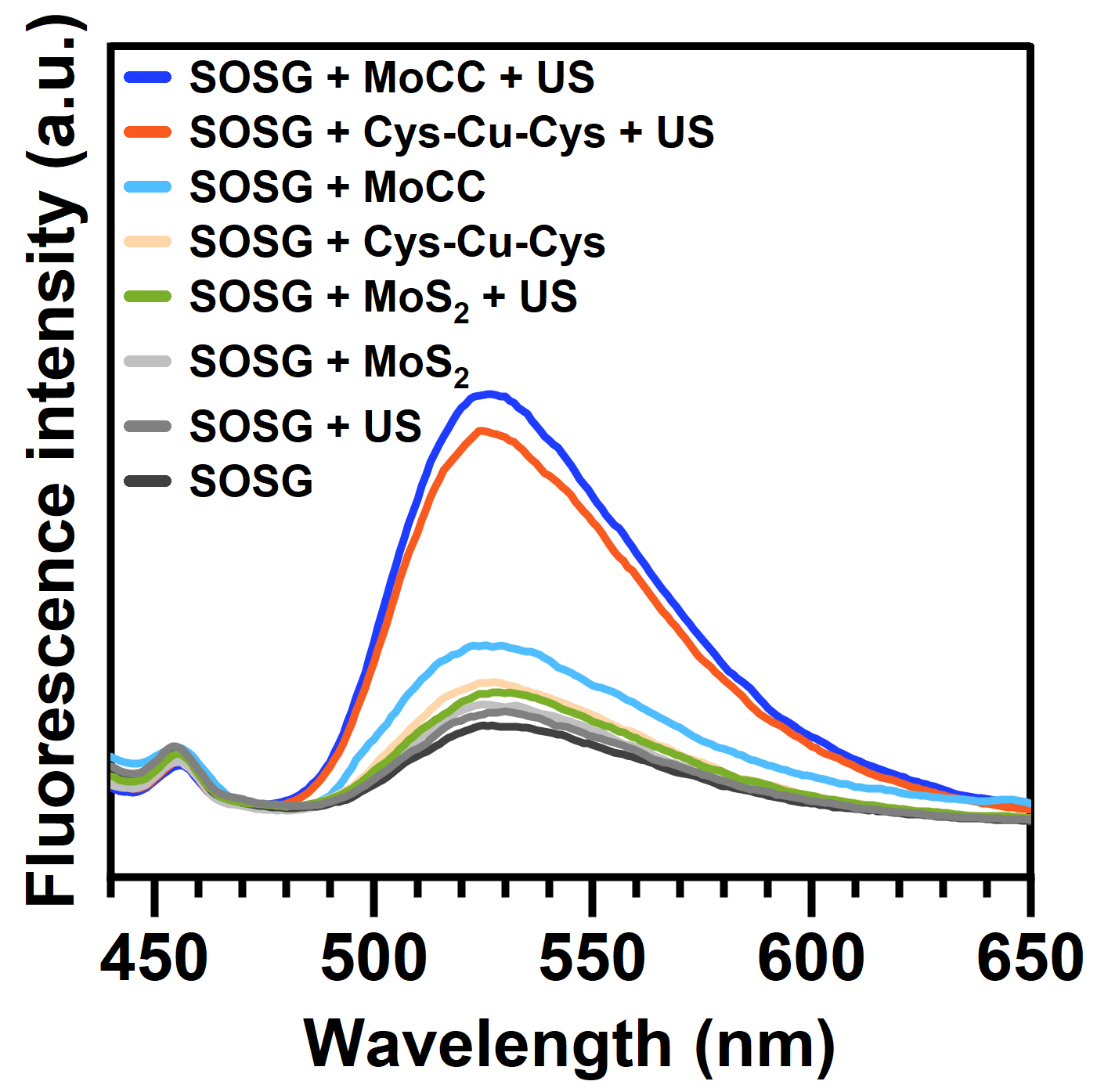
**

**Figure S27.** ^1^O_2_ detection using SOSG probe under different treatments.

**
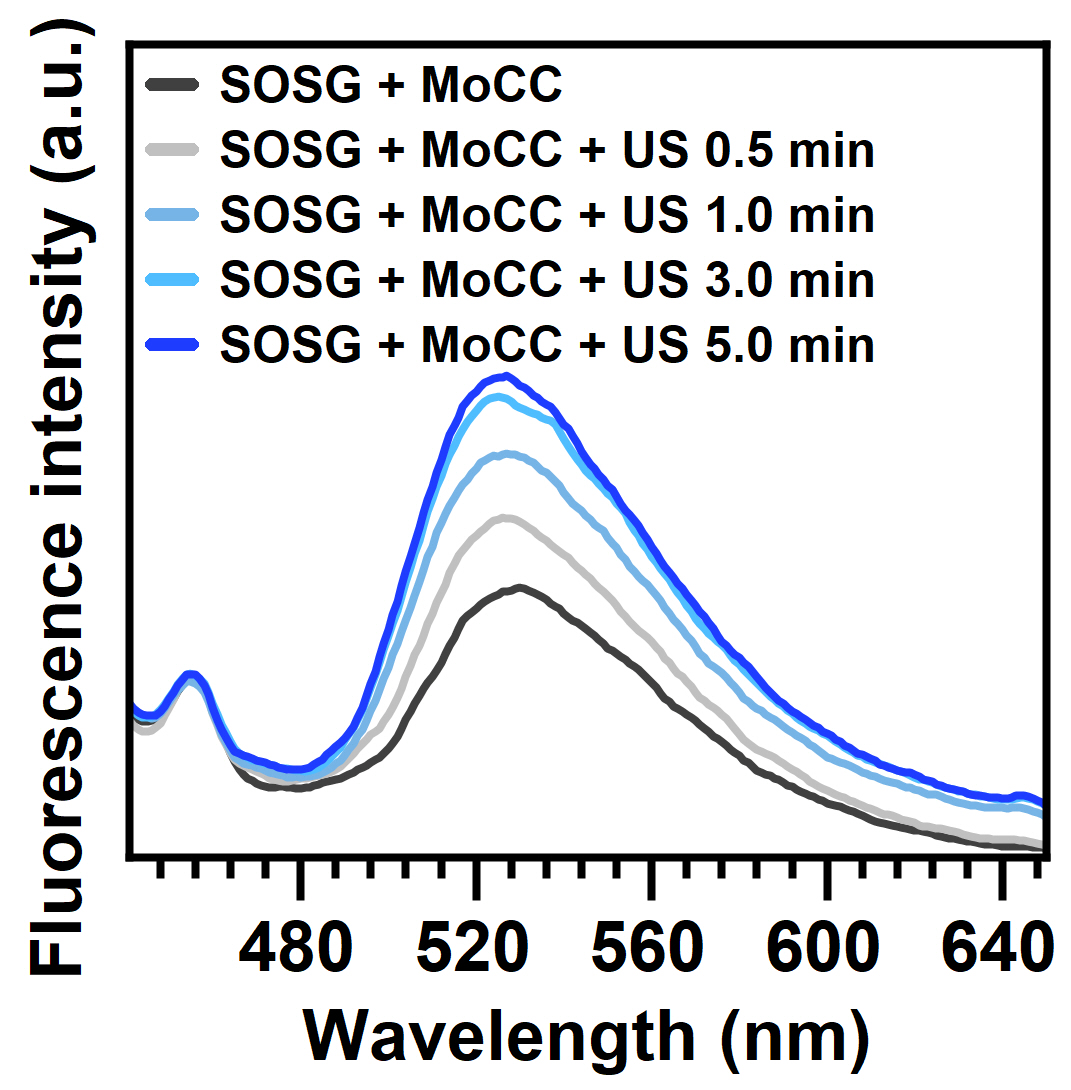
**

**Figure S28.** ^1^O_2_ detection using SOSG probe under different US conditions.


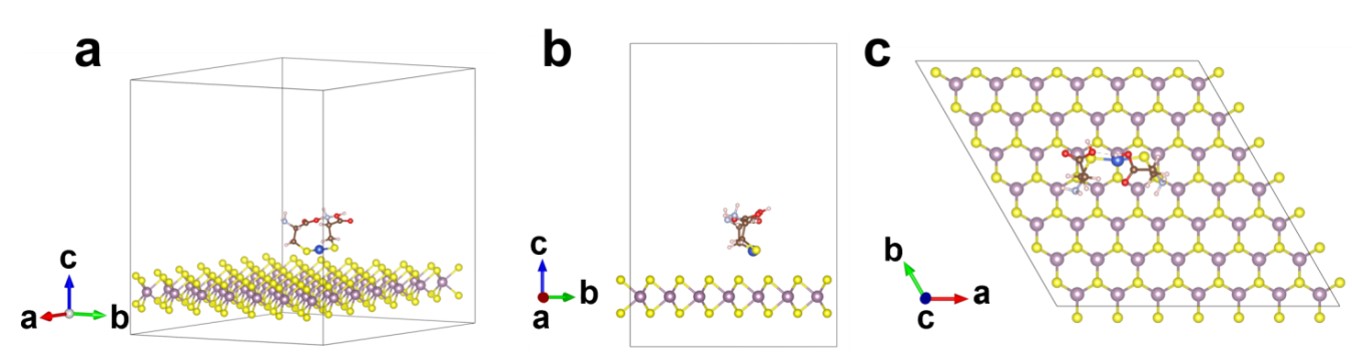


**Figure S29.** The optimized theoretical calculation models of MoCC (a) 3D view, (b) side view (c) top view. Mo, dark purple; S, yellow; Cu, blue; C, brown; O, red; H, pink; N, gray.


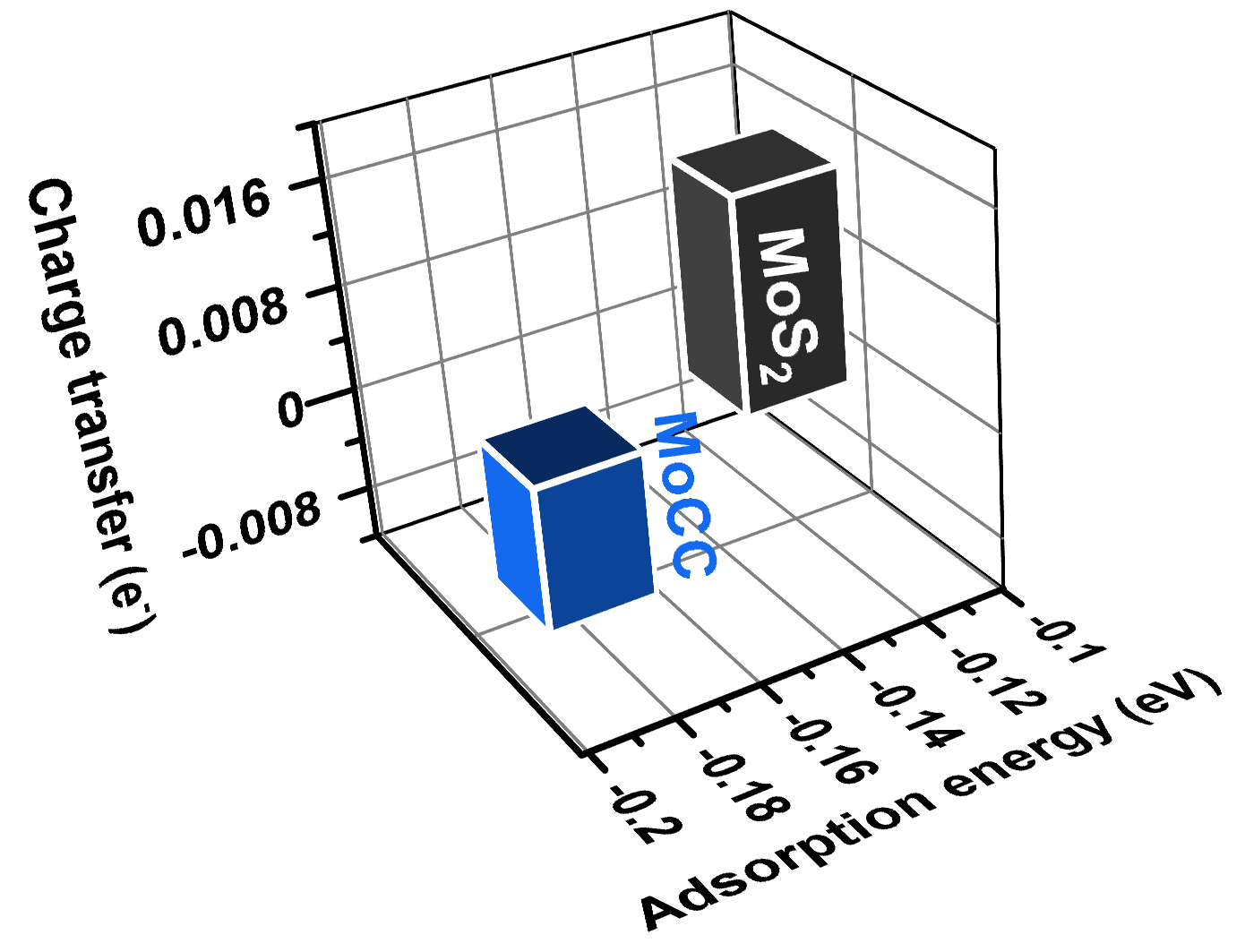


**Figure S30.** Calculated H_2_O_2_* adsorption energy and Bader Charge values on MoS_2_ and MoCC.


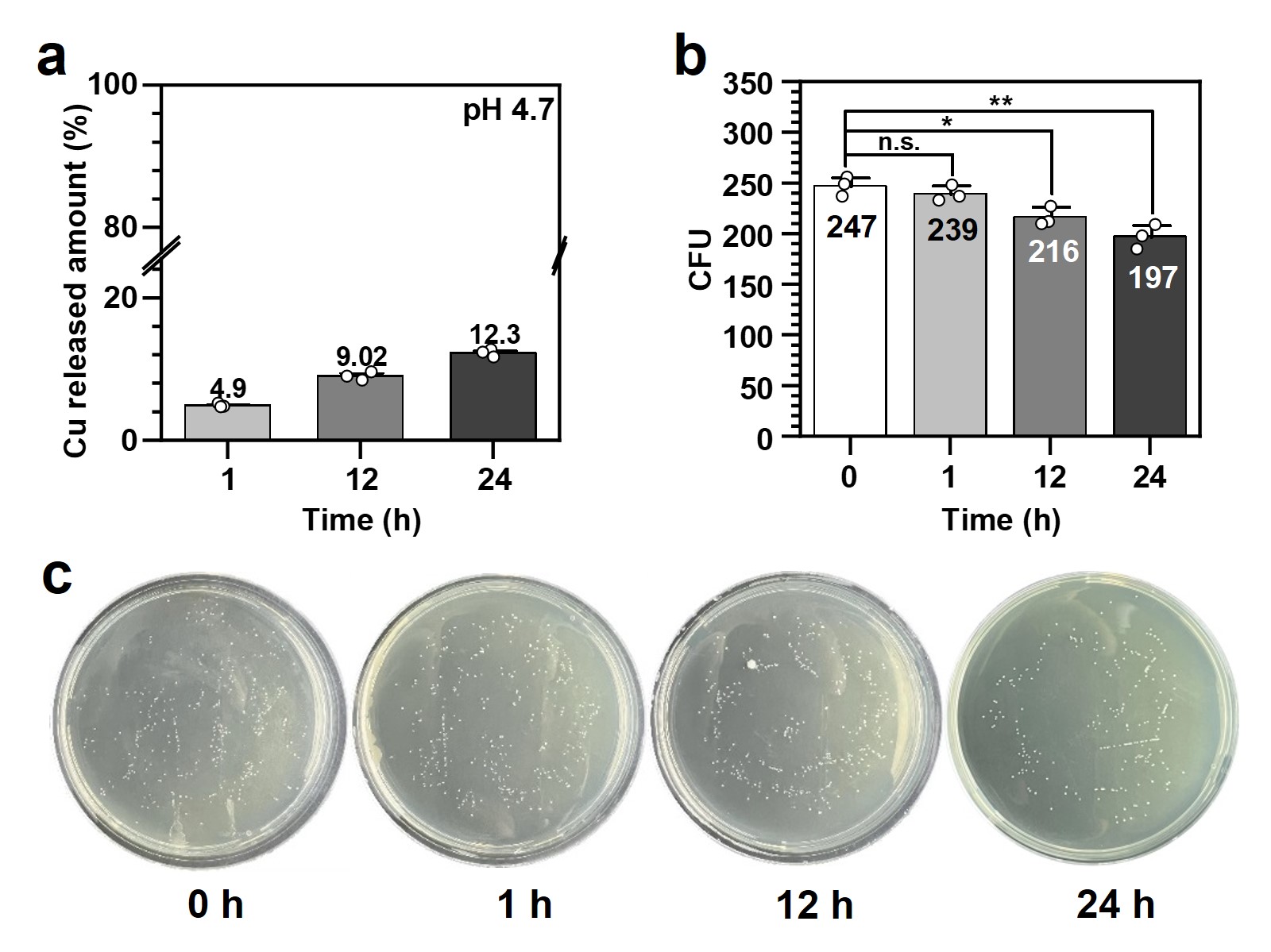


**Figure S31.** (a) Amount of Cu ions released from MoCC. (b) Antibacterial efficiency of released Cu ions and (c) corresponding photos of bacterial colonies. P values were calculated by one-way ANOVA followed by Tukey’s multiple comparison test. * and ** indicate p < 0.05 and p < 0.01, respectively.


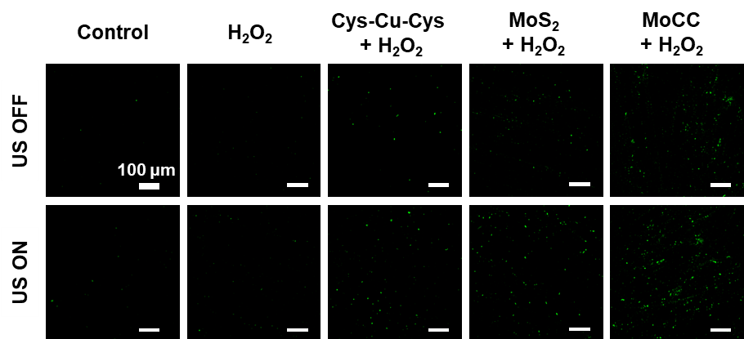


**Figure S32.** Fluorescence images of bacterial cells using DCFH-DA probe after various treatments for ROS detection. [H_2_O_2_]: 150 μM.


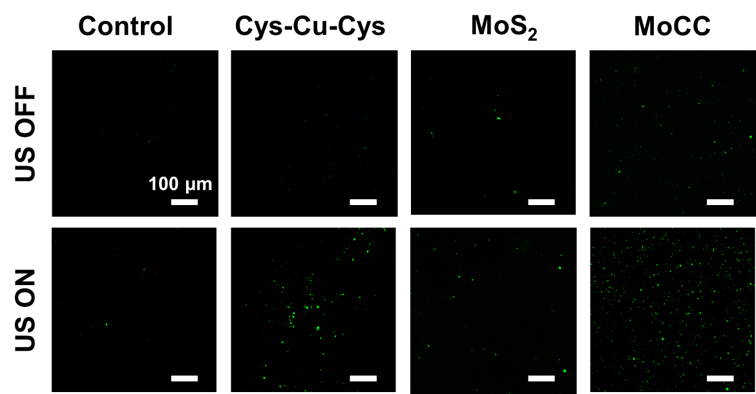


**Figure S33.** Fluorescent signals of ^1^O_2_ using SOSG probe after different treatments.


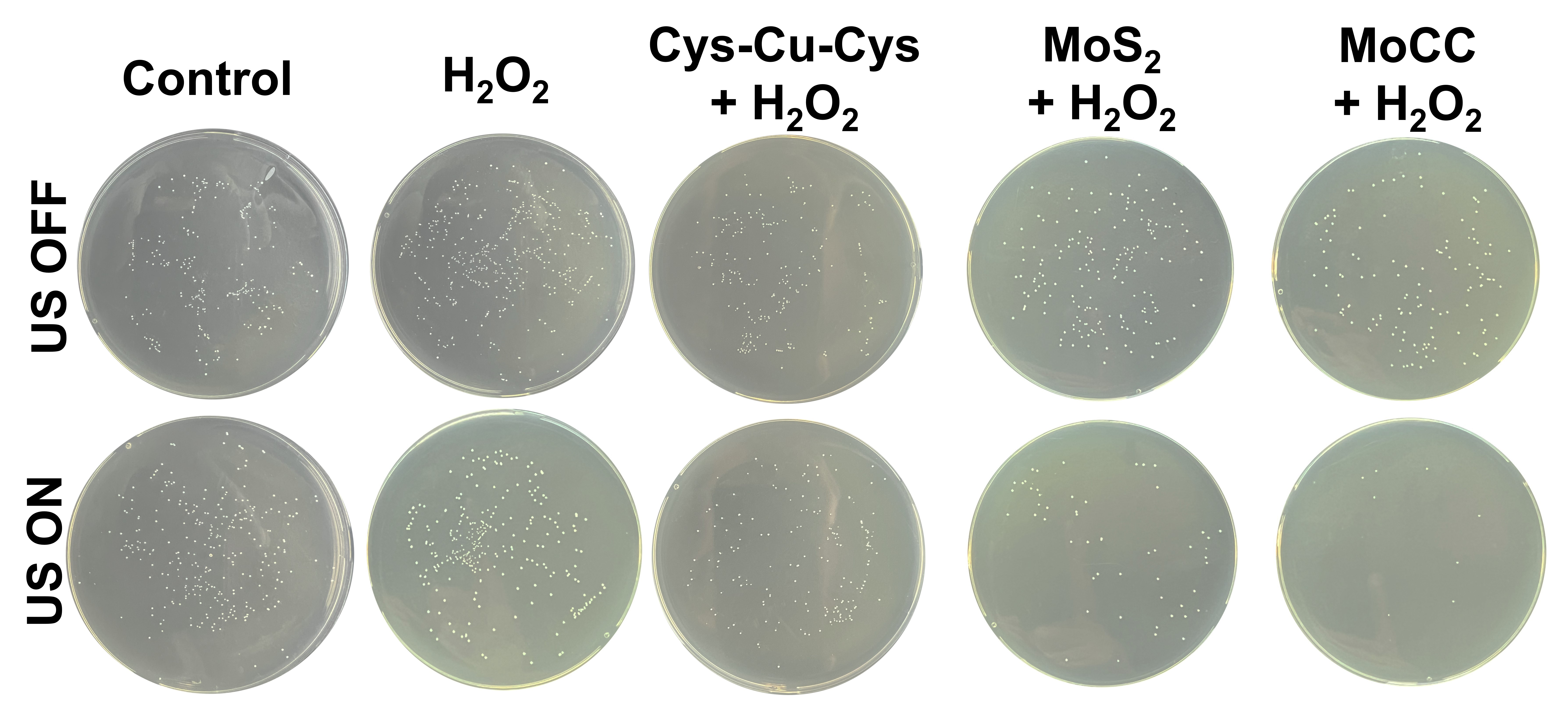


**Figure S34.** Photos of bacterial colonies after different treatments corresponding to Figure 5b.


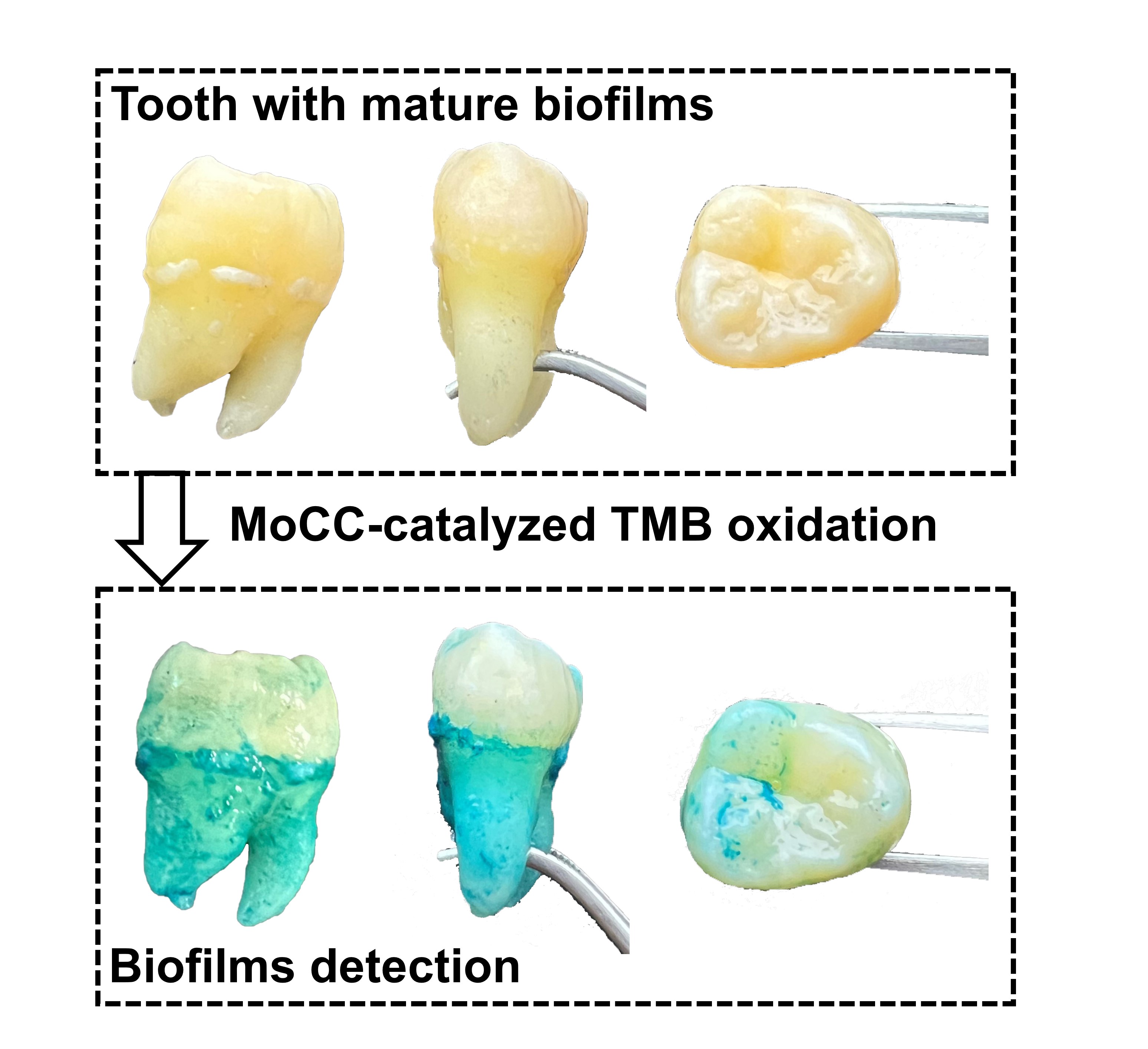


**Figure S35.** MoCC adhered to teeth with mature biofilms, enabling biofilms detection through a visible color changes.


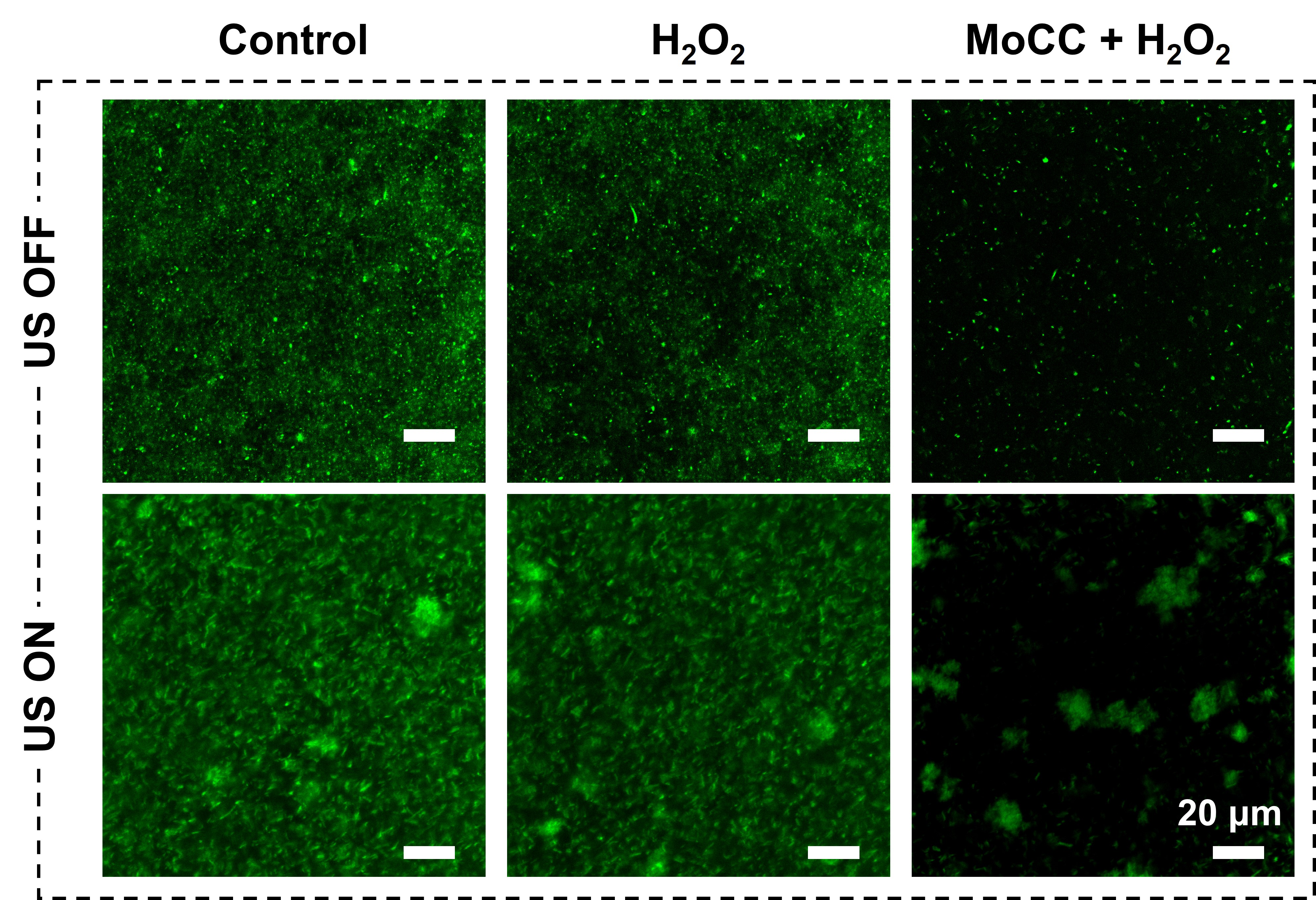


**Figure S36.** Fluorescent signals of EPS in biofilms labeled with FITC-conA after different treatments.


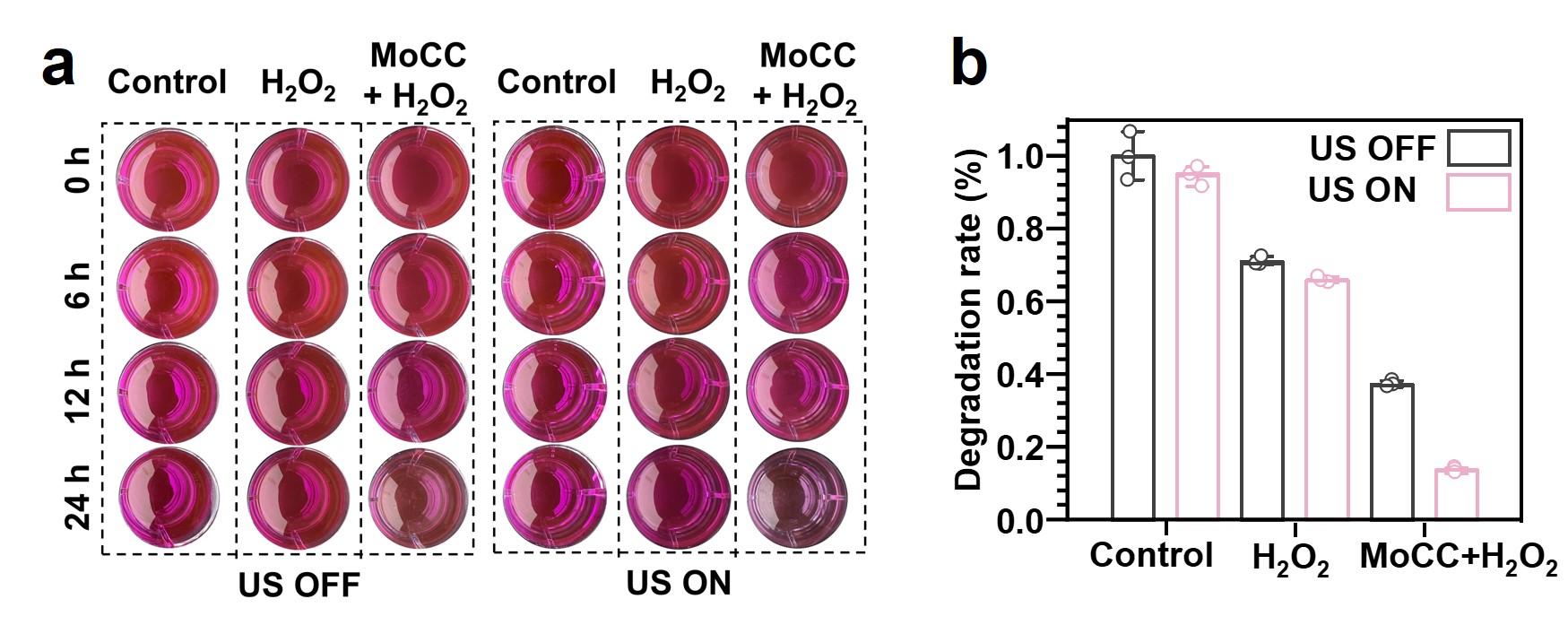


**Figure S37.** (a) Images of RhB degradation after various treatments and (b) corresponding degradation rates.


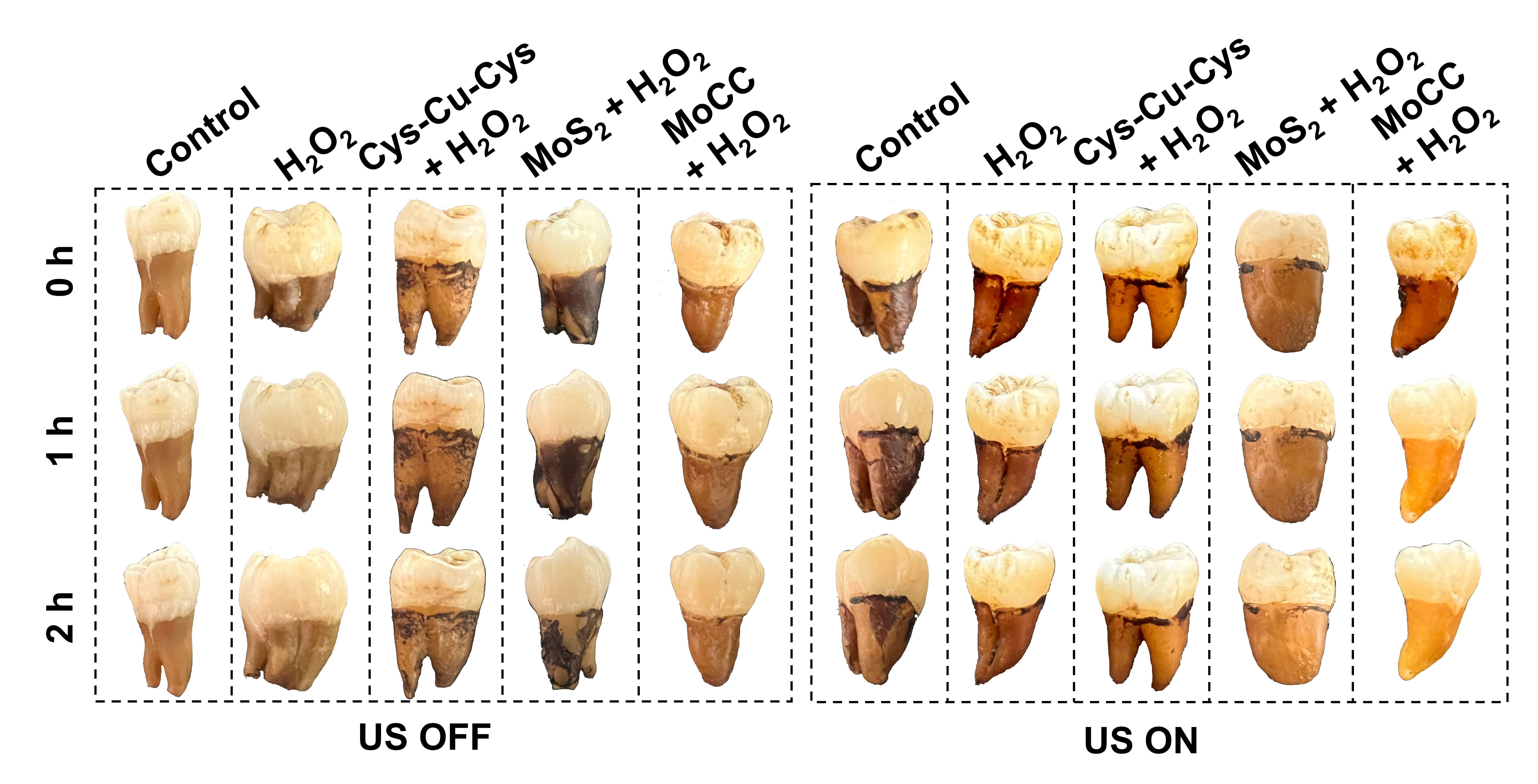


**Figure S38.** Photographs of teeth after various treatments for 0, 1, and 2 h, respectively.


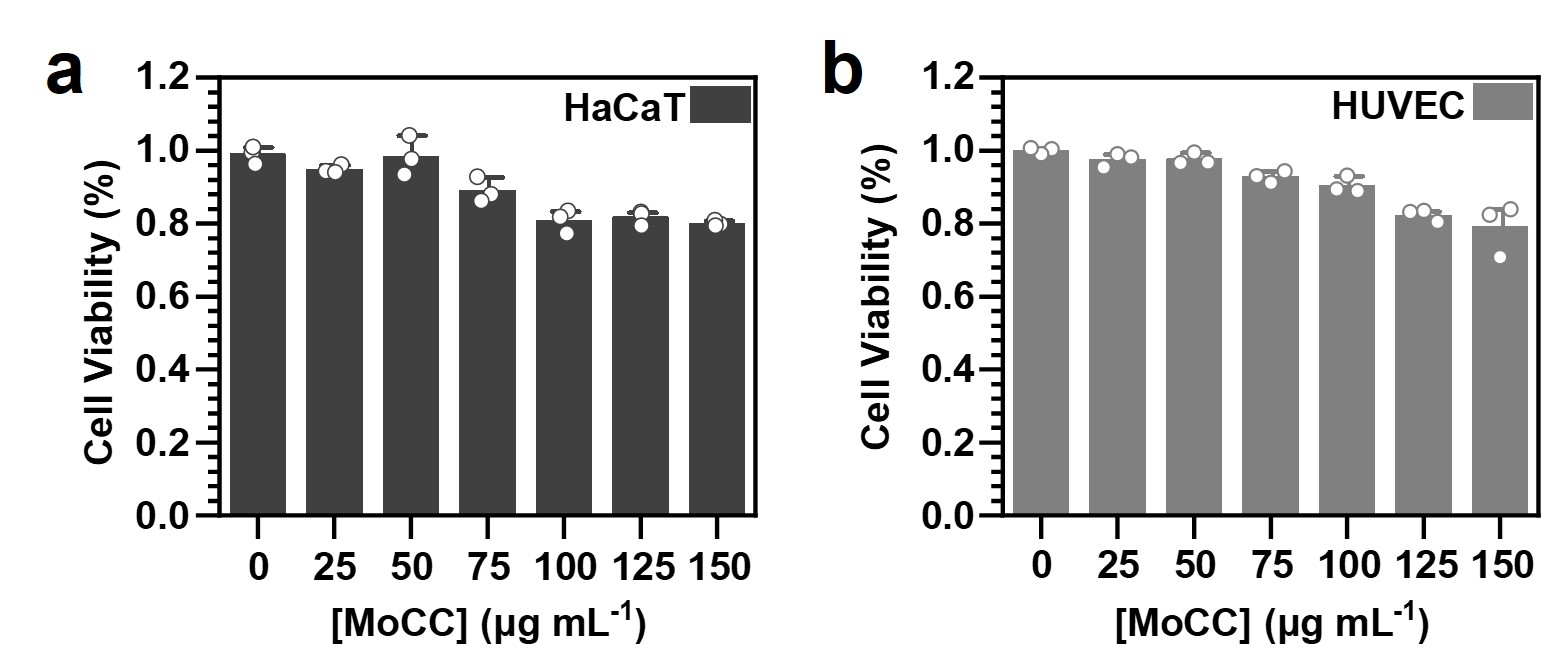


**Figure S39.** (a) HaCaT and (b) HUVEC cell viabilities after 24 h of incubation.


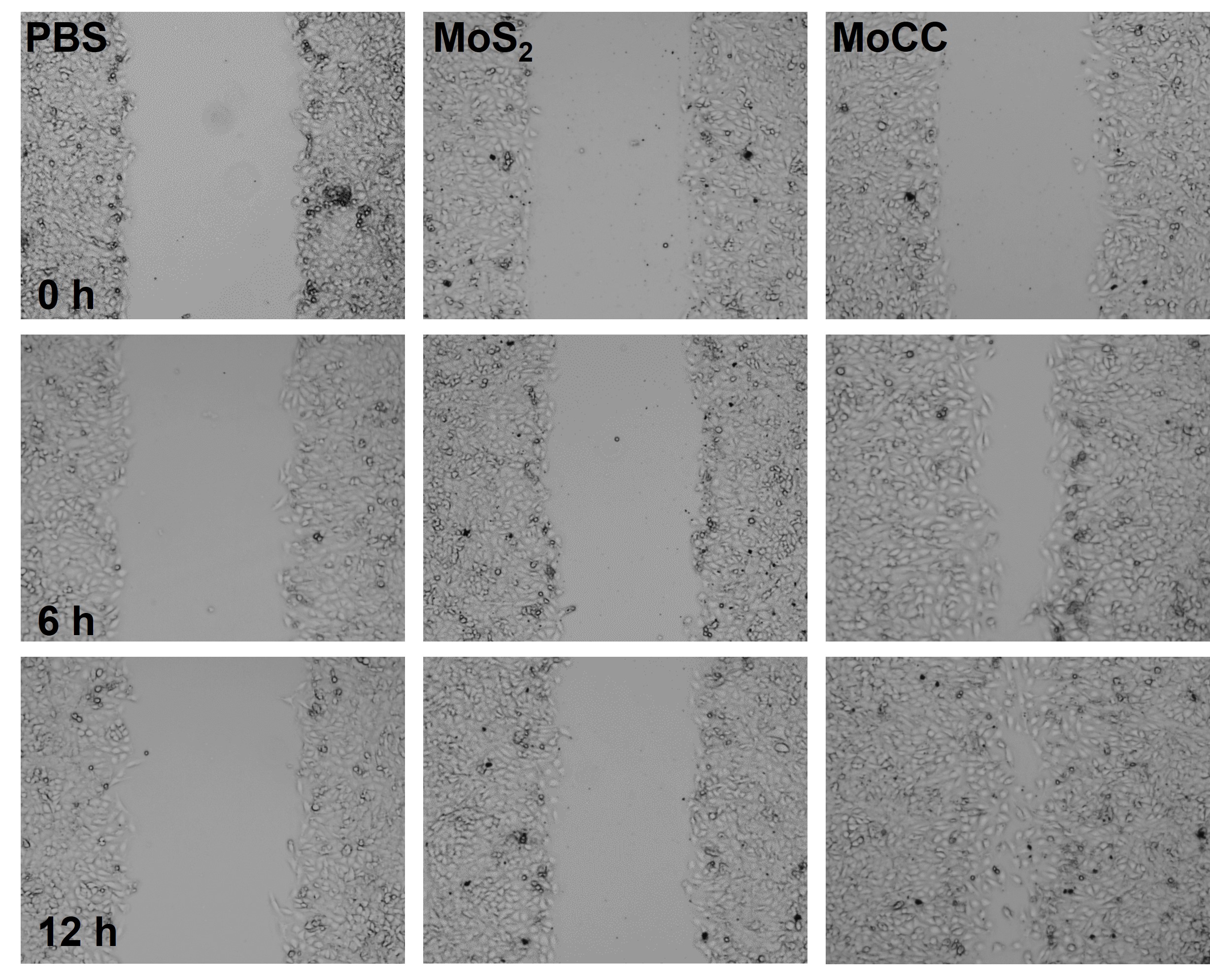


**Figure S40.** Scratch assays of HUVEC treated at 0, 6, and 12 h.


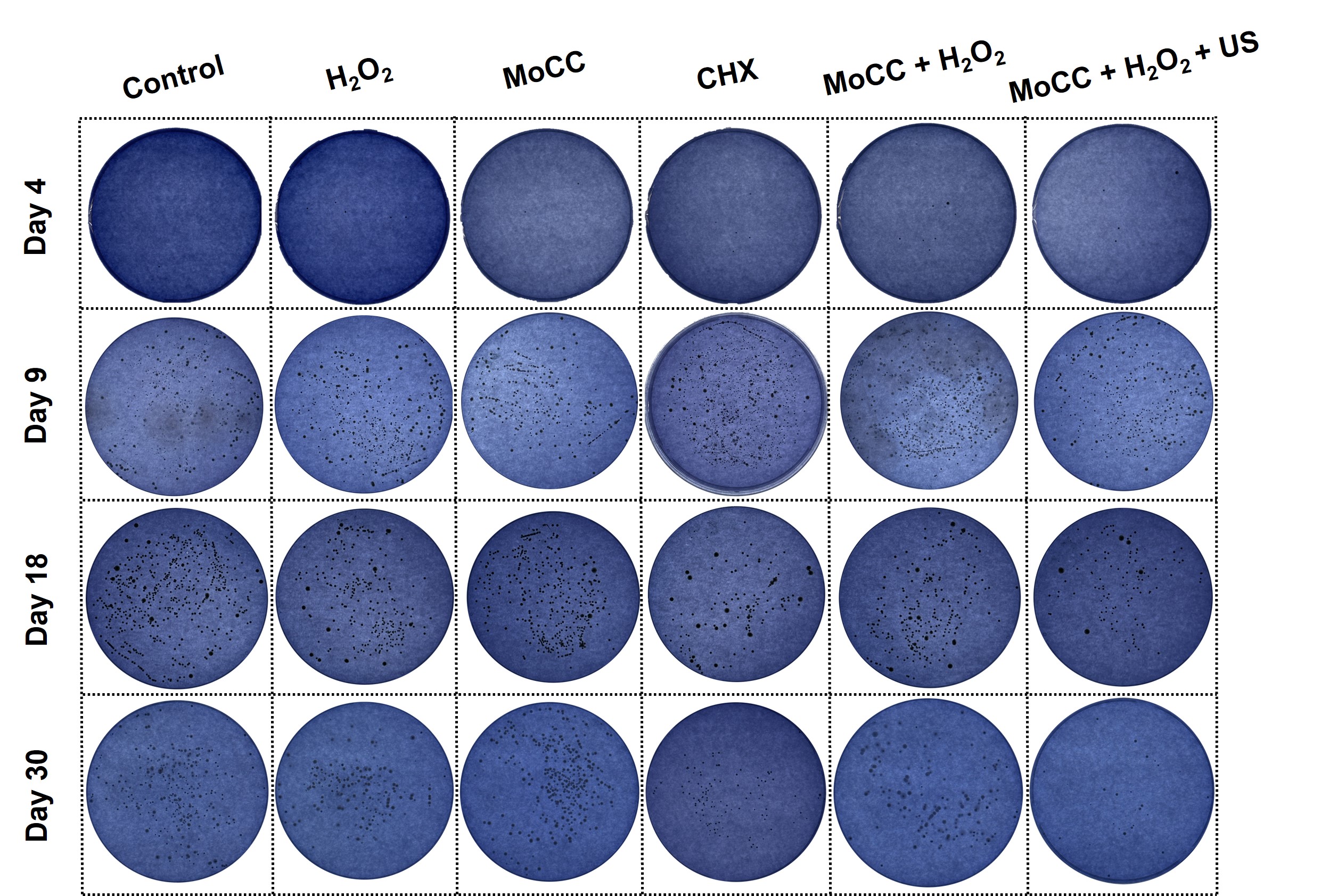


**Figure S41.** Bacterial colonies isolated from oral cavity of SD rats at different days.


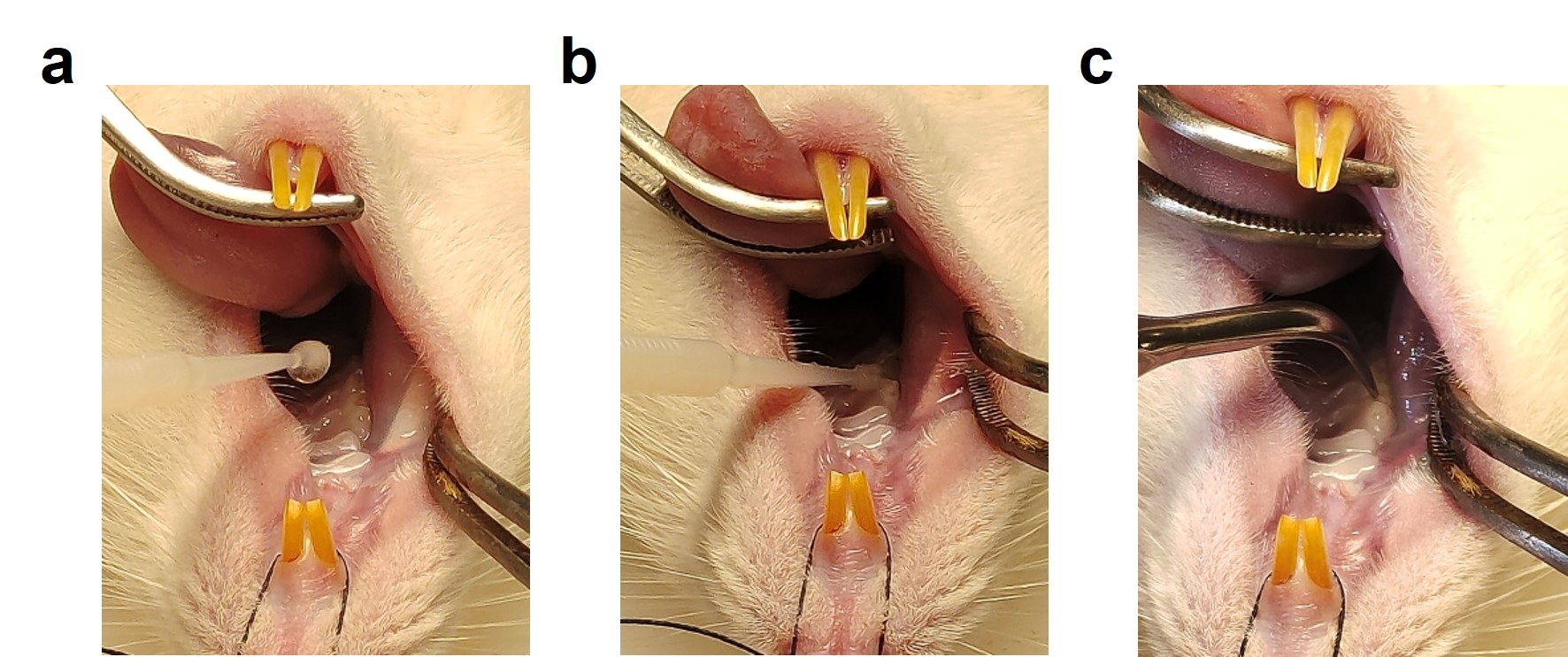


**Figure S42.** Illustration of in vivo dental caries treatments. (a) MoCC-encapsulated sodium alginate covering the molar surface. (b) Ca^2+^ crosslinked hydrogel formation. (c) Application of US to stimulate the US toothbrush in daily teeth cleaning. For MoCC + H_2_O_2_ and MoCC + H_2_O_2_ + US groups, 200 μL of 1% H_2_O_2_ was applied after the hydrogel was cross-linked. For MoCC + H_2_O_2_ + US group, an ultrasonic probe was applied for 5 min to enhance the anti-caries efficacy.


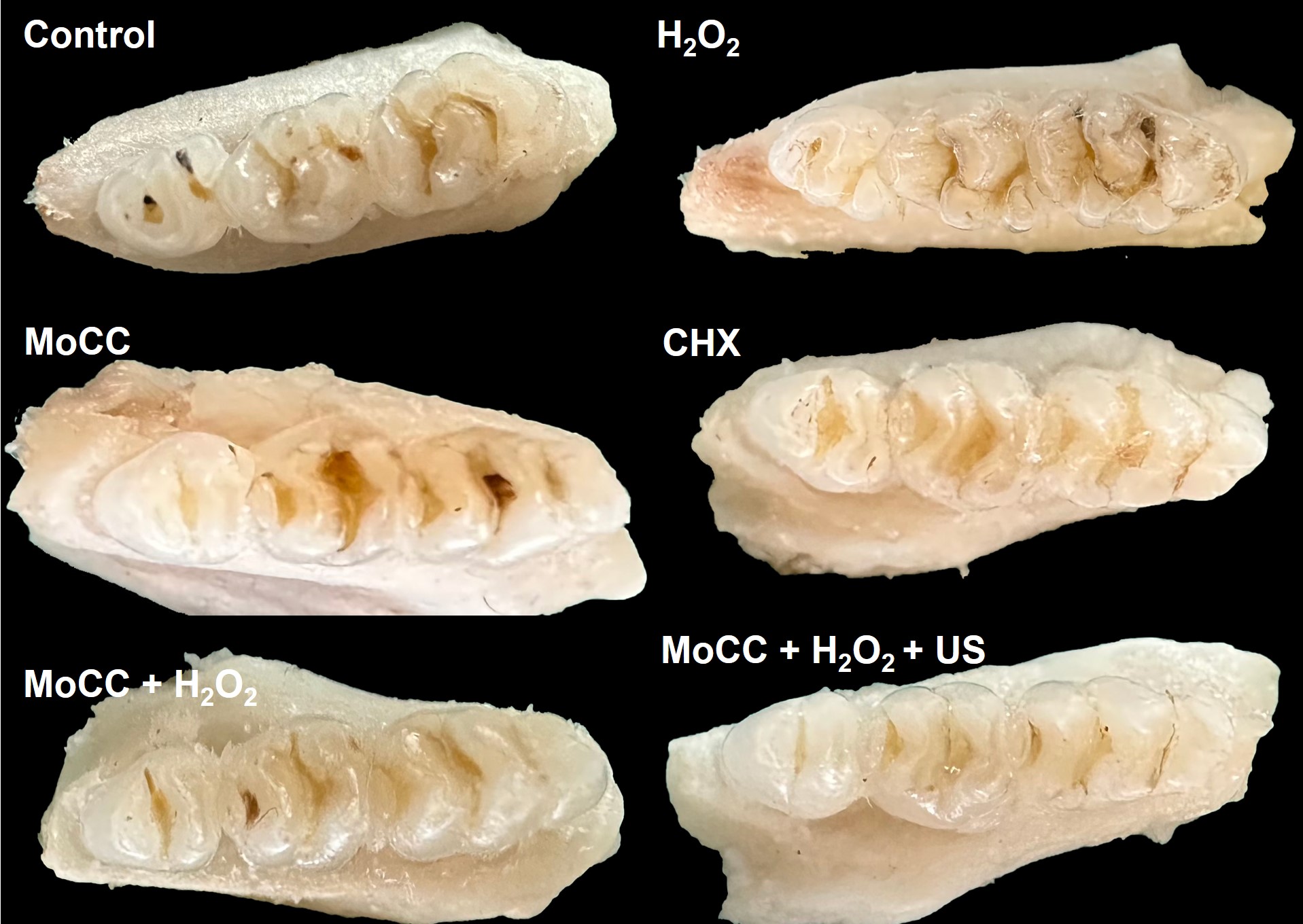


**Figure S43.** Representative photographs of the occlusal surface of rodent teeth after various treatments on day 30.


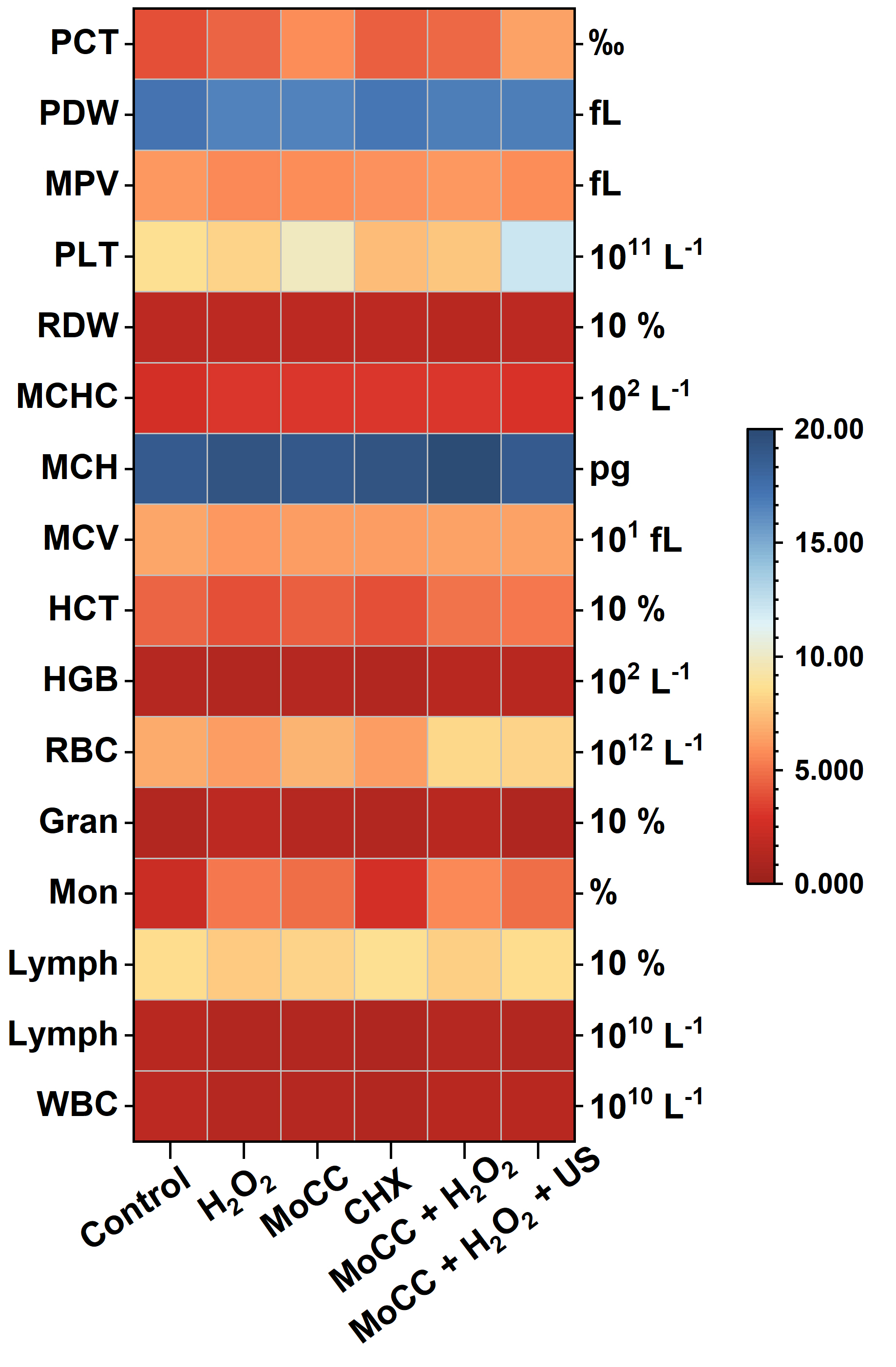


**Figure S44.** Heatmap of hematological parameters of SD rats after various treatments.


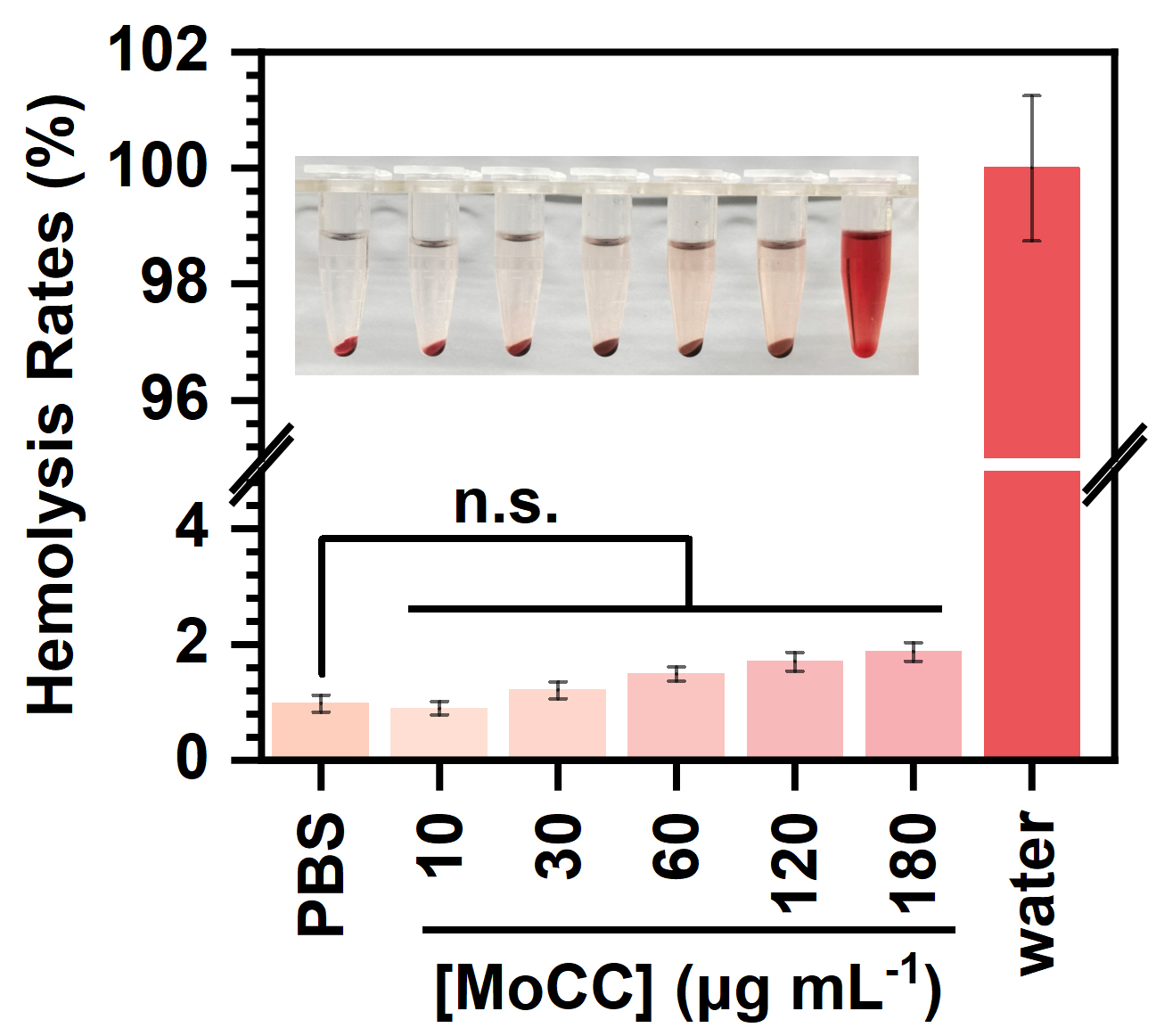


**Figure S45.** Hematolysis rates of MoCC at different concentrations. The inserted image is the corresponding photographs of PBS, MoCC with different concentrations, and water (from left to right).


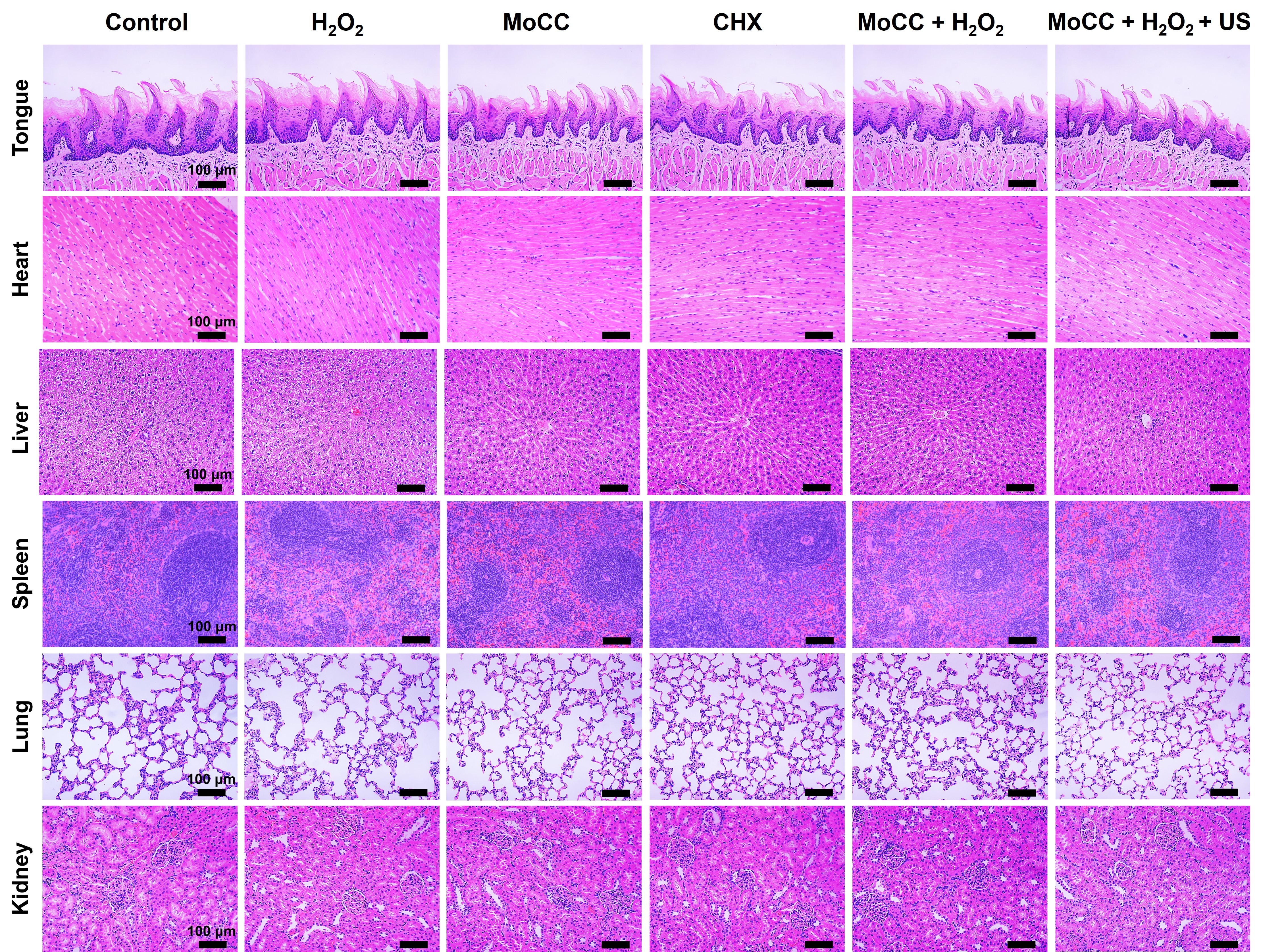


**Figure S46.** H&E staining images of main organs of SD rats after various treatments.

**Supporting References**

[1] Y. Wang, S. Wang, Y. Meng, Z. Liu, D. Li, Y. Bai, G. Yuan, Y. Wang, X. Zhang, X. Li, X. Deng, Nat. Commun. **2022**, 13, 4419.

[2] G. Kresse, D. Joubert, Phys. Rev. B **1999**, 59, 1758-1775.

[3] J. P. Perdew, K. Burke, M. Ernzerhof, Phys. Rev. Lett. **1996**, 77, 3865-3868.

[4] W. Tang, E. Sanville, G. Henkelman, J. Phys. Condens. Matter. **2009**, 21, 084204.

[5] B. Xu, H. Wang, W. Wang, L. Gao, S. Li, X. Pan, H. Wang, H. Yang, X. Meng, Q. Wu, L. Zheng, S. Chen, X. Shi, K. Fan, X. Yan, H. Liu, Angew. Chem. Int. Ed. **2019**, 58, 4911-4916.

[6] Z. Zhou, Y. Wang, F. Peng, F. Meng, J. Zha, L. Ma, Y. Du, N. Peng, L. Ma, Q. Zhang, L. Gu, W. Yin, Z. Gu, C. Tan, Angew. Chem. Int. Ed. **2022**, 61, e202115939.

[7] X. Fan, X. Wu, F. Yang, L. Wang, K. Ludwig, L. Ma, A. Trampuz, C. Cheng, R. Haag, Angew. Chem. Int. Ed. **2022**, 61, e202113833.

[8] Y. Wu, J. Wu, L. Jiao, W. Xu, H. Wang, X. Wei, W. Gu, G. Ren, N. Zhang, Q. Zhang, L. Huang, L. Gu, C. Zhu, Anal. Chem. **2020**, 92, 3373-3379.

[9] S. Li, L. Shang, B. Xu, S. Wang, K. Gu, Q. Wu, Y. Sun, Q. Zhang, H. Yang, F. Zhang, L. Gu, T. Zhang, H. Liu, Angew. Chem. Int. Ed. **2019**, 58, 12624-12631.

[10] S. Kozuch, J. M. L. Martin, ACS Catal. **2012**, 2, 2787-2794.

[11] A. Robert, B. Meunier, ACS Nano **2022**, 16, 6956-6959.

[12] D. Xu, W. Yin, J. Zhou, L. Wu, H. Yao, M. Sun, P. Chen, X. Deng, L. Zhao, Nanoscale **2023**, 15, 6686-6695.

[13] Y. Wang, K. Qi, S. Yu, G. Jia, Z. Cheng, L. Zheng, Q. Wu, Q. Bao, Q. Wang, J. Zhao, X. Cui, W. Zheng, Nano-Micro Lett. **2019**, 11, 102.

[14] Y. He, X. Niu, L. Li, X. Li, W. Zhang, H. Zhao, M. Lan, J. Pan, X. Zhang, ACS Appl. Nano Mater. **2018**, 1, 2397-2405.

[15] T. Wang, J. Feng, H. Sun, Y. Liang, T. Du, J. Dan, J. Wang, W. Zhang, Sens. Actuators, B **2023**, 379, 133249.

[16] H. Liang, F. Lin, Z. Zhang, B. Liu, S. Jiang, Q. Yuan, J. Liu, ACS Appl. Mater. Interfaces **2017**, 9, 1352-1360.

[17] T. Maity, S. Jain, M. Solra, S. Barman, S. Rana, ACS Sustainable Chem. Eng. **2022**, 10, 1398-1407.

[18] Y. Xu, J. Yan, Y. Zhu, H. Chen, C. Wu, X. Zhu, Y. Zhang, H. Li, M. Liu, S. Yao, Anal. Chem. **2022**, 94, 14642-14651.

[19] M. Solra, S. Das, A. Srivastava, B. Sen, S. Rana, ACS Appl. Mater. Interfaces **2022**, 14, 45096-45109.

[20] J. Wu, X. Zhu, Q. Li, Q. Fu, B. Wang, B. Li, S. Wang, Q. Chang, H. Xiang, C. Ye, Q. Li, L. Huang, Y. Liang, D. Wang, Y. Zhao, Y. Li, Nat. Commun. **2024**, 15, 6174.

[21] W. Zhang, M. Wang, B. Liu, H. Chen, J. Tan, Q. Meng, J. Li, B. Ding, P. a. Ma, J. Lin, Angew. Chem. Int. Ed. **2024**, 63, e202402397.

[22] W. Huang, P. Xu, X. Fu, J. Yang, W. Jing, Y. Cai, Y. Zhou, R. Tao, Z. Yang, J. Nanobiotechnol. **2023**, 21, 294.

[23] G. Yuan, C. Wang, Z. Xi, S. Li, X. Sun, P. Hang, X. Liu, J. Han, R. Guo, Small **2023**, 19, 2303739.
